# Correction: *Can India’s primary care facilities deliver? A cross-sectional assessment of the Indian public health system’s capacity for basic delivery and newborn services*

**DOI:** 10.1136/bmjopen-2017-020532corr1

**Published:** 2018-08-01

Sharma J, Leslie HH, Regan M, *et al*. Can India’s primary care facilities deliver? A cross-sectional assessment of the Indian public health system’s capacity for basic delivery and newborn services. *BMJ Open* 2018;8:e020532. doi: 10.1136/bmjopen-2017-020532.

The previous version of this manuscript contains some error in affiliation section namely the affiliation for Dr. Devaki Nambiar’s institute. It should read as:

The George Institute for Global Health

instead of

George Institute for Health, New Delhi, India

