# Supplementary figures and images for: Can India’s primary care facilities deliver? A cross-sectional assessment of the Indian public health system’s capacity for basic delivery and newborn services
Source: BMJ Open. 2018 Jun 4;8(6):e020532. doi: 10.1136/bmjopen-2017-020532 (PMC5988146; doi:10.1136/bmjopen-2017-020532)

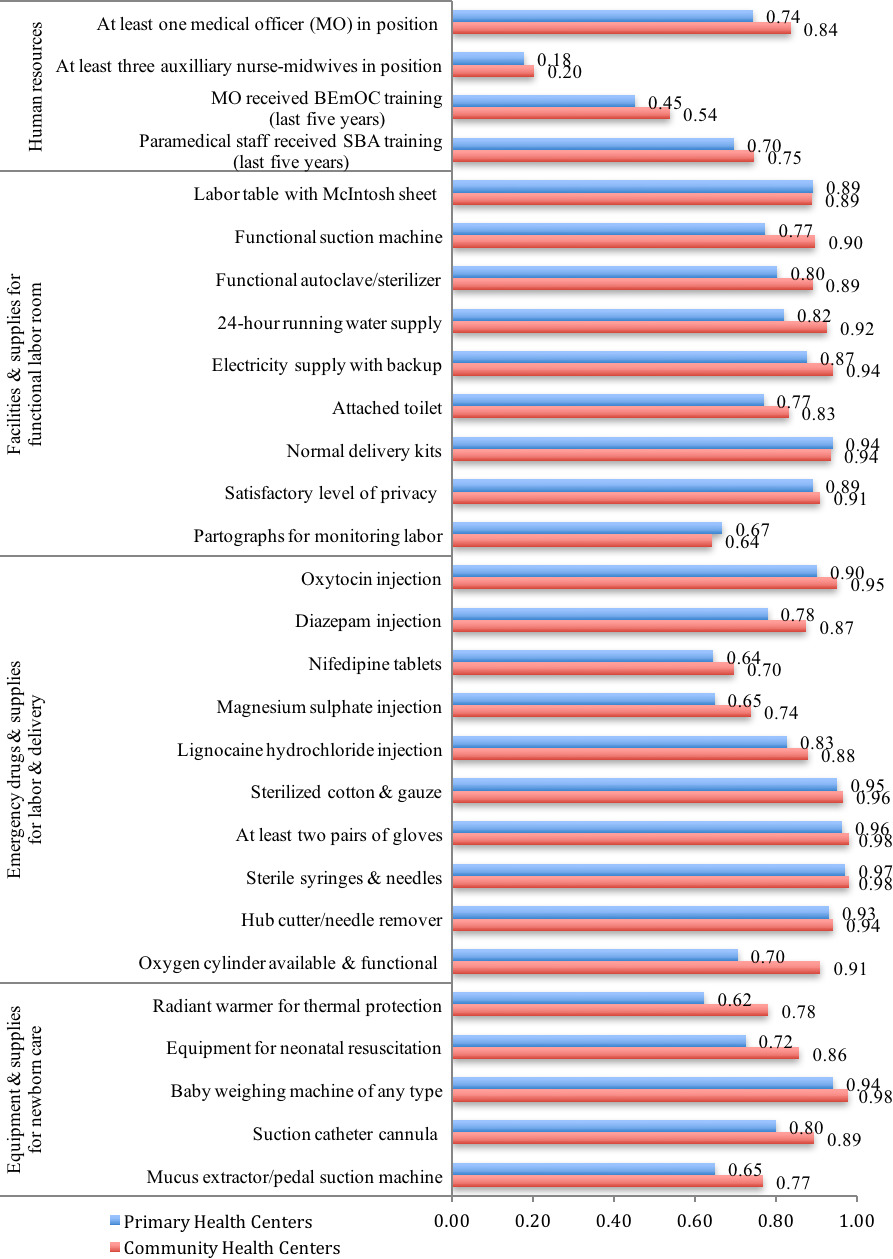

Supplement: Supplementary file 1 [file bmjopen-2017-020532supp001.jpg]

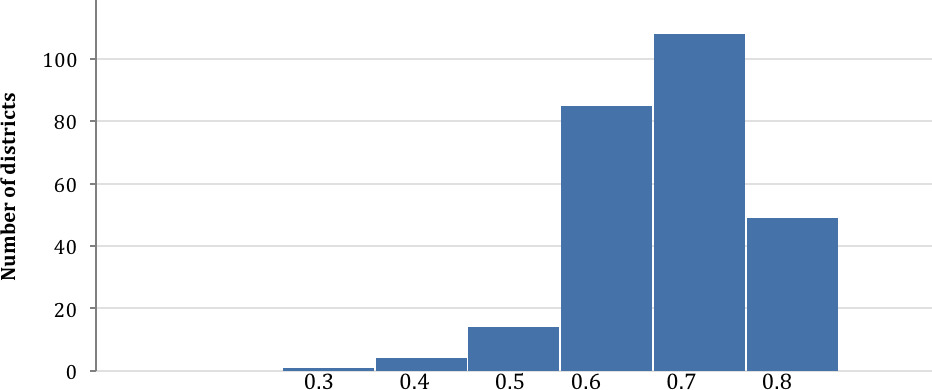

Supplement: Supplementary file 2 [file bmjopen-2017-020532supp002.jpg]

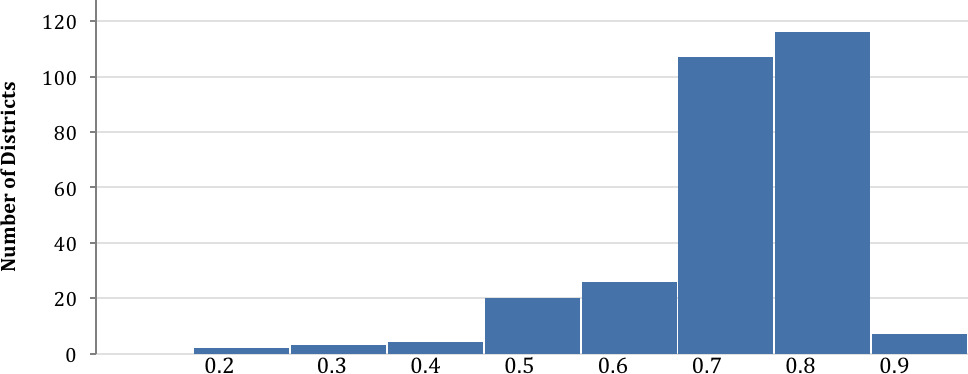

Supplement: Supplementary file 3 [file bmjopen-2017-020532supp003.jpg]
