# Supplementary material for: Can India’s primary care facilities deliver? A cross-sectional assessment of the Indian public health system’s capacity for basic delivery and newborn services
Source: BMJ Open. 2018 Jun 4;8(6):e020532. doi: 10.1136/bmjopen-2017-020532 (PMC5988146; doi:10.1136/bmjopen-2017-020532)
Supplement: Author's manuscript [file bmjopen-2017-020532.draft_revisions.pdf]

BMJ Open is committed to open peer review. As part of this commitment we make the peer review history of every article we publish publicly available.

When an article is published we post the peer reviewers' comments and the authors' responses online. We also post the versions of the paper that were used during peer review. These are the versions that the peer review comments apply to.

The versions of the paper that follow are the versions that were submitted during the peer review process. They are not the versions of record or the final published versions. They should not be cited or distributed as the published version of this manuscript.

BMJ Open is an open access journal and the full, final, typeset and author-corrected version of record of the manuscript is available on our site with no access controls, subscription charges or pay-per-view fees (http://bmjopen.bmj.com).

If you have any questions on BMJ Open's open peer review process please email info.bmjopen@bmj.com

BMJ Open

Can India's primary care facilities deliver? A cross-sectional assessment of the Indian public health system's capacity for basic delivery and newborn services

Journal:	*BMJ Open*
Manuscript ID	bmjopen-2017-020532
Article Type:	Research
Date Submitted by the Author:	09-Nov-2017
Complete List of Authors:	Sharma, Jigyasa; Harvard T. H. Chan School of Public Health, Global Health and Population<br>Leslie, Hannah; Harvard T. H. Chan School of Public Health, Global Health and Population<br>Regan, Mathilda; Harvard School of Public Health, Global Health and Population<br>Nambiar, Devaki; Public Health Foundation of India<br>Kruk, Margaret; Harvard School of Public Health,
**Primary Subject Heading**:	Health services research
Secondary Subject Heading:	Health policy, Global health, Health services research
Keywords:	Health policy < HEALTH SERVICES ADMINISTRATION & MANAGEMENT, Quality in health care < HEALTH SERVICES ADMINISTRATION & MANAGEMENT, Health Systems, Maternal Health, Newborn Health

SCHOLARONE™
Manuscripts

Can India's primary care facilities deliver? A cross-sectional assessment of the Indian public health system's capacity for basic delivery and newborn services

Jigyasa Sharma, MSc[1], Hannah H Leslie, PhD[1], Mathilda Regan, MPH[1], Devaki Nambiar, PhD[2], Margaret E Kruk, MD[1]

[1]Department of Global Health and Population, Harvard T.H. Chan School of Public Health, USA
[2]Public Health Foundation of India, India

Corresponding Author:
Jigyasa Sharma
Department of Global Health and Population
Harvard T.H. Chan School of Public Health
655 Huntington Avenue
Boston, MA 02115
jigyasa.sharma@mail.harvard.edu

For peer review only

Abstract

Objectives

Using nationally representative District Level Household and Facility Survey (1) assess input and process capacity for basic delivery and newborn (delivery care hereafter) care in the Indian public health system and (2) describe differences in facility capacity between rural and urban areas and across states.

Setting

Indian public primary care facilities (Primary Health Centers [PHCs] and Community Health Center [CHCs]).

Methods

We developed a summative index of 33 structural and process capacity items matching the Indian Public Health Standards for PHCs as a metric of minimum facility capacity for delivery care. We assessed differences in performance on this index across facility type and location.

Results

Our study sample consisted of 8536 PHCs and a census of 4810 CHC facilities. About 30% of PHCs and 5% of CHCs reported not offering any delivery care. Amongst those offering services, volumes were low: median monthly delivery volume was 8 (IQR=13) in PHCs and 41 (IQR=73) in CHCs. Basic delivery care capacity was sub-optimal. Mean facility capacity was low in PHCs in both urban (0.64) and rural (0.63) areas, while in CHCs was slightly higher in urban areas (0.77 vs. 0.74). Gaps were most striking in availability of skilled human resources and emergency obstetric services. Poor capacity facilities were more concentrated in the more impoverished states, with 37% of districts

For peer review only - http://bmjopen.bmj.com/site/about/guidelines.xhtml

from these states receiving scores in the lowest third of the facility capacity index (<0.70), compared to 21% of districts otherwise.

Conclusion

Basic delivery care capacity in Indian public primary care facilities is poor in both rural and urban areas, especially lacking in the poorest states with worst health outcomes. Improving maternal and newborn health outcomes will require focused attention to quality measurement, accountability mechanisms, and quality improvement. Policies to address deficits in skilled providers and emergency service availability are urgently required.

For peer review only

Strengths and limitations of this study

1. Uses a nationally representative survey to assess primary public health facilities' adherence to nationally set minimum recommended standards for basic delivery and newborn care.
2. The index used to assess input and process capacity is based on national guidelines, and therefore represents a contextually appropriate measure of basic service readiness.
3. However, the index lacked information on important technical and interpersonal care processes, such as provider competence, owing to data limitation.
4. Similarly, limited data constrained our ability to assess quality of referral care, and quality of care in private primary care facilities, which may perform differently on our basic delivery care capacity index.

Ethical approval:

The Harvard University Human Research Protection Program approved this analysis as exempt from human subjects review.

Funding statement

This research received no specific grant from any funding agency in the public, commercial or not-for-profit sectors.

Competing interests statement

For peer review only

For peer review only - http://bmjopen.bmj.com/site/about/guidelines.xhtml

We have read and understood BMJ policy on declaration of interests and declare that we have no competing interests.

Author contributions

JS, HHL and MEK conceived the research question. JS and HHL developed the methods, and JS, HHL and MR conducted the data analysis. JS, HHL, MR, DN and MEK interpreted the results, and JS wrote the first draft of the manuscript. JS, HHL, MR, DN and MEK edited and revised the manuscript and approved the final draft for submission

Data sharing statement

The data is publicly available through the International Institute of Population Sciences, India. Website: http://www.iipsindia.ac.in

For peer review only

Introduction

The adoption of Sustainable Development Goals (SDGs) has reaffirmed the reduction of preventable maternal and newborn deaths as global health priorities. Achieving the targeted reduction of the global maternal mortality ratio (MMR) to less than 70 per 100,000 live births and neonatal mortality to 12 per 1,000 live births [1] will require near universal coverage of institutional deliveries and timely detection and management of birth complications, as most maternal and newborn deaths occur at birth or within 24 hours of birth [2]. An emerging body of literature suggests that getting women to facilities alone will not suffice: what happens when a woman reaches a facility – the quality of care during delivery – matters [3-5].

In India, increased coverage of facility-based births has not successfully translated to improvement in health outcomes for mothers and newborns. Under India's National Rural Health Mission (NRHM), a variety of interventions were introduced through architectural improvements in the fund flow and design of services: increased number of maternal care facilities, particularly primary health centers (PHCs) and community health centers (CHCs); a strengthened supply chain for essential medicines, equipment and supplies; and Janani Surakshya Yojana (JSY), a financial-incentive program to increase institutional deliveries [6]. Following the launch of NHRM in 2005, institutional deliveries in rural areas have more than doubled [7], but without improvement in delivery outcomes [8-10]. Annual decline in neonatal mortality between 2005 and 2015 was faster than in the preceding years; however, the rate of decline in not sufficient to meet the 2030 SDG targets [11]. Inadequate quality of care, including insufficient facility readiness, and

For peer review only

low provider skill and clinical management capacity, as evidence from low and middle income (LMIC) countries indicate, could explain why increased utilization alone has not resulted in the desired reduction in adverse delivery outcomes [12, 13].

Beyond health outcomes, quality of care also affects utilization. Evidence from India indicates that the availability of a labor room and adequacy of essential equipment and laboratory services for delivery at public health facilities have a significant effect on service uptake [14, 15]. Describing and improving quality of care during delivery is relevant to improving service uptake in India, where maternal and newborn services are underutilized despite availability of primary healthcare in public health facilities free of charge.

India has made several efforts to strengthen its public health service provision. Initial policy emphasis was on rural areas given the preponderance of the Indian population living there and the assumption that population access to quality health services was relatively better in urban areas. However, mounting evidence that the health of the urban poor lagged that of the rich, and sometimes even rural populations, led to the formal launch of the National Urban Health Mission in 2013 [16]. The unified National Health Mission is now charged with oversight for public health care in urban and rural areas [17]. The implementation of programs and policies, however, has varied sub-nationally. Under the NRHM, substantial funding and technical support has been provided to states with relatively weaker public health indicators and health system infrastructure, including the Empowered Action Group (EAG) states (Bihar, Jharkhand, Chhattisgarh, Madhya

For peer review only - http://bmjopen.bmj.com/site/about/guidelines.xhtml

Pradesh, Uttar Pradesh, Uttaranchal, Orissa and Rajasthan) [18]. About 46% of India's population resides in the EAG states, and this region lags behind the rest of the Indian states on socio-economic, demographic and health indicators.

Public health facilities are a significant provider of care in India, especially for rural and vulnerable population segments. About 80.1% of all deliveries in rural India are facility-based, of which about 70% are in public facilities. For urban areas, 89.5% of births are institutional, 47.4% of which are in public facilities [19]. In most of the Indian states (25/30), more facility-based deliveries happen at government health institutions than in private facilities [20, 21]. Additionally, quality of care in the public sector affects the poorest segment of the population the most [22], as the poorest wealth quintile class is more heavily reliant on public health facilities than the richest, in both rural (58% vs. 29%) and urban areas (48% vs. 19%).

To boost the performance of public health facilities, in 2007 the government introduced the Indian Public Health Standards (IPHS), a set of recommendatory standards to be used as a reference for public health infrastructure planning and as a benchmark for assessing the functional status of health facilities [23]. Although widely used in health system planning and management, the use of these standards in health system research has been limited in number and geographic scope to date. Rural Health Statistics from 2015 note that only 21% of PHCs and 26% of CHCs were functioning in concordance with IPHS standards, although a service-specific breakdown was not provided [24]. A few studies have utilized IPHS as a reference, noting deficiencies in service availability, human

For peer review only - http://bmjopen.bmj.com/site/about/guidelines.xhtml

resource and infrastructure [25-28]. With the exception of one [28], all these studies surveyed less than a hundred facilities.

In this paper, using the IPHS (2007) as the minimum standard for basic facility capacity for delivery and newborn care (hereafter, delivery care), we (1) assess the performance of PHCs and CHCs in India against this standard; and (2) describe differences in delivery care capacity between urban and rural areas and across states.

Methods

Study sample

We drew data from the fourth cycle of the District Level Household and Facility Survey (DLHS) conducted by the International Institute of Population Sciences in India. We used data from the facility modules of the DLHS for PHCs and CHCs to capture the primary level of the public health system designated to provide delivery services. Typically, PHCs serve a population of 20,000 to 30,000 [29], and CHCs, which are intended to be referral centers for four PHCs, cater to approximately a population of 80,000 to 120,000 [30]. Within the tiers of the Indian public health system, CHCs are referral centers for PHCs; yet they are frequently used for first-line delivery care. In this paper, in keeping with international guidelines, both are referred to as primary care facilities [31].

The most recent survey, DLHS-4, was conducted in 2011-2012 and contains a sample of PHCs designed to be representative at district level and a census of CHCs. We also obtained the most recent district and state boundaries from the database of global

For peer review only

administrative areas (GADM.org) and matched DLHS data to the appropriate area using state and district name. We were unable to match three districts, likely due to district boundary changes between 2013 and 2016.

Basic delivery care capacity index

Availability of essential physical and human resources and services are foundational to provision of high-quality care.[32] The study objective was to evaluate basic delivery care capacity that should be present in all delivery facilities. We used the IPHS for PHCs to define this capacity; CHCs are assumed to meet at least the standards of the lower-tier PHCs [29]. The facility capacity index comprises structure and process capacity elements required for both routine and basic emergency obstetric care.

We identified 48 elements of the IPHS pertaining to delivery care. Of these, we found at least some information on 33 in the DLHS facility audit, of which 26 were a complete match. Facility capacity to offer services such as manual removal of placenta, appropriate pre-referral management of and referral for obstetric emergencies, and availability of select drugs and equipment, identified as the basic requirements for all PHCs and higher-level facilities, were not included in the facility audit. See Table 1 for the full list of IPHS requirements along with corresponding item from DLHS survey.

Our data comprised both observed and self-reported measures of structural inputs to care and self-reported process capacity indicators. Structural inputs fell into four sub-domains: skilled provider availability (average of 4 indicators), facilities for a functional labor

For peer review only - http://bmjopen.bmj.com/site/about/guidelines.xhtml

room (average of 9 indicators), emergency drugs and supplies for labor (average of 10 indicators), and delivery and newborn care equipment and supplies (average of 5 indicators). Process capacity indicators included reports from survey respondent (facility managers or medical officers in charge) on availability of assisted vaginal deliveries; administration of parenteral oxytocins, antibiotics, magnesium sulfate; postpartum hemorrhage management; newborn resuscitation and thermal protection services. All process capacity indicators were binary measures. The items were grouped following the IPHS categorization. See Supplemental Figure 1 for details on all items.

For both structure (4 sub-domains) and process (7 items) capacity indices, sub-domains or indicators were averaged to provide a facility summary score from 0 to 1, with missing values excluded. Missing values were minimal (less than 3% of facilities) for all indicators except for availability of newborn resuscitation (11% of PHCs missing) and electricity supply with backup (23.6% of PHC and 9.6% of CHC missing). We created an index of facility capacity for basic delivery care as an average of the structure and process capacity indices. We followed current practice and weighted each indicator equally within a sub-domain or index given the lack of guidance in the IPHS on weights [33, 34].

Covariates

We identified covariates that may be associated with facility capacity. We followed DLHS classification of facilities as either a PHC or CHC, which is based on delivery volume and their capacity to perform comprehensive obstetric emergency management.

For peer review only - http://bmjopen.bmj.com/site/about/guidelines.xhtml

We categorized facilities by rural versus urban location as reported in the survey. We also calculated annual delivery volume based on facility report, excluding missing values. We calculated capacity for quality delivery care within districts by weighting each facility by delivery volume. For weighting, we assigned facilities reporting delivery capacity but missing delivery volume data or reporting no deliveries a delivery volume of 1.

Statistical analysis

In this analysis, to describe primary level public health facilities' capacity to provide basic care during delivery, we first calculated availability of basic delivery care services at the facility level: facilities not offering any services, facilities offering round-the-clock services, and those providing daytime services only. Some PHCs and CHCs may have curtailed services possibly due to low volume (resulting from proximity to a higher-level facility), inadequate infrastructure or human resources. As our data do not support classification of these facilities based on the reason for service unavailability (which may have been justified or not, given individual circumstances), we restricted subsequent analysis to facilities reporting provision of either round-the-clock or daytime only labor and delivery services. In this sample, we also assessed each facility's adherence to national standard for care provision, stratified by facility type (PHC vs. CHC).

We described variation in facility level capacity across rural and urban settings. We employed Pearson's chi-square test for binomial and Student's T-test for continuous indicators to assess the statistical significance ($p<0.05$) of difference in capacity between PHCs and CHCs and between rural and urban PHCs. To visualize differences in delivery

For peer review only - http://bmjopen.bmj.com/site/about/guidelines.xhtml

care capacity across India, we also calculated averages of overall facility capacity for delivery care at the district level, weighting facilities by total deliveries. For mapping, we divided the overall facility capacity index scores into sixths, designating the lowest third of scores as "poor capacity."

Statistical analysis was done using Stata 14.1 (StataCorp, Texas) and mapping using QGIS Version 2.12 (Free Software Foundation, Massachusetts).

Ethical approval

The Harvard University Human Research Protection Program approved this analysis as exempt from human subjects review.

Results

The 2012-2013 DLHS-4 facility survey was conducted across India; however, data are publicly available only for 30 out of 36 states and union territories (excluding the states of Gujarat, and Jammu and Kashmir, and the territories Dadar and Nagar Haveli, Daman Diu, the National Capital Territory of Delhi, and Lakshadweep). Our study sample comprised of a total of 8536 PHCs and 4810 CHCs. Although all PHCs and CHCs are expected to provide delivery care, about 30.2% (N=2557) of PHCs and 5.2% (N=251) of CHCs reported not providing any delivery services, either round-the-clock or daytime-only. Only 59.7% (N=4798) of rural and 62.7% (N=312) of urban PHCs and only 92.7% (N=3578) of rural and 94.9% (N=895) of urban CHCs offered 24/hour delivery and

For peer review only

newborn services. As Figure 1 indicates, availability of services was similar for both levels of facility across urban and rural settings.

Within facilities offering any delivery services (N= 5959 PHC, N= 4553 CHC), median monthly delivery volume was 8 (IQR=13) in PHCs and 41 (IQR=73) in CHCs. 73.4% of the PHCs self-identified as government designated 24x7 delivery facilities; 24x7 delivery and newborn services were offered in 86% and 98% of all included PHCs and CHCs, respectively. A designated area for newborn care was present only in 67% and 80% of included PHCs and CHCs, respectively.

Overall capacity for basic delivery services was sub-optimal in both PHCs (mean 0.63, standard deviation [SD] 0.23) and CHCs (mean 0.75, SD 0.17) included in this analysis. The structural capacity index for basic delivery care was slightly better than the process capacity index for both PHCs (mean 0.67, SD 0.19 vs. 0.60, SD 0.29) and CHCs (mean 0. 76, SD 0.14 vs. 0.73, SD 0.22). As shown in Figure 2, PHCs lagged CHCs on all indicators; the difference between PHCs and CHCs was statistically significant for all indicators and the summary index. Although CHCs, as the referral centers for PHCs, are expected to offer more comprehensive care, they frequently lacked basic infrastructure required even at PHCs. Human resource availability and training in both CHCs (mean 0.58, SD 0.23) and PHCs (mean 0.52, SD 0.26) were comparably low. Particularly large gaps were seen in process capacity indicators such as provision of assisted vaginal deliveries (13% of PHCs versus 30% of CHCs), administration of parenteral magnesium

For peer review only - http://bmjopen.bmj.com/site/about/guidelines.xhtml

sulfate (54% versus 70%), and management of postpartum hemorrhage (53% of PHCs versus 69% of CHCs).

Figures 3a and 3b display the differences in structural and process capacity indices, and in overall capacity of basic delivery care across rural and urban settings, for PHCs and CHCs, respectively. Facility capacity was low across the board, with small differences across geographical settings. Scores for structural, process, and overall capacity summary indices were comparable for PHCs, with no significant rural-urban differences. Rural-urban differences in CHCs were slightly more pronounced, with urban CHCs outperforming rural CHCs by 0.02 to 0.04 in structural, process, and overall facility capacity indices.

As shown in Figure 4, the capacity of facilities that offered delivery care varied across the country, with districts with the poorest facility capacity concentrated in the northern part of the country. Seven districts had no facilities offering labor and delivery services. We had no data for 118 districts, including the districts in regions for which survey data was not publicly available. In both EAG and non-EAG states there was significant variation in facility capacity (Supplemental Figures 2a and 2b). There was a disproportionate number of poor capacity facilities in EAG states, with 37% of districts scoring in the lowest third of the facility capacity index (less than 0.70) compared to 21% of non-EAG states. Among the nine EAG states, Uttar Pradesh had the greatest concentration of poor capacity facilities, with 84% percent of districts receiving scores in the lowest third.

For peer review only - http://bmjopen.bmj.com/site/about/guidelines.xhtml

Discussion

Using a national health facility survey, we found that Indian public primary care facilities fail to meet the nationally set minimum recommended standards for basic delivery care. Despite a concerted effort to increase accessibility, about 30% of PHCs and 5% of CHCs did not offer any delivery and newborn services; round-the-clock delivery and newborn care services were available only in 60% of PHCs and 94% of CHCs. Both PHCs and CHCs offering these services had critical deficiencies in routine and emergency care practices, infrastructure and staffing, fulfilling an average of only 63% and 75% of an index of basic delivery care capacity, respectively. These findings are consistent with studies documenting weak emergency care capacity, infrastructure and staffing in low-income countries, particularly in lower level facilities with low delivery volume [33, 35]. Most CHCs, which are meant to serve as referral centers to manage more complicated cases [18], failed to meet the threshold set for lower level facilities. Furthermore, monthly delivery volume in PHCs was very low, suggesting inefficiencies in the health system, considering the current amount of resources spent at this level of healthcare [36]. This merits attention for effective planning and resource allocation as healthcare delivery structure being developed for urban India under the National Urban Health Mission follows the same model as in rural areas [17].

Structural capacity across the study facilities was marginally better than process capacity, with large gaps in provision of signal functions of emergency obstetric care [37]. For example, assisted vaginal deliveries were offered only in about one-tenth of PHCs and one-third of CHCs. Almost one-half of PHCs and one-third of CHCs reported not

For peer review only

providing services to manage postpartum hemorrhage, a leading cause of maternal mortality in India. We also noted that availability of medicine and supplies, such as the partograph or uterotonics, was worse than that of equipment and infrastructure, such as scales or labor tables, underscoring the need to strengthen the supply chain for essential medicine and supplies. This is a challenge that NRHM was designed to address, and one that has been resistant to change [15].

Similarly concerning are the low scores on human resource availability and training across facilities. Although addressing the shortage of skilled providers in rural PHCs and CHCs has also been one of the agendas of the NRHM, critical deficiencies clearly persist, as has been documented elsewhere [38]. The gaps observed are especially problematic because our indicators reflect a minimum level of human resource preparedness (for example, skilled birth attendance training for auxiliary nurse-midwives or emergency obstetric care training for medical officers). They do not include key dimensions of provider availability and competence, which the World Health Organization identifies as integral to good quality of care during facility-based deliveries [39]. For example, accounting for absenteeism, a common problem in public facilities in India, would probably lower our estimate of actual human resource availability significantly [38].

Facility capacity for basic delivery care in public facilities was similar in rural and urban areas. Our findings underline the need to improve facility capacity in both urban and rural areas, which the unified National Health Mission may be well situated to tackle. Given the fact that the public facilities assessed here are the main source of obstetric care for

For peer review only

poor populations in both rural and urban settings, [40, 41] the deficiencies we found are likely to disproportionately affect the most vulnerable women. Meeting the commitment to equitable care inherent in the National Health Mission requires redoubled attention on care quality in the public health system.

Despite additional funding and considerable technical support for improving maternal and newborn health outcomes in the EAG states, facility capacity for delivery care remains weaker than in non-EAG states. We found that facilities with poor capacity were especially common in Uttar Pradesh and Bihar, states with the two highest maternal and newborn mortality rates in the country. Roughly comprising a third of India's population, these two states are critical in India's progress towards meeting national and international maternal and newborn mortality reduction targets.

We found important gaps in data and measurement of facility capacity in India. For fifteen out of forty-eight delivery care items on the IPHS, we did not find any corresponding survey question in the DLHS facility audit. Harmonizing data collection with national standards is a critical first step towards addressing information gaps. Likewise, expanding data collection to include a more diverse range of indicators to capture the nature and content of care being provided is important for understanding quality of care [42]. Examples include the use of basic emergency care procedures for mothers and newborns (signal functions) and adherence to global guidelines for routine deliveries [43]. Strengthening the publicly available health information system in India to collect reliable, frequent and timely data on facility quality is key for effective program

For peer review only - http://bmjopen.bmj.com/site/about/guidelines.xhtml

implementation and for meeting national and global targets such as those of the Ending Preventable Maternal Mortality, the Every Newborn Action Plan, and the SDGs.

Our study has several limitations. First, it would have been ideal to examine the quality of clinical care provided to women in public health facilities using a multidimensional index such as the quality of the process of intrapartum and immediate postpartum care index [34]. Our index was constructed from indicators of basic equipment and service availability, measuring facility level inputs to care and basic service readiness, but it lacked information on important technical and interpersonal care processes, such as provider competence. Previous studies have noted the importance of provider knowledge and effort in determining the quality of actual clinical encounters [44] as well as substantial variation in the skill and competence of skilled birth attendants [45]. Likewise, although disrespectful care in delivery is common [46], and respectful care is identified as WHO as a key requirement for improving quality of care during delivery [39], we could not assess interpersonal care aspects in our index. Critically, data constrained our ability to assess facility capacity in private facilities, which are also relied on for delivery care and may have different adherence to IPHS standards. We were also not able to assess quality of referral care or the outcomes of care. Thus, our measure should be seen as a starting point in measuring care quality in India; process and outcome quality may well be worse than the largely input-based measures examined here.

Our study findings add to the growing body of literature documenting gaps in provision of quality delivery care in LMICs. Moreover, these data highlight key deficiencies in

For peer review only - http://bmjopen.bmj.com/site/about/guidelines.xhtml

quality measurement in India. In future, facility assessments should collect data on process and outcome domains of quality, particularly the clinical effectiveness of care, patient experience, respectful care, and care outcomes. A variety of methods are available for this, including patient and provider interviews, direct observations, and clinical vignettes, in both the public and the private sector [44]. It is also essential to link quality measures to health outcomes and demographic characteristics of the population to assess the determinants of quality and the impact of quality on outcomes. Health systems research in India needs to be expanded to address the large knowledge gaps in health system quality [47].

Conclusion

Primary care facilities in India are not well prepared to provide high quality obstetric and newborn care, and facility capacity is worst in states with the worst health outcomes. Over the past decade, India's health system has operated in an extremely resource-constrained environment: from 2004-2014, government health expenditure has remained approximately around 1% of country's GDP [22]. The Indian government will need to increase investment in the health system, in providers, and in research to harness the full benefit of its public health infrastructure. Improving quality of care and strengthening public health infrastructure is integral to India's path to universal health coverage: without an explicit focus on quality, a push towards universal coverage is unlikely to lead to better health for mothers and newborns.

For peer review only - http://bmjopen.bmj.com/site/about/guidelines.xhtml

References

1. UN Development Program. Sustainable Development Goals. Geneva, United Nations: 2015.
2. The Partnership for Maternal Newborn & Child Health. A Global Review of the Key Interventions Related to Reproductive, Maternal, Newborn and Child Health (RMNCH). Geneva, Switzerland: PMNCH: 2011.
3. Kruk ME, Larson E, Twum-Danso NA. Time for a quality revolution in global health. Lancet Glob Health. 2016;4(9):e594-6. doi: 10.1016/S2214-109X(16)30131-0. PubMed PMID: 27539798.
4. Dickson KE, Kinney MV, Moxon SG, Ashton J, Zaka N, Simen-Kapeu A, et al. Scaling up quality care for mothers and newborns around the time of birth: an overview of methods and analyses of intervention-specific bottlenecks and solutions. BMC Pregnancy Childbirth. 2015;15 Suppl 2:S1. doi: 10.1186/1471-2393-15-S2-S1. PubMed PMID: 26390820; PubMed Central PMCID: PMCPMC4578819.
5. Bhutta ZA, Das JK, Bahl R, Lawn JE, Salam RA, Paul VK, et al. Can available interventions end preventable deaths in mothers, newborn babies, and stillbirths, and at what cost? Lancet. 2014;384(9940):347-70. doi: 10.1016/S0140-6736(14)60792-3. PubMed PMID: 24853604.
6. Nandan D. National rural health mission: turning into reality. Indian J Community Med. 2010;35(4):453-4. doi: 10.4103/0970-0218.74338. PubMed PMID: 21278861; PubMed Central PMCID: PMCPMC3026119.
7. National Health Mission. Five years (2009–2014). Achievements and new initiatives. New Delhi: Ministry of Health and Family Welfare, Government of India, 2014.
8. Lim SS, Dandona L, Hoisington JA, James SL, Hogan MC, Gakidou E. India's Janani Suraksha Yojana, a conditional cash transfer programme to increase births in health facilities: an impact evaluation. Lancet. 2010;375(9730):2009-23. doi: 10.1016/S0140-6736(10)60744-1. PubMed PMID: 20569841.
9. Powell-Jackson T, Mazumdar S, Mills A. Financial incentives in health: New evidence from India's Janani Suraksha Yojana. J Health Econ. 2015;43:154-69. doi: 10.1016/j.jhealeco.2015.07.001. PubMed PMID: 26302940.
10. Ng M, Misra A, Diwan V, Agnani M, Levin-Rector A, De Costa A. An assessment of the impact of the JSY cash transfer program on maternal mortality reduction in Madhya Pradesh, India. Glob Health Action. 2014;7:24939. doi: 10.3402/gha.v7.24939. PubMed PMID: 25476929; PubMed Central PMCID: PMCPMC4256523.
11. Million Death Study C. Changes in cause-specific neonatal and 1-59-month child mortality in India from 2000 to 2015: a nationally representative survey. Lancet. 2017. doi: 10.1016/S0140-6736(17)32162-1. PubMed PMID: 28939096.
12. Leslie HH, Fink G, Nsona H, Kruk ME. Obstetric Facility Quality and Newborn Mortality in Malawi: A Cross-Sectional Study. PLoS Med. 2016;13(10):e1002151. doi: 10.1371/journal.pmed.1002151. PubMed PMID: 27755547; PubMed Central PMCID: PMCPMC5068819.
13. Knight HE, Self A, Kennedy SH. Why are women dying when they reach hospital on time? A systematic review of the 'third delay'. PLoS One. 2013;8(5):e63846. doi:

For peer review only - http://bmjopen.bmj.com/site/about/guidelines.xhtml

10.1371/journal.pone.0063846. PubMed PMID: 23704943; PubMed Central PMCID: PMCPMC3660500.
14. Patel R, Ladusingh L. Do Physical Proximity and Availability of Adequate Infrastructure at Public Health Facility Increase Institutional Delivery? A Three Level Hierarchical Model Approach. PLoS One. 2015;10(12). doi: 10.1371/journal.pone.0144352. PubMed PMID: 26689199; PubMed Central PMCID: PMCPMC4686327.
15. Kumar S, Dansereau E. Supply-side barriers to maternity-care in India: a facility-based analysis. PLoS One. 2014;9(8):e103927. Epub 2014/08/06. doi: 10.1371/journal.pone.0103927. PubMed PMID: 25093729; PubMed Central PMCID: PMCPMC4122393.
16. Dasgupta R, Bisht R. The Missing Mission in Health. Economic and Political Weekly. 2010;XLV(6):3.
17. Bhaumik S. India outlines plans for National Urban Health Mission. Lancet. 2012;380(9841):550. PubMed PMID: 22891372.
18. Programme Evaluation Organisation. Evaluation Study of National Rural Health Mission (NRHM) in Seven States. New Delhi, India: Planning Commission, Government of India, 2011.
19. National Sample Survey Office, Ministry of Statistics and Programme Implementation. Key Indicators of Social Consumption in India: Health. New Delhi, India: Government of India, 2014.
20. Office of the Registrar General & Census Commissioner, Institute of Economic Growth. Annual Health Survey Report. New Delhi, India: Ministry of Home Affairs, Government of India, 2015.
21. International Institute for Population Sciences. District Level Household and Facility Survey - 4 State Fact Sheet (2012-13). Mumbai: Ministry of Health and Family Welfare, Government of India, 2014.
22. Patel V, Parikh R, Nandraj S, Balasubramaniam P, Narayan K, Paul VK, et al. Assuring health coverage for all in India. Lancet. 2015;386(10011):2422-35. doi: 10.1016/S0140-6736(15)00955-1. PubMed PMID: 26700532.
23. National Health Mission, Ministry of Health & Family Welfare, Government of India. Indian Public Health Standards New Delhi, India: Government of India; [cited 2016 November 15]. Available from: http://nrhm.gov.in/nhm/nrhm/guidelines/indian-public-health-standards.html.
24. Ministry of Health and Family Welfare. Rural health statistics bulletin 2015. New Delhi: Government of India, 2015.
25. Zaman FA, Laskar NB. An application of Indian public health standard for evaluation of primary health centers of an EAG and a Non-EAG state. Indian journal of public health. 2010;54(1):36-9. Epub 2010/09/23. doi: 10.4103/0019-557x.70551. PubMed PMID: 20859050.
26. Sodani P, Sharma K. Assessing Indian Public Health Standards for 24 × 7 primary health centers: A case study with special reference to newborn care services. The Journal of National Accreditation Board for Hospitals & Healthcare Providers. 2014;1(1):12-6. doi: 10.4103/2348-6139.132919.
27. Sodani PR, Sharma K. Assessing Indian public health standards for community health centers: a case study with special reference to essential newborn care services.

For peer review only - http://bmjopen.bmj.com/site/about/guidelines.xhtml

Indian journal of public health. 2011;55(4):260-6. Epub 2012/02/03. doi: 10.4103/0019-557x.92402. PubMed PMID: 22298134.
28. Sodani PR, Sharma K. Strengthening Primary Level Health Service Delivery: Lessons from a State in India. Journal of Family Medicine and Primary Care. 2012;1(2):127-31. doi: 10.4103/2249-4863.104983. PubMed PMID: 24479021; PubMed Central PMCID: PMCPMC3893968.
29. Directorate General of Health Services. Indian Public Health Standards (IPHS) Guidelines for Primary Health Centers. New Delhi: Ministry of Health and Family Welfare, Government of India, 2012.
30. Directorate General of Health Services. Indian Public Health Standards (IPHS) Guidelines for Community Health Centers. New Delhi: Ministry of Health and Family Welfare, Government of India, 2012.
31. Bhatia M. The Indian Helath Care System, 2015. United States: The Commonwealth Fund, 2016.
32. Tuncalp, Were WM, MacLennan C, Oladapo OT, Gulmezoglu AM, Bahl R, et al. Quality of care for pregnant women and newborns-the WHO vision. BJOG. 2015;122(8):1045-9. doi: 10.1111/1471-0528.13451. PubMed PMID: 25929823; PubMed Central PMCID: PMCPMC5029576.
33. Kruk ME, Leslie HH, Verguet S, Mbaruku GM, Adanu RM, Langer A. Quality of basic maternal care functions in health facilities of five African countries: an analysis of national health system surveys. Lancet Glob Health. 2016;4(11):e845-e55. doi: 10.1016/S2214-109X(16)30180-2. PubMed PMID: 27670090.
34. Tripathi V, Stanton C, Strobino D, Bartlett L. Development and Validation of an Index to Measure the Quality of Facility-Based Labor and Delivery Care Processes in Sub-Saharan Africa. PLoS One. 2015;10(6):e0129491. doi: 10.1371/journal.pone.0129491. PubMed PMID: 26107655; PubMed Central PMCID: PMCPMC4479466.
35. Hsia RY, Mbembati NA, Macfarlane S, Kruk ME. Access to emergency and surgical care in sub-Saharan Africa: the infrastructure gap. Health Policy Plan. 2012;27(3):234-44. doi: 10.1093/heapol/czr023. PubMed PMID: 21441566.
36. Prinja S, Gupta A, Verma R, Bahuguna P, Kumar D, Kaur M, et al. Cost of Delivering Health Care Services in Public Sector Primary and Community Health Centres in North India. PLoS One. 2016;11(8):e0160986. doi: 10.1371/journal.pone.0160986. PubMed PMID: 27536781; PubMed Central PMCID: PMCPMC4990301.
37. WHO, UNFPA, UNICEF, Mailman School of Public Health. Monitoring emergency obstetric care: a handbook. Geneva: World Health Organization, 2009.
38. Rao M, Rao KD, Kumar AK, Chatterjee M, Sundararaman T. Human resources for health in India. Lancet. 2011;377(9765):587-98. doi: 10.1016/S0140-6736(10)61888-0. PubMed PMID: 21227499.
39. World Health Organization. Standard for improving quality of maternal and newborn care in health facilities. Geneva, Switzerland: World Health Organization Press; 2016.
40. Balarajan Y, Selvaraj S, Subramanian SV. Health care and equity in India. Lancet. 2011;377(9764):505-15. doi: 10.1016/S0140-6736(10)61894-6. PubMed PMID: 21227492; PubMed Central PMCID: PMCPMC3093249.

For peer review only

41. Barik D, Thorat A. Issues of Unequal Access to Public Health in India. Front Public Health. 2015;3:245. doi: 10.3389/fpubh.2015.00245. PubMed PMID: 26579507; PubMed Central PMCID: PMCPMC4621381.
42. Campbell OM, Calvert C, Testa A, Strehlow M, Benova L, Keyes E, et al. The scale, scope, coverage, and capability of childbirth care. Lancet. 2016. doi: 10.1016/S0140-6736(16)31528-8. PubMed PMID: 27642023.
43. World Health Organization. WHO safe childbirth checklist implementation guide: improving the quality of facility-based delivery for mothers and newborns. Geneva: World Health Organization, 2015.
44. Das J, Hammer J. Quality of Primary Care in Low-Income Countries: Facts and Economics. Annual Review of Economics. 2014;6(1):525-53. doi: 10.1146/annurev-economics-080213-041350.
45. Harvey SA, Blandon YC, McCaw-Binns A, Sandino I, Urbina L, Rodriguez C, et al. Are skilled birth attendants really skilled? A measurement method, some disturbing results and a potential way forward. Bull World Health Organ. 2007;85(10):783-90. PubMed PMID: 18038060; PubMed Central PMCID: PMCPMC2636500.
46. Bohren MA, Vogel JP, Hunter EC, Lutsiv O, Makh SK, Souza JP, et al. The Mistreatment of Women during Childbirth in Health Facilities Globally: A Mixed-Methods Systematic Review. PLoS Med. 2015;12(6):e1001847; discussion e. doi: 10.1371/journal.pmed.1001847. PubMed PMID: 26126110; PubMed Central PMCID: PMCPMC4488322.
47. Das J, Mohpal A. Socioeconomic Status And Quality Of Care In Rural India: New Evidence From Provider And Household Surveys. Health Aff (Millwood). 2016;35(10):1764-73. doi: 10.1377/hlthaff.2016.0558. PubMed PMID: 27702947.

For peer review only

Table 1: Indian Public Health Standard for basic delivery and newborn care, and corresponding question (closest match) in the DLHS-4 facility survey for Primary and Community Health Centers

Indian Public Health Standard	DLHS Question	Data Type
Management of normal deliveries	Can you tell me whether normal delivery services are provided in this facility?	Self-reported
Assisted vaginal deliveries including forceps/vacuum delivery whenever required	Can you tell me whether assisted (forceps delivery/vacuum) delivery services are provided in this facility?	Self-reported
24-hour delivery services both normal and assisted	Whether deliveries are conducted in this facility or no? If yes, whether the deliveries are conducted 24X7?	Self-reported
Manual removal of placenta	NA	
Appropriate & prompt referral for cases needing specialist care	NA	
Management of pregnancy induced hypertension & referral	NA	
Pre-referral management (obstetric first-aid) in obstetric emergencies that need expert assistance	NA	
Proficient in identification and basic first aid treatment for PPH, eclampsia, sepsis and prompt referral	Can you tell me whether administration of parenteral antibiotics are provided in this facility?	Self-reported
	Can you tell me whether administration of parenteral oxytocics are provided in this facility?	Self-reported
	Can you tell me whether management of PPH are provided in this facility?	Self-reported
Minimum 48 hours of stay after delivery	NA	
Managing labor using partograph	Partographs being recorded for the recently delivered women or women in labor at the facility	Self-reported
Facilities for Essential New Born Care and Resuscitation (Newborn Care Corner in LR/OT)	Whether the following essential newborn care services are available? Resuscitation	Self-reported

For peer review only - http://bmjopen.bmj.com/site/about/guidelines.xhtml

Indian Public Health Standard	DLHS Question	Data Type
	Availability of designated newborn baby corner (functional)	Observed
Management of neonatal hypothermia, infection protection, cord care & identification of sick newborn & prompt referral	Whether the following essential newborn care services are available? Thermal protection (warmer/table lamp)	Self-reported
Early initiation of breastfeeding within one hour of birth	NA	
At least one medical officer - MBBS	Availability of human resources (Medical Officer - Regular) - No. in position	Self-reported
Three nurse midwife (staff-nurse)	Availability of human resources (ANM/Female Health Worker - Regular) - No. in position	Self-reported
Health worker female	NA	
Health Assistant (female)	NA	
Training for staff for emergency management to be ensured	Training received by any medical officer during last five years (SBA or Basic Emergency Obstetric Care training)	Self-reported
	Training received for paramedical staff during last five years (SBA training)	Self-reported
Labor table	Physically verify and record availability of labor table with McIntosh sheet	Observed
Suction machine	Physically verify and record availability of suction machine (functional)	Observed
Facility for oxygen administration	NA	
Sterilization equipment (autoclave)	Physically verify and record availability of autoclave/sterilizer (functional)	Observed
24-hour running water	Physically verify and record availability of 24 hour running water supply	Observed
Electricity supply with back-up facility (generator with POL)	Whether the generator supply is connected to the LR	Observed

For peer review only - http://bmjopen.bmj.com/site/about/guidelines.xhtml

Indian Public Health Standard	DLHS Question	Data Type
Attached toilet facilities	Physically verify and record availability of attached toilet in the LR (functional)	Observed
Delivery kits, including those for normal & assisted deliveries	Normal delivery kits available in the facility?	Self-reported
Privacy of woman ensured	Observe and record the condition of the LR - privacy in the LR (satisfactory)	Observed
Radiant warmer, fixed height with trolley	Radiant warmer	Observed
Resuscitation bag and mask with reservoir	Ambu bag with mask (functional)	Observed
Weighing scale	Baby weighing machine of any time (functional)	Observed
Thermometer, clinical, digital	NA	
Light examination	NA	
I/V Cannula 24G, 26G	Suction catheter/cannula (functional)	Observed
Mucus extractor; Pump suction, foot operated	Pedal suction machine/mucus extractor (functional)	Observed
Feeding tube	NA	
Oxygen catheter	NA	
Sterile gloves	Whether following emergency drugs and consumables are available: at least 2 pairs of gloves	Self-reported
Inj. Oxytocin	Whether following emergency drugs and consumables are available: oxytocin injection	Self-reported
Inj. Diasepam	Whether following emergency drugs and consumables are available: diazepam injection	Self-reported
Tab. Nifedepine	Whether following emergency drugs and consumables are available: nifedipine tablet	Self-reported
Inj. Magnesium Sulphate	Whether following emergency drugs and consumables are available: magnesium sulphate injection	Self-reported
Inj. Lignocaine hydrochloride	Whether following emergency drugs and consumables are available: lignocaine hydrochloride injection	Self-reported
Inj. Methyl ergometrine maleate	NA	

For peer review only - http://bmjopen.bmj.com/site/about/guidelines.xhtml

Indian Public Health Standard	DLHS Question	Data Type
I/V Haemaccel	NA	
Sterilized cotton and gauze	Whether following emergency drugs and consumables are available: sterilized cotton and gauze	Self-reported
Syringe	Whether following emergency drugs and consumables are available: sterile syringes and needles (different sizes)	Self-reported
Hub cutter	Hub Cutter (available and functional)	Observed
Oxygen bottles	Whether following emergency drugs and consumables are available: Oxygen cylinder with face mask, wrench & regulator - functional	Self-reported

Indicators were extracted from Indian Public Health Standards (IPHS) for Primary Health Center;
DLHS questions from both Primary and Community Health Center surveys;
NA refers to IPHS indicators that were not included in the DLHS facility survey;
LR: Labor Room.

For peer review only

For peer review only

For peer review only - http://bmjopen.bmj.com/site/about/guidelines.xhtml

Figure 1: Availability of basic delivery and newborn services in primary care facilities in India

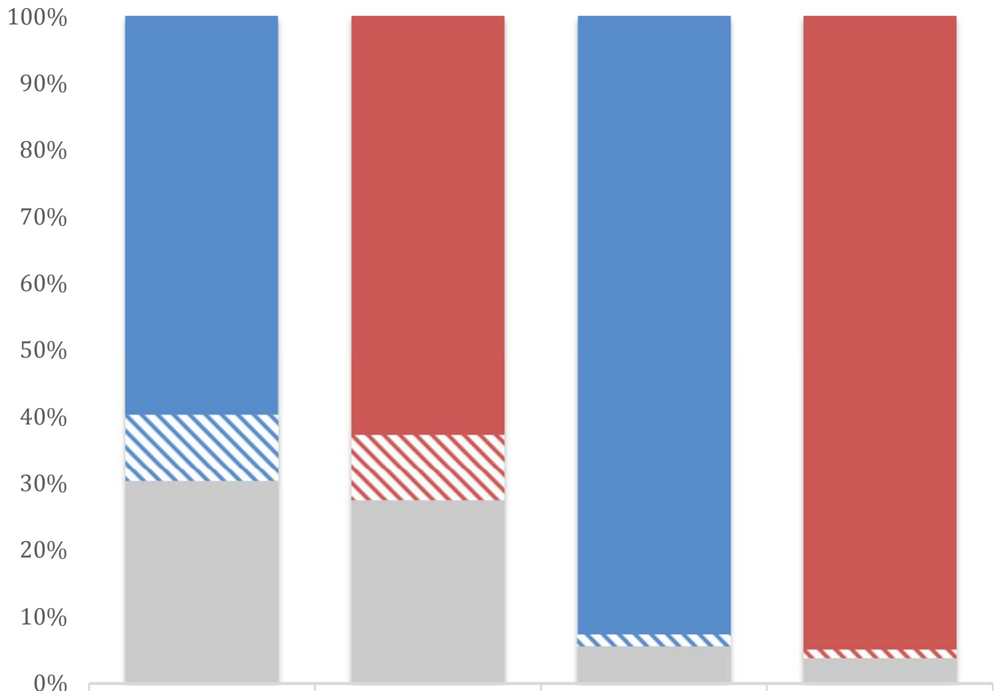

	Primary Health Center		Community Health Center	
	Rural (N=8038)	Urban (N=498)	Rural (N=3861)	Urban (N=943)
24X7	59.7%	62.7%	92.7%	94.9%
Daytime only	10.0%	9.8%	1.8%	1.3%
No service	30.4%	27.5%	5.6%	3.8%

Figure 2: Capacity of Primary Health Centers and Community Health Centers for basic delivery and newborn care

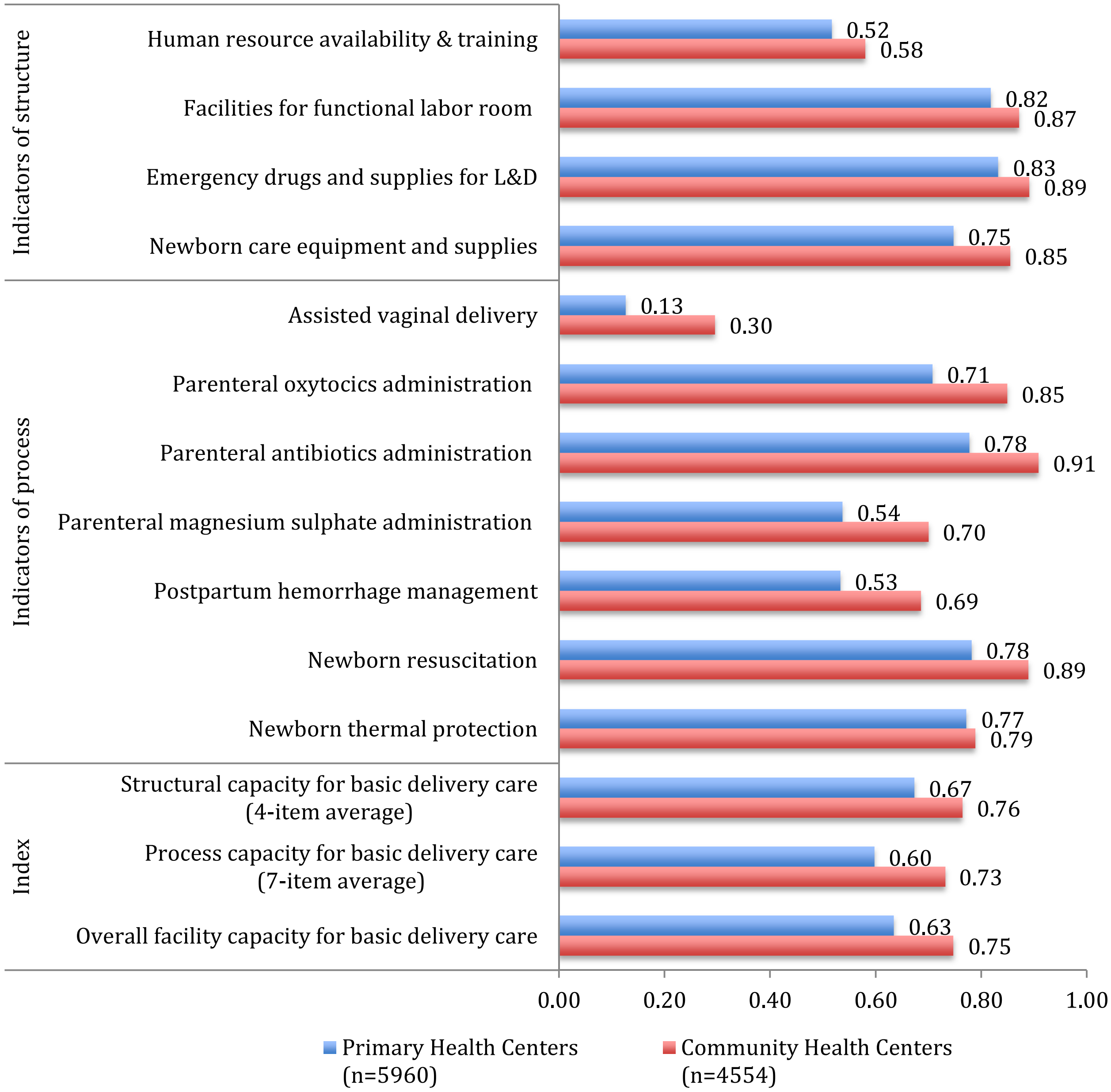

*** Legend:
Overall facility capacity of basic delivery and newborn care calculated as the average of the two preceding summary measures (process and structural capacity). Details on indicators of structure are available in Supplement Figure 1. Differences between PHC and CHC, for all indicators and summary index are statistically significant at $p<0.05$ level (Pearson's chi-square test and Student's t-test).

Figure 3a: Difference in facility capacity for basic delivery and newborn care across rural and urban Primary Health Centers

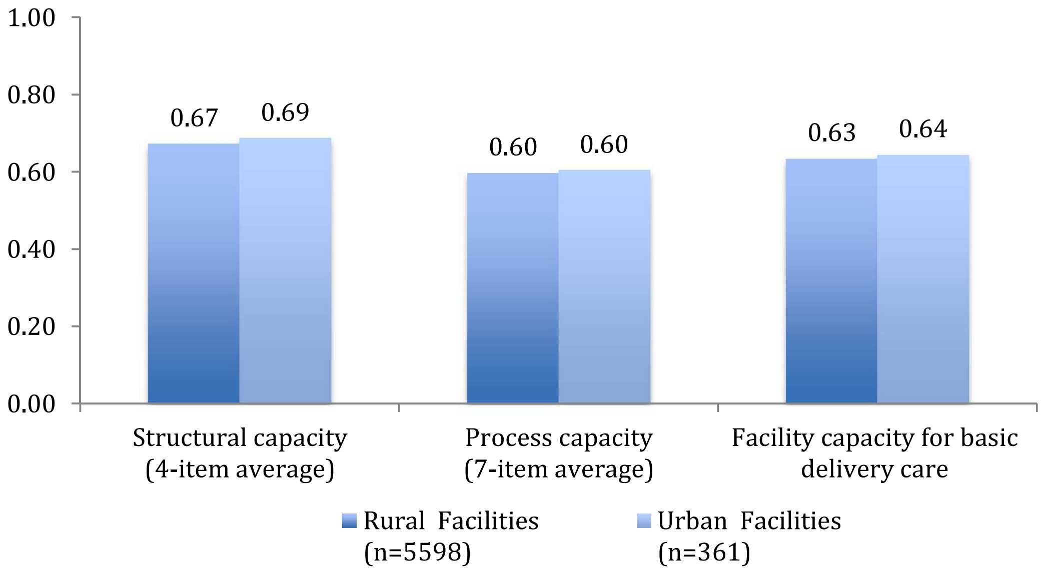

***Legend: Differences between rural and urban facilities for all three summary indices are not statistically significant at p<0.05 level (Student's t-test).

Figure 3b: Difference in facility capacity for basic delivery and newborn care across rural and urban Community Health Centers

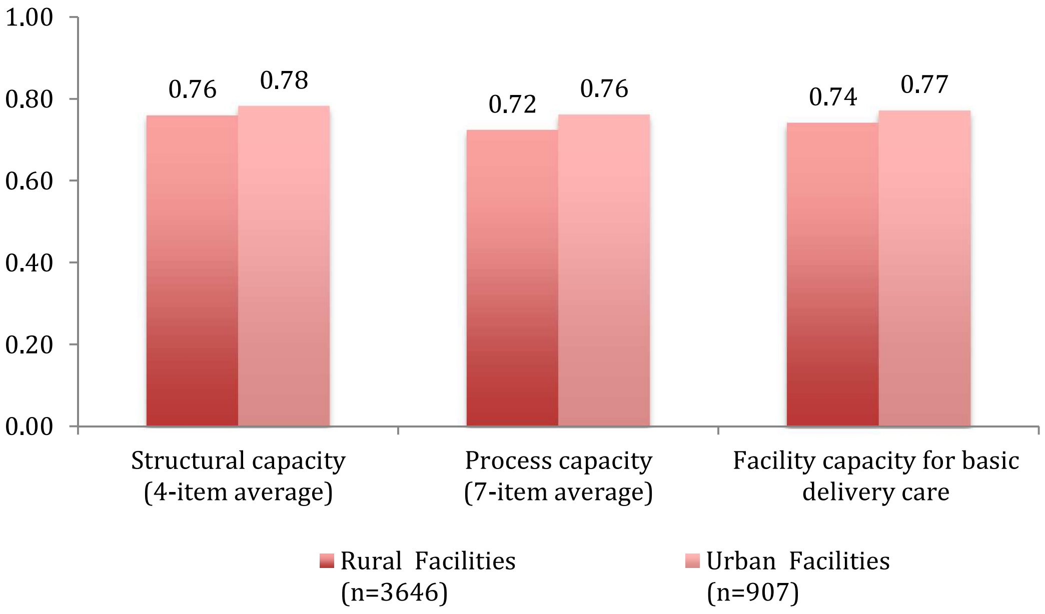

review only

For peer review only - http://bmjopen.bmj.com/site/about/guidelines.xhtml

Figure 4: Empowered Action Group (EAG) status and facility capacity for basic delivery and newborn care

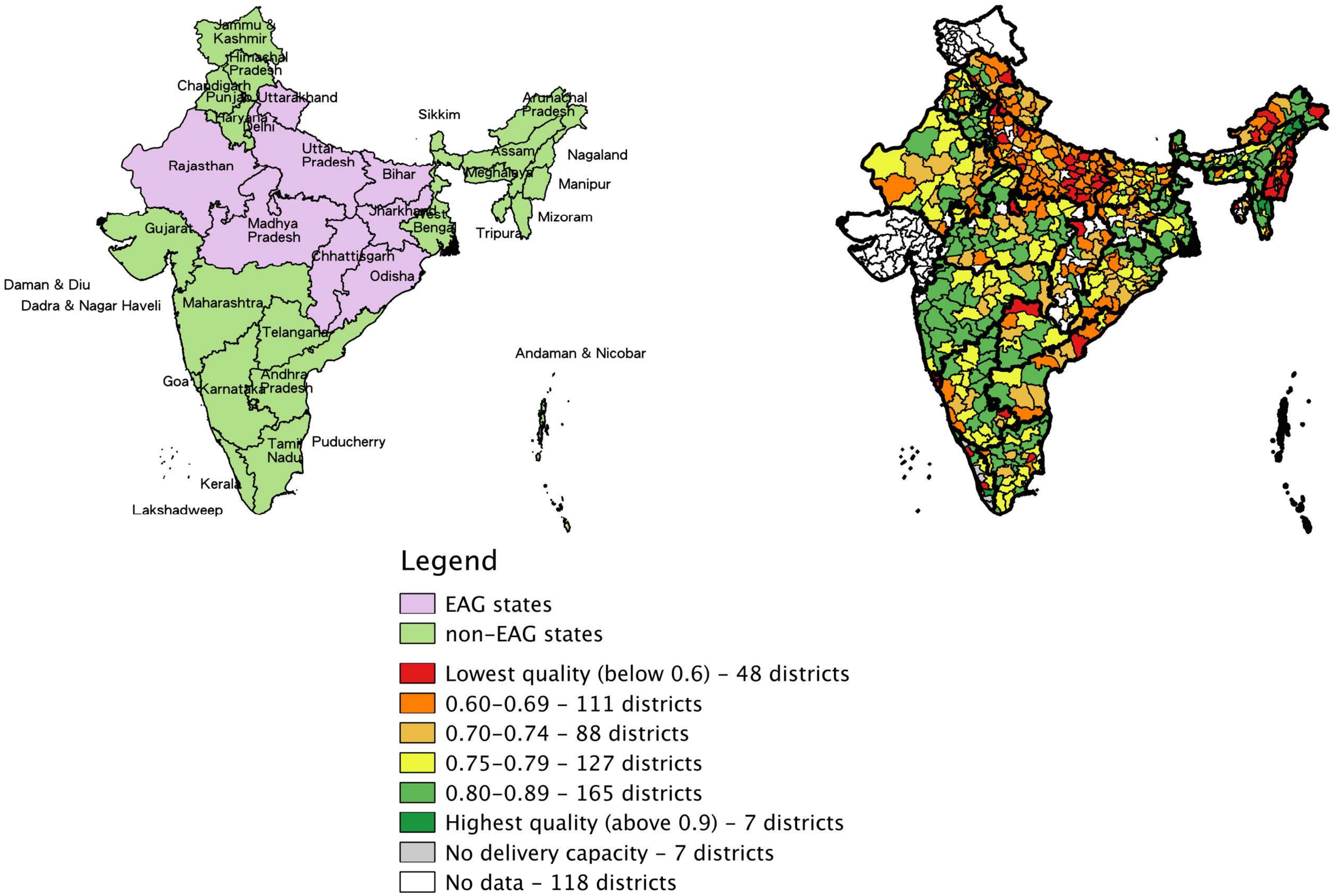

For peer review only

For peer review only - http://bmjopen.bmj.com/site/about/guidelines.xhtml

Supplement Figure 1: Indicators of basic structural capacity for delivery and newborn care in primary facilities

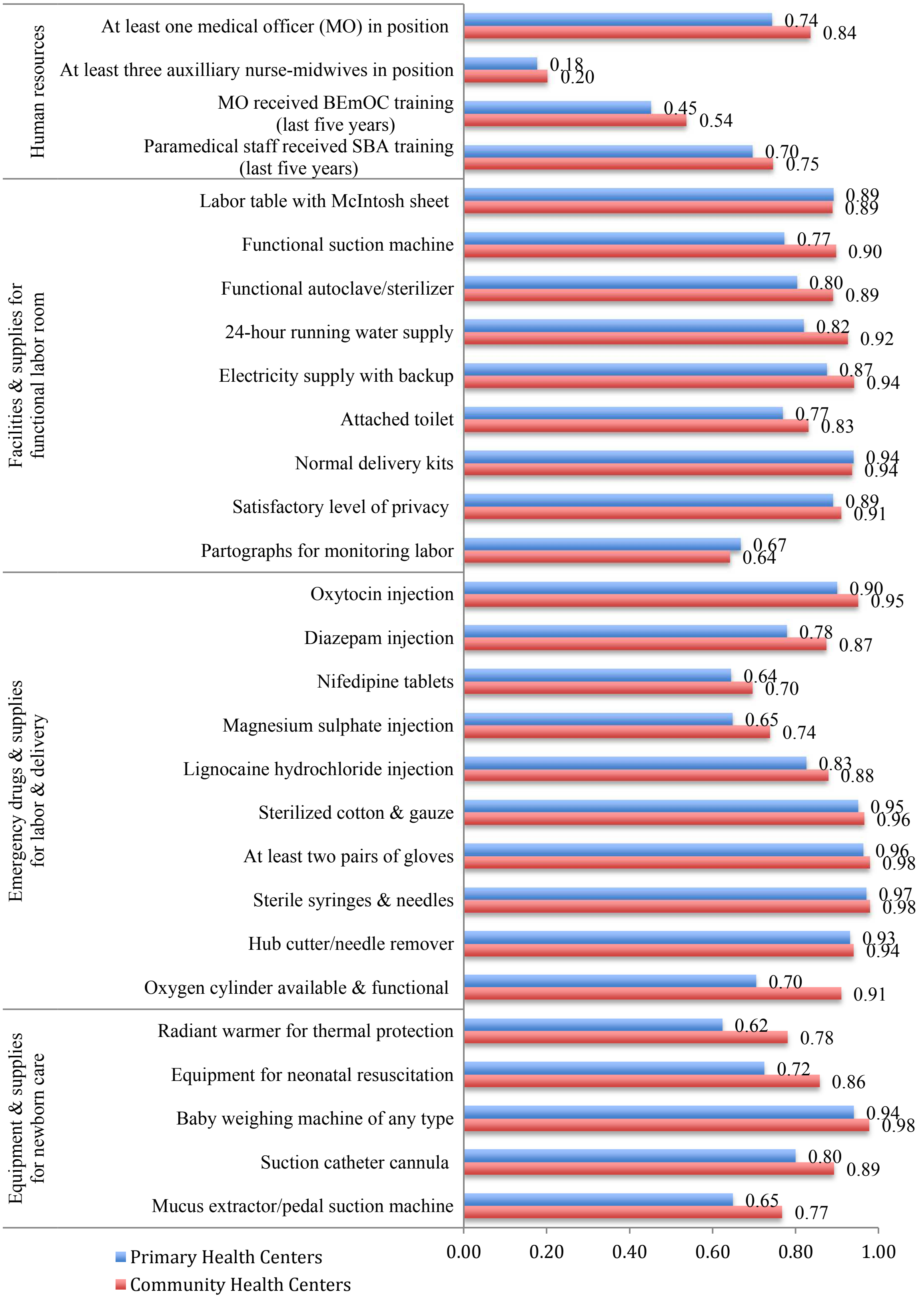

1

Supplemental Figure 2a: Mean facility capacity scores in Empowered Action Group (EAG) states

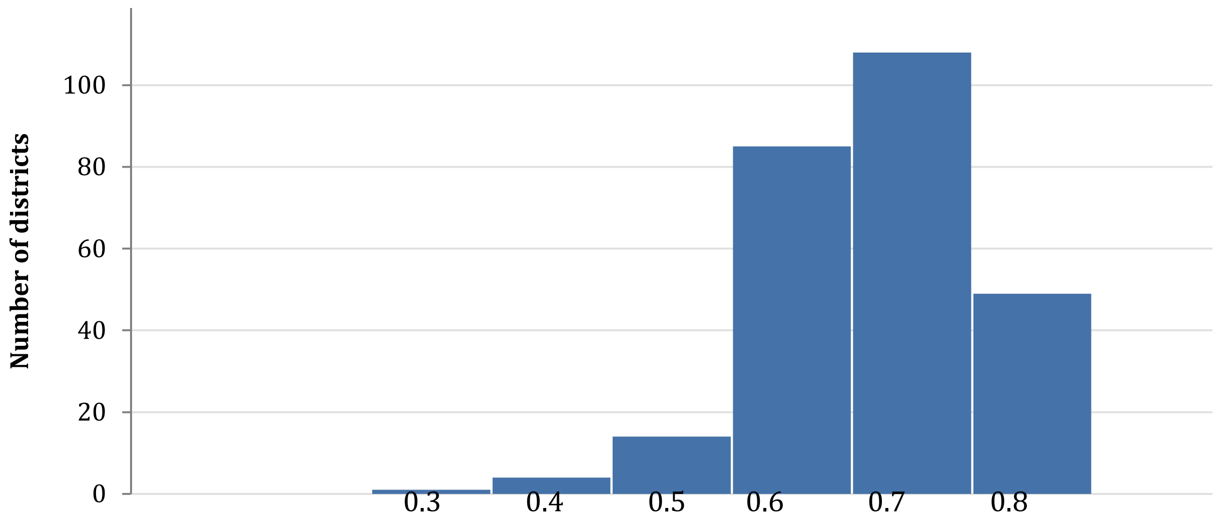

For peer review only - http://bmjopen.bmj.com/site/about/guidelines.xhtml

Supplemental Figure 2b: Mean facility capacity scores in non-EAG states

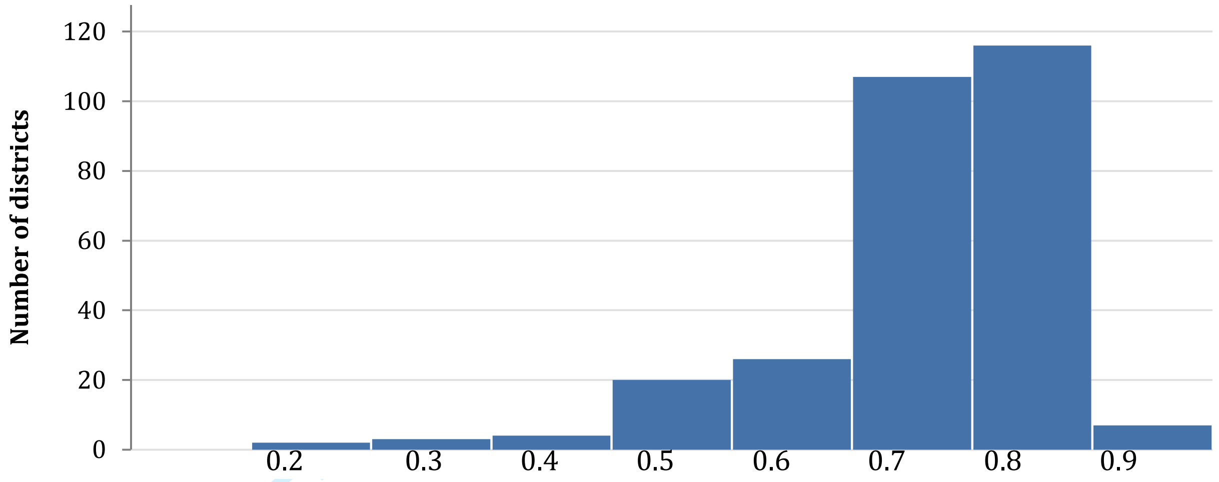

***Legend: Mean facility capacity scores were calculated by summarizing overall capacity at district level, weighting facilities by total deliveries

For peer review only - http://bmjopen.bmj.com/site/about/guidelines.xhtml

BMJ Open

Can India’s primary care facilities deliver? A cross-sectional assessment of the Indian public health system’s capacity for basic delivery and newborn services

Journal:	*BMJ Open*
Manuscript ID	bmjopen-2017-020532.R1
Article Type:	Research
Date Submitted by the Author:	14-Feb-2018
Complete List of Authors:	Sharma, Jigyasa; Harvard T. H. Chan School of Public Health, Global Health and Population<br>Leslie, Hannah; Harvard T. H. Chan School of Public Health, Global Health and Population<br>Regan, Mathilda; Harvard School of Public Health, Global Health and Population<br>Nambiar, Devaki; The George Institute for Public Health, India<br>Kruk, Margaret; Harvard School of Public Health,
**Primary Subject Heading**:	Health services research
Secondary Subject Heading:	Health policy, Global health, Health services research
Keywords:	Health policy < HEALTH SERVICES ADMINISTRATION & MANAGEMENT, Quality in health care < HEALTH SERVICES ADMINISTRATION & MANAGEMENT, Health Systems, Maternal Health, Newborn Health

SCHOLARONE™
Manuscripts

For peer review only

Can India's primary care facilities deliver? A cross-sectional assessment of the Indian public health system's capacity for basic delivery and newborn services

Jigyasa Sharma, MSc[1], Hannah H Leslie, PhD[1], Mathilda Regan, MPH[1], Devaki Nambiar, PhD[2], Margaret E Kruk, MD[1]

[1]Department of Global Health and Population, Harvard T.H. Chan School of Public Health, USA
[2] The George Institute for Public Health, India

Corresponding Author:
Jigyasa Sharma
Department of Global Health and Population
Harvard T.H. Chan School of Public Health
655 Huntington Avenue
Boston, MA 02115
jigyasa.sharma@mail.harvard.edu

For peer review only

Abstract

Objectives

Using nationally representative District Level Household and Facility Survey, (1) assess input and process capacity for basic delivery and newborn (intrapartum care hereafter) care in the Indian public health system and (2) describe differences in facility capacity between rural and urban areas and across states.

Setting

Indian public primary care facilities (Primary Health Centers [PHCs] and Community Health Center [CHCs]).

Methods

We developed a summative index of 33 structural and process capacity items matching the Indian Public Health Standards for PHCs as a metric of minimum facility capacity for intrapartum care. We assessed differences in performance on this index across facility type and location.

Results

Our study sample consisted of 8536 PHCs and a census of 4810 CHC facilities. About 30% of PHCs and 5% of CHCs reported not offering any intrapartum care. Amongst those offering services, volumes were low: median monthly delivery volume was 8 (IQR=13) in PHCs and 41 (IQR=73) in CHCs. Both PHCs and CHCs failed to meet the national standards for basic intrapartum care capacity. Mean facility capacity was low in PHCs in both urban (0.64) and rural (0.63) areas, while in CHCs, capacity was slightly higher in urban areas (0.77 vs. 0.74). Gaps were most striking in availability of skilled human resources and emergency obstetric services. Poor capacity facilities were more

For peer review only

concentrated in the more impoverished states, with 37% of districts from these states receiving scores in the lowest third of the facility capacity index (<0.70), compared to 21% of districts otherwise.

Conclusion

Basic intrapartum care capacity in Indian public primary care facilities is weak in both rural and urban areas, especially lacking in the poorest states with worst health outcomes. Improving maternal and newborn health outcomes will require focused attention to quality measurement, accountability mechanisms, and quality improvement. Policies to address deficits in skilled providers and emergency service availability are urgently required.

For peer review only

Strengths and limitations of this study

1. Uses a nationally representative survey to assess primary public health facilities' adherence to nationally set minimum recommended standards for basic delivery and newborn (intrapartum) care.
2. The index used to assess input and process capacity is based on national guidelines, and therefore represents a contextually appropriate measure of basic service readiness.
3. However, the index lacked information on important technical and interpersonal care processes, such as provider competence, owing to data limitation.
4. Similarly, limited data constrained our ability to assess quality of referral care, and quality of care in private primary care facilities, which may perform differently on our basic intrapartum care capacity index.

Ethical approval:

The Harvard University Human Research Protection Program approved this analysis as exempt from human subjects review.

Funding statement

This research received no specific grant from any funding agency in the public, commercial or not-for-profit sectors.

Competing interests statement

For peer review only - http://bmjopen.bmj.com/site/about/guidelines.xhtml

We have read and understood BMJ policy on declaration of interests and declare that we have no competing interests.

Author contributions

JS, HHL and MEK conceived the research question. JS and HHL developed the methods, and JS, HHL and MR conducted the data analysis. JS, HHL, MR, DN and MEK interpreted the results, and JS wrote the first draft of the manuscript. JS, HHL, MR, DN and MEK edited and revised the manuscript and approved the final draft for submission

Data sharing statement

The data are publicly available through the International Institute of Population Sciences, India. Website: http://www.iipsindia.ac.in

For peer review only

Introduction

The adoption of Sustainable Development Goals (SDGs) has reaffirmed the reduction of preventable maternal and newborn deaths as global health priorities. Achieving the targeted reduction of the global maternal mortality ratio (MMR) to less than 70 per 100,000 live births and neonatal mortality to 12 per 1,000 live births [1] will require near universal coverage of institutional deliveries and timely detection and management of birth complications, as most maternal and newborn deaths occur at birth or within 24 hours of birth [2]. An emerging body of literature suggests that getting women to facilities alone will not suffice: what happens when a woman reaches a facility – including her right to quality antenatal, intrapartum, and postpartum care – matters [3, 4], especially given the global mandate of universal health coverage [5, 6].

In India, increased coverage of facility-based births has not successfully translated to desired improvement in health outcomes for mothers and newborns. Under India's National Rural Health Mission (NRHM, now called the National Health Mission or NHM), a variety of interventions were introduced through architectural improvements in the fund flow and design of services: increased number of maternal care facilities, particularly primary health centers (PHCs) and community health centers (CHCs); a strengthened supply chain for essential medicines, equipment and supplies; and Janani Surakshya Yojana (JSY), a financial-incentive program to increase institutional deliveries [7]. Following the launch of NHRM in 2005, institutional deliveries in rural areas have more than doubled [8], and a declining trend in maternal and newborn mortality has been noted, although strong causal evidence linking NRHM efforts to improved health

For peer review only - http://bmjopen.bmj.com/site/about/guidelines.xhtml

outcomes for mothers and newborns is lacking [9-11]. Annual decline in neonatal mortality between 2005 and 2015 was faster than in the preceding years; however, the rate of decline is not sufficient to meet the 2030 SDG targets [12]. Inadequate quality of care, including insufficient facility readiness, and low provider skill and clinical management capacity, as evidence from low and middle income (LMIC) countries indicates, may explain why increased utilization alone may not have resulted in the desired reduction in adverse intrapartum outcomes [13, 14]. Moreover, quality of care itself also affects utilization. Evidence from India indicates that the availability of a labor room and adequacy of essential equipment and laboratory services for childbirth at public health facilities have a significant effect on service uptake [15, 16]. Describing and improving quality of intrapartum care is relevant to improving service uptake in India, where maternal and newborn services are underutilized despite the availability of primary healthcare in public health facilities free of charge.

Public health facilities are a significant provider of care in India, especially for rural and vulnerable population segments. About 80.1% of all deliveries in rural India are facility-based, of which about 70% are in public facilities [17]. For urban areas, 89.5% of births are institutional, 47.4% of which are in public facilities [17]. In the majority of India's states, more facility-based deliveries happen at government health institutions than in private facilities [18, 19]. Additionally, quality of care in the public sector affects the poorest segment of the population the most, as the poorest wealth quintile class is more heavily reliant on public health facilities than the richest, in both rural (58% vs. 29%) and urban areas (48% vs. 19%) [17, 20].

For peer review only - http://bmjopen.bmj.com/site/about/guidelines.xhtml

India has made several efforts to strengthen its public health service provision. Initial policy emphasis was on rural areas given the preponderance of the Indian population living there and the assumption that population access to quality health services was relatively better in urban areas. However, mounting evidence that the health of the urban poor lagged that of the rich, and sometimes even rural populations, led to the formal launch of the National Urban Health Mission in 2013 [21]. The unified National Health Mission is now charged with oversight for public health care in urban and rural areas [22]. The implementation of programs and policies, however, has varied sub-nationally. Under the NRHM, substantial funding and technical support has been provided to states with relatively weaker public health indicators and health system infrastructure, including the Empowered Action Group (EAG) states (Bihar, Jharkhand, Chhattisgarh, Madhya Pradesh, Uttar Pradesh, Uttaranchal, Orissa and Rajasthan) [23]. About 46% of India's population resides in the EAG states, and this region lags behind the rest of the Indian states on socio-economic, demographic and health indicators [23].

To boost the performance of public health facilities, in 2007 the government introduced the Indian Public Health Standards (IPHS), a set of recommendatory standards to be used as a reference for public health infrastructure planning and as a benchmark for assessing the functional status of health facilities [24]. Although widely used in health system planning and management, the use of these standards in health system research has been limited in number and geographic scope to date. Rural Health Statistics from 2015 note that only 21% of PHCs and 26% of CHCs were functioning in concordance with IPHS

For peer review only - http://bmjopen.bmj.com/site/about/guidelines.xhtml

standards, although a service-specific breakdown was not provided [25]. A few studies have utilized IPHS as a reference, noting deficiencies in service availability, human resource and infrastructure [26-29]. With the exception of one [29], all these studies surveyed less than a hundred facilities.

The aim of this work is to understand the capacity of the Indian public health system to provide quality intrapartum care. This is an important element of understanding the effects of maternal and newborn health policies to date and setting priorities for health system strengthening going forward. In this paper, using the updated IPHS (2012) as the minimum standard for basic facility capacity for intrapartum care, we (1) assess the performance of PHCs and CHCs in India against this standard; and (2) describe differences in intrapartum care capacity between urban and rural areas, and across states.

Methods

Study sample

We drew data from the fourth cycle of the District Level Household and Facility Survey (DLHS) conducted by the International Institute of Population Sciences in India. We used data from the facility modules of the DLHS for PHCs and CHCs to capture the primary level of the public health system designated to provide intrapartum care services. Typically, PHCs serve a population of 20,000 to 30,000 [30], and CHCs, which are intended to be referral centers for four PHCs, cater to approximately a population of 80,000 to 120,000 [30]. Within the tiers of the Indian public health system, CHCs are referral centers for PHCs; yet they are frequently used for first-line intrapartum care. In

For peer review only

For peer review only - http://bmjopen.bmj.com/site/about/guidelines.xhtml

this paper, in keeping with international guidelines, both are referred to as primary care facilities [31].

The most recent survey, DLHS-4, was conducted in 2012-2014 and contains a sample of PHCs designed to be representative at district level and a census of CHCs. We also obtained the most recent district and state boundaries from the database of global administrative areas (GADM.org) and matched DLHS data to the appropriate area using state and district name. We were unable to match three districts, likely due to district boundary changes between 2013 and 2016.

Basic intrapartum care capacity index

Availability of essential physical and human resources and services is foundational to the provision of high-quality care [32]. The study objective was to evaluate basic intrapartum care capacity that should be present in all childbirth facilities. As described in the WHO quality of care framework for maternal and newborn health [32], which builds on Donebedian's framework of healthcare quality [33], structural capacity elements, such as availability of drugs and supplies, refer to necessary, but not sufficient, human and physical resource conditions for care delivery. Process capacity elements refer to facility's ability to offer evidence-based practices for routine care and management of complications, such as assisted vaginal delivery or administration of parenteral antibiotics.

We used the IPHS for PHCs to define intrapartum care capacity in this context; CHCs are assumed to meet at least the standards of the lower-tier PHCs [30]. The facility capacity

For peer review only - http://bmjopen.bmj.com/site/about/guidelines.xhtml

index comprises structure and process capacity elements required for both routine and basic emergency obstetric care.

We identified 48 elements of the IPHS pertaining to intrapartum care. Of these, we found at least some information on 33 in the DLHS facility audit, of which 26 were a complete match. Facility capacity to offer services such as manual removal of placenta, appropriate pre-referral management of and referral for obstetric emergencies, and availability of select drugs and equipment, identified as the basic requirements for all PHCs and higher-level facilities, were not included in the facility audit. See Table 1 for the full list of IPHS requirements along with corresponding item from DLHS survey.

Our data comprised both observed and self-reported measures of structural inputs to care and self-reported process capacity indicators. Structural inputs fell into four sub-domains: skilled provider availability (average of 4 indicators), facilities for a functional labor room (average of 9 indicators), emergency drugs and supplies for labor (average of 10 indicators), and delivery and newborn care equipment and supplies (average of 5 indicators). Process capacity indicators included reports from survey respondent (facility managers or medical officers in charge) on availability of assisted vaginal deliveries; administration of parenteral oxytocins, antibiotics, magnesium sulfate; postpartum hemorrhage management; newborn resuscitation and thermal protection services. All process capacity indicators were binary measures. The items were grouped following the IPHS categorization. See Supplemental Figure 1 for details on all items.

For peer review only - http://bmjopen.bmj.com/site/about/guidelines.xhtml

For both structure (4 sub-domains) and process (7 items) capacity indices, sub-domains or indicators were averaged to provide a facility summary score from 0 to 1, with missing values excluded. Missing values were minimal (less than 3% of facilities) for all indicators except for availability of newborn resuscitation (11% of PHCs missing) and electricity supply with backup (23.6% of PHC and 9.6% of CHC missing). We created an index of facility capacity for basic intrapartum care as an average of the structure and process capacity indices. We followed current practice and weighted each indicator equally within a sub-domain or index given the lack of guidance in the IPHS on weights [34, 35].

Covariates

We identified covariates that may be associated with facility capacity. We followed DLHS classification of facilities as either a PHC or CHC, which is based on delivery volume and their capacity to perform comprehensive obstetric emergency management. We categorized facilities by rural versus urban location as reported in the survey. We also calculated annual delivery volume based on facility report, excluding missing values. We calculated capacity for quality intrapartum care within districts by weighting each facility by delivery volume. For weighting, we assigned facilities reporting childbirth capacity but missing delivery volume data or reporting no deliveries a delivery volume of 1.

Statistical analysis

In this analysis, to describe primary level public health facilities' capacity to provide basic intrapartum care, we first calculated availability of basic childbirth care services at

For peer review only - http://bmjopen.bmj.com/site/about/guidelines.xhtml

the facility level: facilities not offering any services, facilities offering round-the-clock services, and those providing daytime services only. Some PHCs and CHCs may have curtailed services possibly due to low volume (resulting from proximity to a higher-level facility), inadequate infrastructure or human resources. As our data do not support classification of these facilities based on the reason for service unavailability (which may have been justified or not, given individual circumstances), we restricted subsequent analysis to facilities reporting provision of either round-the-clock or daytime only childbirth services. In this sample, we assessed each facility's adherence to national standard for care provision, stratified by facility type (PHC or CHC).

We described variation in facility-level capacity across rural and urban settings. We employed Pearson's chi-square test for binomial and Student's T-test for continuous indicators to assess the statistical significance ($p<0.05$) of difference in capacity between PHCs and CHCs and between rural and urban PHCs. To visualize differences in intrapartum care capacity across India, we also calculated averages of overall facility intrapartum care capacity at the district level, weighting facilities by total deliveries. For mapping, we divided the overall facility capacity index scores into sixths, designating the lowest third of scores as "poor capacity."

Statistical analysis was done using Stata 14.1 (StataCorp, Texas) and mapping using QGIS Version 2.12 (Free Software Foundation, Massachusetts).

Ethical approval

For peer review only - http://bmjopen.bmj.com/site/about/guidelines.xhtml

The Harvard University Human Research Protection Program approved this analysis as exempt from human subjects review.

Results

The 2012-2013 DLHS-4 facility survey was conducted across India; however, data are publicly available only for 30 out of 36 states and union territories (excluding the states of Gujarat, and Jammu and Kashmir, and the territories Dadar and Nagar Haveli, Daman Diu, the National Capital Territory of Delhi, and Lakshadweep). Our study sample comprised a total of 8536 PHCs and 4810 CHCs. Although all PHCs and CHCs are expected to provide childbirth care, about 30.2% (N=2557) of PHCs and 5.2% (N=251) of CHCs reported not providing any childbirth services, either round-the-clock or daytime-only. Only 59.7% (N=4798) of rural and 62.7% (N=312) of urban PHCs and only 92.7% (N=3578) of rural and 94.9% (N=895) of urban CHCs offered 24-hour intrapartum care. As Figure 1 indicates, availability of services was similar for both levels of facility across urban and rural settings.

Within facilities offering any delivery services (N= 5959 PHC, N= 4553 CHC), median monthly delivery volume was 8 (IQR=13) in PHCs and 41 (IQR=73) in CHCs. 73.4% of the PHCs self-identified as government-designated 24x7 delivery facilities; 24x7 services were offered in 86% and 98% of all included PHCs and CHCs, respectively. A designated area for newborn care was present only in 67% and 80% of included PHCs and CHCs, respectively.

For peer review only - http://bmjopen.bmj.com/site/about/guidelines.xhtml

Overall capacity for basic intrapartum care was lower than the basic IPHS standard in both PHCs (mean 0.63, standard deviation [SD] 0.23) and CHCs (mean 0.75, SD 0.17) included in this analysis. The structural capacity index for basic intrapartum care was slightly better than the process capacity index for both PHCs (mean 0.67, SD 0.19 vs. 0.60, SD 0.29) and CHCs (mean 0. 76, SD 0.14 vs. 0.73, SD 0.22). As shown in Figure 2, PHCs lagged behind CHCs on all indicators; the difference between PHCs and CHCs was statistically significant for all indicators and the summary index. Although CHCs, as the referral centers for PHCs, are expected to offer more comprehensive care, they frequently lacked basic infrastructure required even at PHCs. Human resource availability and training in both CHCs (mean 0.58, SD 0.23) and PHCs (mean 0.52, SD 0.26) were comparably low. Particularly large gaps were seen in process capacity indicators such as provision of assisted vaginal deliveries (13% of PHCs versus 30% of CHCs), administration of parenteral magnesium sulfate (54% versus 70%), and management of postpartum hemorrhage (53% of PHCs versus 69% of CHCs).

Figures 3 and 4 display the differences in structural and process capacity indices, and in overall capacity of basic intrapartum care across rural and urban settings, for PHCs and CHCs, respectively. Facility capacity was low across the board, with small differences across geographical settings. Scores for structural, process, and overall capacity summary indices were comparable for PHCs, with no significant rural-urban differences. Rural-urban differences in CHCs were slightly more pronounced, with urban CHCs outperforming rural CHCs by 0.02 to 0.04 in structural, process, and overall facility capacity indices.

For peer review only - http://bmjopen.bmj.com/site/about/guidelines.xhtml

As shown in Figure 5, the capacity of facilities that offered intrapartum care varied across the country, with districts with the poorest facility capacity concentrated in the northern part of the country. Seven districts had no facilities offering childbirth services. We had no data for 118 districts, including the districts in regions for which survey data were not publicly available. In both EAG and non-EAG states, there was significant variation in facility capacity (Supplemental Figures 2 and 3). There was a disproportionate number of poor capacity facilities in EAG states, with 37% of districts scoring in the lowest third of the facility capacity index (less than 0.70) compared to 21% of non-EAG states. Among the nine EAG states, Uttar Pradesh had the greatest concentration of poor capacity facilities, with 84% percent of districts receiving scores in the lowest third.

Discussion

Using a national health facility survey, we found that many Indian public primary care facilities fail to meet the nationally set minimum recommended standards for basic intrapartum care. Despite a concerted effort to increase accessibility, about 30% of PHCs and 5% of CHCs did not offer any childbirth services; round-the-clock intrapartum care services were available only in 60% of PHCs and 94% of CHCs. Both PHCs and CHCs offering these services had critical deficiencies in routine and emergency care practices, infrastructure and staffing, fulfilling an average of only 63% and 75% of an index of basic intrapartum care capacity, respectively. These findings are consistent with studies documenting weak emergency care capacity, infrastructure and staffing in low-income countries, particularly in lower level facilities with low delivery volume [34, 36]. Most

For peer review only - http://bmjopen.bmj.com/site/about/guidelines.xhtml

CHCs, which are meant to serve as referral centers to manage more complicated cases [23], failed to meet the threshold set for lower level facilities.

Structural capacity across the study facilities was marginally better than process capacity, with large gaps in provision of signal functions of emergency obstetric care [37]. For example, assisted vaginal deliveries were offered only in about one-tenth of PHCs and one-third of CHCs. Almost one-half of PHCs and one-third of CHCs reported not providing services to manage postpartum hemorrhage, a leading cause of maternal mortality in India. We also noted that availability of medicine and supplies, such as the partograph or uterotonics, was worse than that of equipment and infrastructure, such as scales or labor tables, underscoring the need to strengthen the supply chain for essential medicine and supplies. This is a challenge that NRHM was designed to address, and one that has been resistant to change [16].

Similarly concerning are the low scores on human resource availability and training across facilities. Although addressing the shortage of skilled providers in rural PHCs and CHCs has also been one of the agendas of the NRHM, critical deficiencies clearly persist, as has been documented elsewhere [38]. The gaps observed are especially problematic because our indicators reflect a minimum level of human resource preparedness (for example, skilled birth attendance training for auxiliary nurse-midwives or emergency obstetric care training for medical officers). They do not include key dimensions of provider availability and competence, which the World Health Organization identifies as integral to good quality of care during facility-based deliveries [39]. For example,

For peer review only

For peer review only - http://bmjopen.bmj.com/site/about/guidelines.xhtml

accounting for absenteeism, a common problem in public facilities in India, would probably lower our estimate of actual human resource availability significantly [38].

Facility capacity for basic intrapartum care in public facilities was similar in rural and urban areas. Our findings underline the need to improve facility capacity in both urban and rural areas, which the unified National Health Mission may be well situated to tackle. Given the fact that the public facilities assessed here are the main source of obstetric care for poor populations in both rural and urban settings, [40, 41] the deficiencies we found are likely to disproportionately affect the most vulnerable women. Meeting the commitment to equitable care inherent in the National Health Mission requires redoubled attention on care quality in the public health system.

Despite additional funding and considerable technical support for improving maternal and newborn health outcomes in the EAG states, facility capacity for intrapartum care remains weaker than in non-EAG states. We found that facilities with poor capacity were especially common in Uttar Pradesh and Bihar, states with the two highest maternal and newborn mortality rates in the country. Roughly comprising a third of India's population, these two states are critical in India's progress towards meeting national and international maternal and newborn mortality reduction targets.

Our findings indicate very low monthly delivery volume in PHCs. Bypassing of care at lower-level facilities may be driven by a myriad of reasons [42], including, but not limited to, women's preference for higher quality care when it is available, as seen in

For peer review only

Tanzania [43]. Regardless of the reason, underutilization of PHCs represents inefficiency in the Indian health system, considering the amount of resources spent at this level of healthcare [44]. This merits attention for effective planning and resource allocation as healthcare delivery structure being developed for urban India under the National Urban Health Mission follows the same model as in rural areas [22].

A demand for quality care, regardless of the level of facility, has been noted in India. Preference for higher-level facilities with better quality is observed in the state of Kerala in India [17], whereas in Tamil Nadu, women prefer to delivery in PHCs with qualified staff providing respectful care [45]. Most high-income and many middle-income countries encourage delivery in hospitals where surgical and newborn care services are available to all women [46]. Organizing health system services around the capacity to deliver high-quality care may thus respond to women's observed preferences and improve health system performance. The breadth of quality deficits in CHCs and especially PHCs, which are under-resourced despite concerted efforts and investment of the NHM, is concerning. Rather than attempting to bring many thousand facilities up to standards, regionalization of obstetric care and quality improvement in high-volume facilities may offer a more efficient resource allocation strategy for the general population. Building on the success of NRHM in using supply- and demand-side strategies to reduce barriers to facility-based delivery [47], India may be particularly well positioned to assess the feasibility of regionalizing care. Assessment of regionalization should include checks against over-medicalization of childbirth care, which has been seen in some states [48]. That said, it is essential to strengthen lower-level facilities and

For peer review only

improve referral linkages with higher-level facilities in areas with highly remote, rural and/or vulnerable populations, to ensure continued access to services for all, especially considering evidence from India indicating preference for PHCs when they are adequate [45, 49].

Our study has several limitations. First, it would have been ideal to examine the quality of clinical care provided to women in public health facilities such as the use of basic emergency care procedures for mothers and newborns (signal functions) and adherence to global guidelines for routine deliveries [35, 50]. Our index was constructed from indicators of basic equipment and service availability, measuring facility level inputs to care and basic service readiness, but it lacked information on important technical and interpersonal care processes, such as provider competence. Previous studies have noted the importance of provider knowledge and effort in determining the quality of actual clinical encounters [51] as well as substantial variation in the skill and competence of skilled birth attendants [52]. Likewise, although disrespectful care during childbirth is common [53] and respectful care is identified as WHO as a key requirement for improving quality of intrapartum care [39], we could not assess interpersonal care aspects in our index. Critically, data constrained our ability to assess private facilities, which are also relied on for intrapartum care and may have different adherence to IPHS standards. We were also not able to assess quality of referral care or the outcomes of care. Thus, our measure should be seen as a starting point in measuring care quality in India; process and outcome quality may well be worse than the largely input-based measures examined here.

For peer review only

Finally, these findings may not be representative of the six states and union territories without the survey data.

Our study findings add to the growing body of literature documenting gaps in provision of quality intrapartum care in LMICs as well as inadequate measurement to assess health system quality. For fifteen out of forty-eight intrapartum care items on the IPHS, we did not find any corresponding survey question in the DLHS facility audit. Harmonizing data collection with national standards is a critical first step towards addressing information gaps. Likewise, expanding data collection to capture the nature and content of care being provided is important for understanding quality of care [54]. Quality of care is complex and multifaceted. While the inputs to care measured here are a critical foundation for high-quality care, they are a poor proxy of care as delivered [55]. Even measures of process quality may be insufficient to predict quality-sensitive outcomes such as morbidity and mortality [10, 56, 57], particularly for childbirth where averting severe outcomes requires timely recognition of complications by qualified personnel and functional referral systems to ensure a rapid response [10, 57].

Future assessments should better capture the context of care and whether women's rights to quality care are being met, with a focus on clinical effectiveness of care, patient experience, respectful care, and outcomes [32]. A variety of methods are available for this, including patient and provider interviews, direct observations, and clinical vignettes; assessment of the private sector is essential to gaining a full understanding of health system performance [51]. Strengthening the publicly available health information system

For peer review only - http://bmjopen.bmj.com/site/about/guidelines.xhtml

in India to collect reliable, frequent and timely data on facility quality is key for effective program implementation and for meeting national and global targets such as those of the Ending Preventable Maternal Mortality, the Every Newborn Action Plan, and the SDGs. It is also essential to link quality measures to health outcomes and demographic characteristics of the population to assess the determinants of quality and its distribution across population subgroups. Health systems research in India needs to be expanded to address the large knowledge gaps in health system quality [58].

Conclusion

Primary care facilities in India are not well prepared to provide high quality obstetric and newborn care, and facility capacity is worst in states with the worst health outcomes. Over the past decade, India's health system has operated in an extremely resource-constrained environment: from 2004-2014, government health expenditure has remained approximately around 1% of country's GDP [20]. The Indian government will need to increase investment in the health system, in providers, and in research to harness the full benefit of its public health infrastructure. Research on regionalization is a priority as this may offer an innovative approach to ensuring quality services for mothers and newborns. The impact of regionalization strategies on facility overcrowding, performance incentive structures for frontline workers, as well as equity in service access should be important considerations of such research. Improving quality of care and strengthening public health infrastructure is integral to India's path to universal health coverage: without an explicit focus on quality, a push towards universal coverage is unlikely to lead to better health for mothers and newborns.

For peer review only - http://bmjopen.bmj.com/site/about/guidelines.xhtml

References

1. UN Development Program. Sustainable Development Goals. Geneva, United Nations: 2015.
2. The Partnership for Maternal Newborn & Child Health. A Global Review of the Key Interventions Related to Reproductive, Maternal, Newborn and Child Health (RMNCH). Geneva, Switzerland: PMNCH: 2011.
3. Dickson KE, Kinney MV, Moxon SG, Ashton J, Zaka N, Simen-Kapeu A, et al. Scaling up quality care for mothers and newborns around the time of birth: an overview of methods and analyses of intervention-specific bottlenecks and solutions. BMC pregnancy and childbirth. 2015;15 Suppl 2:S1. Epub 2015/09/24. doi: 10.1186/1471-2393-15-s2-s1. PubMed PMID: 26390820; PubMed Central PMCID: PMCPmc4578819.
4. Bhutta ZA, Das JK, Bahl R, Lawn JE, Salam RA, Paul VK, et al. Can available interventions end preventable deaths in mothers, newborn babies, and stillbirths, and at what cost? Lancet. 2014;384(9940):347-70. doi: 10.1016/S0140-6736(14)60792-3. PubMed PMID: 24853604.
5. Kruk ME, Larson E, Twum-Danso NA. Time for a quality revolution in global health. The Lancet Global health. 2016;4(9):e594-6. Epub 2016/08/20. doi: 10.1016/s2214-109x(16)30131-0. PubMed PMID: 27539798.
6. Sobel HL, Huntington D, Temmerman M. Quality at the centre of universal health coverage. Health Policy Plan. 2016;31(4):547-9. Epub 2015/10/01. doi: 10.1093/heapol/czv095. PubMed PMID: 26420642.
7. Nandan D. National rural health mission: turning into reality. Indian J Community Med. 2010;35(4):453-4. doi: 10.4103/0970-0218.74338. PubMed PMID: 21278861; PubMed Central PMCID: PMCPMC3026119.
8. National Health Mission. Five years (2009–2014). Achievements and new initiatives. New Delhi: Ministry of Health and Family Welfare, Government of India, 2014.
9. Lim SS, Dandona L, Hoisington JA, James SL, Hogan MC, Gakidou E. India's Janani Suraksha Yojana, a conditional cash transfer programme to increase births in health facilities: an impact evaluation. Lancet. 2010;375(9730):2009-23. Epub 2010/06/24. doi: 10.1016/s0140-6736(10)60744-1. PubMed PMID: 20569841.
10. Powell-Jackson T, Mazumdar S, Mills A. Financial incentives in health: New evidence from India's Janani Suraksha Yojana. J Health Econ. 2015;43:154-69. doi: 10.1016/j.jhealeco.2015.07.001. PubMed PMID: 26302940.
11. Ng M, Misra A, Diwan V, Agnani M, Levin-Rector A, De Costa A. An assessment of the impact of the JSY cash transfer program on maternal mortality reduction in Madhya Pradesh, India. Glob Health Action. 2014;7:24939. doi: 10.3402/gha.v7.24939. PubMed PMID: 25476929; PubMed Central PMCID: PMCPMC4256523.
12. Million Death Study C. Changes in cause-specific neonatal and 1-59-month child mortality in India from 2000 to 2015: a nationally representative survey. Lancet. 2017. doi: 10.1016/S0140-6736(17)32162-1. PubMed PMID: 28939096.
13. Leslie HH, Fink G, Nsona H, Kruk ME. Obstetric Facility Quality and Newborn Mortality in Malawi: A Cross-Sectional Study. PLoS medicine.

For peer review only - http://bmjopen.bmj.com/site/about/guidelines.xhtml

2016;13(10):e1002151. Epub 2016/10/19. doi: 10.1371/journal.pmed.1002151. PubMed PMID: 27755547; PubMed Central PMCID: PMC5068819.

14. Knight HE, Self A, Kennedy SH. Why are women dying when they reach hospital on time? A systematic review of the 'third delay'. PLoS One. 2013;8(5):e63846. Epub 2013/05/25. doi: 10.1371/journal.pone.0063846. PubMed PMID: 23704943; PubMed Central PMCID: PMCPMC3660500.

15. Patel R, Ladusingh L. Do Physical Proximity and Availability of Adequate Infrastructure at Public Health Facility Increase Institutional Delivery? A Three Level Hierarchical Model Approach. PLoS One. 2015;10(12). doi: 10.1371/journal.pone.0144352. PubMed PMID: 26689199; PubMed Central PMCID: PMCPMC4686327.

16. Kumar S, Dansereau E. Supply-side barriers to maternity-care in India: a facility-based analysis. PLoS One. 2014;9(8):e103927. Epub 2014/08/06. doi: 10.1371/journal.pone.0103927. PubMed PMID: 25093729; PubMed Central PMCID: PMCPMC4122393.

17. National Sample Survey Office, Ministry of Statistics and Programme Implementation. Key Indicators of Social Consumption in India: Health. New Delhi, India: Government of India, 2014.

18. Office of the Registrar General & Census Commissioner, Institute of Economic Growth. Annual Health Survey Report. New Delhi, India: Ministry of Home Affairs, Government of India, 2015.

19. International Institute for Population Sciences. District Level Household and Facility Survey - 4 State Fact Sheet (2012-13). Mumbai: Ministry of Health and Family Welfare, Government of India, 2014.

20. Patel V, Parikh R, Nandraj S, Balasubramaniam P, Narayan K, Paul VK, et al. Assuring health coverage for all in India. Lancet. 2015;386(10011):2422-35. doi: 10.1016/S0140-6736(15)00955-1. PubMed PMID: 26700532.

21. Dasgupta R, Bisht R. The Missing Mission in Health. Economic and Political Weekly. 2010;XLV(6):3.

22. Bhaumik S. India outlines plans for National Urban Health Mission. Lancet. 2012;380(9841):550. PubMed PMID: 22891372.

23. Programme Evaluation Organisation. Evaluation Study of National Rural Health Mission (NRHM) in Seven States. New Delhi, India: Planning Commission, Government of India, 2011.

24. National Health Mission, Ministry of Health & Family Welfare, Government of India. Indian Public Health Standards New Delhi, India: Government of India; [cited 2016 November 15]. Available from: http://nrhm.gov.in/nhm/nrhm/guidelines/indian-public-health-standards.html.

25. Ministry of Health and Family Welfare. Rural health statistics bulletin 2015. New Delhi: Government of India, 2015.

26. Zaman FA, Laskar NB. An application of Indian public health standard for evaluation of primary health centers of an EAG and a Non-EAG state. Indian journal of public health. 2010;54(1):36-9. Epub 2010/09/23. doi: 10.4103/0019-557x.70551. PubMed PMID: 20859050.

27. Sodani P, Sharma K. Assessing Indian Public Health Standards for 24 × 7 primary health centers: A case study with special reference to newborn care

For peer review only - http://bmjopen.bmj.com/site/about/guidelines.xhtml

services. The Journal of National Accreditation Board for Hospitals & Healthcare Providers. 2014;1(1):12-6. doi: 10.4103/2348-6139.132919.

28. Sodani PR, Sharma K. Assessing Indian public health standards for community health centers: a case study with special reference to essential newborn care services. Indian journal of public health. 2011;55(4):260-6. Epub 2012/02/03. doi: 10.4103/0019-557x.92402. PubMed PMID: 22298134.

29. Sodani PR, Sharma K. Strengthening Primary Level Health Service Delivery: Lessons from a State in India. Journal of Family Medicine and Primary Care. 2012;1(2):127-31. doi: 10.4103/2249-4863.104983. PubMed PMID: 24479021; PubMed Central PMCID: PMCPMC3893968.

30. Directorate General of Health Services. Indian Public Health Standards (IPHS) Guidelines for Primary Health Centers. New Delhi: Ministry of Health and Family Welfare, Government of India, 2012.

31. Bhatia M. The Indian Helath Care System, 2015. United States: The Commonwealth Fund, 2016.

32. Tuncalp, Were WM, MacLennan C, Oladapo OT, Gulmezoglu AM, Bahl R, et al. Quality of care for pregnant women and newborns-the WHO vision. BJOG. 2015;122(8):1045-9. doi: 10.1111/1471-0528.13451. PubMed PMID: 25929823; PubMed Central PMCID: PMCPMC5029576.

33. Donabedian A. The quality of care. How can it be assessed? JAMA. 1988;260(12):1743-8. PubMed PMID: 3045356.

34. Kruk ME, Leslie HH, Verguet S, Mbaruku GM, Adanu RM, Langer A. Quality of basic maternal care functions in health facilities of five African countries: an analysis of national health system surveys. The Lancet Global health. 2016;4(11):e845-e55. doi: 10.1016/S2214-109X(16)30180-2. PubMed PMID: 27670090.

35. Tripathi V, Stanton C, Strobino D, Bartlett L. Development and Validation of an Index to Measure the Quality of Facility-Based Labor and Delivery Care Processes in Sub-Saharan Africa. PLoS One. 2015;10(6):e0129491. Epub 2015/06/25. doi: 10.1371/journal.pone.0129491. PubMed PMID: 26107655; PubMed Central PMCID: PMC4479466.

36. Hsia RY, Mbembati NA, Macfarlane S, Kruk ME. Access to emergency and surgical care in sub-Saharan Africa: the infrastructure gap. Health Policy Plan. 2012;27(3):234-44. doi: 10.1093/heapol/czr023. PubMed PMID: 21441566.

37. WHO, UNFPA, UNICEF, Mailman School of Public Health. Monitoring emergency obstetric care: a handbook. Geneva: World Health Organization, 2009.

38. Rao M, Rao KD, Kumar AK, Chatterjee M, Sundararaman T. Human resources for health in India. Lancet. 2011;377(9765):587-98. doi: 10.1016/S0140-6736(10)61888-0. PubMed PMID: 21227499.

39. World Health Organization. Standard for improving quality of maternal and newborn care in health facilities. Geneva, Switzerland: World Health Organization Press; 2016.

40. Balarajan Y, Selvaraj S, Subramanian SV. Health care and equity in India. Lancet. 2011;377(9764):505-15. doi: 10.1016/S0140-6736(10)61894-6. PubMed PMID: 21227492; PubMed Central PMCID: PMCPMC3093249.

For peer review only - http://bmjopen.bmj.com/site/about/guidelines.xhtml

41. Barik D, Thorat A. Issues of Unequal Access to Public Health in India. Front Public Health. 2015;3:245. doi: 10.3389/fpubh.2015.00245. PubMed PMID: 26579507; PubMed Central PMCID: PMCPMC4621381.
42. Gupta A, Fledderjohann J, Reddy H, Raman VR, Stuckler D, Vellakkal S. Barriers and prospects of India's conditional cash transfer program to promote institutional delivery care: a qualitative analysis of the supply-side perspectives. BMC Health Serv Res. 2018;18(1):40. Epub 2018/01/27. doi: 10.1186/s12913-018-2849-8. PubMed PMID: 29370798; PubMed Central PMCID: PMCPMC5785836.
43. Kruk ME, Hermosilla S, Larson E, Mbaruku GM, Kruk ME, Hermosilla S, et al. Bypassing primary care clinics for childbirth: a cross-sectional study in the Pwani region, United Republic of Tanzania. Bulletin of the World Health Organization. 2014;92(4):246-53.
44. Prinja S, Gupta A, Verma R, Bahuguna P, Kumar D, Kaur M, et al. Cost of Delivering Health Care Services in Public Sector Primary and Community Health Centres in North India. PLoS One. 2016;11(8):e0160986. doi: 10.1371/journal.pone.0160986. PubMed PMID: 27536781; PubMed Central PMCID: PMCPMC4990301.
45. Jayanthi TP, Suresh S, Padmanaban P. Primary health centres: preferred option for birthing care in tamilnadu, India, from users' perspectives. J Health Popul Nutr. 2015;33(1):177-86. Epub 2015/05/23. PubMed PMID: 25995734; PubMed Central PMCID: PMCPMC4438661.
46. Straneo M, Hanson C, Fogliati P, Mbaruku GM. Minimum obstetric volume in low-income countries. The Lancet. 2017;389(10070):698. doi: 10.1016/S0140-6736(17)30342-2.
47. Randive B, Diwan V, De Costa A. India's Conditional Cash Transfer Programme (the JSY) to Promote Institutional Birth: Is There an Association between Institutional Birth Proportion and Maternal Mortality? PLoS One. 2013;8(6):e67452. Epub 2013/07/05. doi: 10.1371/journal.pone.0067452. PubMed PMID: 23826302; PubMed Central PMCID: PMC3694862.
48. Leone T. Demand and supply factors affecting the rising overmedicalization of birth in India. Int J Gynaecol Obstet. 2014;127(2):157-62. Epub 2014/07/30. doi: 10.1016/j.ijgo.2014.05.018. PubMed PMID: 25064013.
49. Vellakkal S, Reddy H, Gupta A, Chandran A, Fledderjohann J, Stuckler D. A qualitative study of factors impacting accessing of institutional delivery care in the context of India's cash incentive program. Soc Sci Med. 2017;178:55-65. Epub 2017/02/16. doi: 10.1016/j.socscimed.2017.01.059. PubMed PMID: 28199860; PubMed Central PMCID: PMCPMC5360172.
50. World Health Organization. WHO safe childbirth checklist implementation guide: improving the quality of facility-based delivery for mothers and newborns. Geneva: World Health Organization, 2015.
51. Das J, Hammer J. Quality of Primary Care in Low-Income Countries: Facts and Economics. Annual Review of Economics. 2014;6(1):525-53. doi: doi:10.1146/annurev-economics-080213-041350.
52. Harvey SA, Blandon YC, McCaw-Binns A, Sandino I, Urbina L, Rodriguez C, et al. Are skilled birth attendants really skilled? A measurement method, some disturbing results and a potential way forward. Bull World Health Organ.

For peer review only - http://bmjopen.bmj.com/site/about/guidelines.xhtml

2007;85(10):783-90. PubMed PMID: 18038060; PubMed Central PMCID: PMCPMC2636500.
53. Bohren MA, Vogel JP, Hunter EC, Lutsiv O, Makh SK, Souza JP, et al. The Mistreatment of Women during Childbirth in Health Facilities Globally: A Mixed-Methods Systematic Review. PLoS medicine. 2015;12(6):e1001847; discussion e. Epub 2015/07/01. doi: 10.1371/journal.pmed.1001847. PubMed PMID: 26126110; PubMed Central PMCID: PMCPmc4488322.
54. Campbell OM, Calvert C, Testa A, Strehlow M, Benova L, Keyes E, et al. The scale, scope, coverage, and capability of childbirth care. Lancet. 2016. doi: 10.1016/S0140-6736(16)31528-8. PubMed PMID: 27642023.
55. Leslie HH, Sun Z, Kruk ME. Association between infrastructure and observed quality of care in 4 healthcare services: A cross-sectional study of 4,300 facilities in 8 countries. PLoS medicine. 2017;14(12):e1002464. doi: 10.1371/journal.pmed.1002464.
56. Souza JP, Gülmezoglu AM, Vogel J, Carroli G, Lumbiganon P, Qureshi Z, et al. Moving beyond essential interventions for reduction of maternal mortality (the WHO Multicountry Survey on Maternal and Newborn Health): a cross-sectional study. The Lancet. 2013;381(9879):1747-55 %U http://www.sciencedirect.com/science/article/pii/S0140673613606868.
57. Semrau KEA, Hirschhorn LR, Marx Delaney M, Singh VP, Saurastri R, Sharma N, et al. Outcomes of a Coaching-Based WHO Safe Childbirth Checklist Program in India. New England Journal of Medicine. 2017;377(24):2313–24. doi: 10.1056/NEJMoa1701075.
58. Das J, Mohpal A. Socioeconomic Status And Quality Of Care In Rural India: New Evidence From Provider And Household Surveys. Health Aff (Millwood). 2016;35(10):1764-73. doi: 10.1377/hlthaff.2016.0558. PubMed PMID: 27702947.

For peer review only - http://bmjopen.bmj.com/site/about/guidelines.xhtml

Table 1: Indian Public Health Standard for basic delivery and newborn care, and corresponding question (closest match) in the DLHS-4 facility survey for Primary and Community Health Centers

Indian Public Health Standard	DLHS Question	Data Type	Categorization
**Management of normal deliveries**	Can you tell me whether normal delivery services are provided in this facility?	Self-reported	-
**Assisted vaginal deliveries including forceps/vacuum delivery whenever required**	Can you tell me whether assisted (forceps delivery/vacuum) delivery services are provided in this facility?	Self-reported	Process
**24-hour delivery services both normal and assisted**	Whether deliveries are conducted in this facility or no? If yes, whether the deliveries are conducted 24X7?	Self-reported	-
**Manual removal of placenta**	NA		
**Appropriate & prompt referral for cases needing specialist care**	NA		
**Management of pregnancy induced hypertension & referral**	NA		
**Pre-referral management (obstetric first-aid) in obstetric emergencies that need expert assistance**	NA		
**Proficient in identification and basic first aid treatment for PPH, eclampsia, sepsis and prompt referral**	Can you tell me whether administration of parenteral antibiotics is provided in this facility?	Self-reported	Process
	Can you tell me whether administration of parenteral oxytocics is provided in this facility?	Self-reported	Process
	Can you tell me whether administration of parenteral magnesium sulphate is provided in this facility?	Self-reported	Process
	Can you tell me whether management of PPH is provided in this facility?	Self-reported	Process
**Minimum 48 hours of stay after delivery**	NA		
**Facilities for Essential New Born Care and Resuscitation (Newborn Care Corner in LR/OT)**	Whether the following essential newborn care services are available? Resuscitation	Self-reported	Process
	Availability of designated newborn baby corner (functional)	Observed	-

For peer review only - http://bmjopen.bmj.com/site/about/guidelines.xhtml

**Management of neonatal hypothermia, infection protection, cord care & identification of sick newborn & prompt referral**	Whether the following essential newborn care services are available? Thermal protection (warmer/table lamp)	Self-reported	Process
**Early initiation of breastfeeding within one hour of birth**	NA		
**At least one medical officer - MBBS**	Availability of human resources (Medical Officer - Regular) - No. in position	Self-reported	Structure: human resources
**Three nurse midwife (staff-nurse)**	Availability of human resources (ANM/Female Health Worker - Regular) - No. in position	Self-reported	Structure: human resources
**Health worker female**	NA		
**Health Assistant (female)**	NA		
**Training for staff for emergency management to be ensured**	Training received by any medical officer during last five years (SBA or Basic Emergency Obstetric Care training)	Self-reported	Structure: human resources
	Training received for paramedical staff during last five years (SBA training)	Self-reported	Structure: human resources
**Labor table**	Physically verify and record availability of labor table with McIntosh sheet	Observed	Structure: facilities and supplies for functional labor room
**Managing labor using partograph**	Partographs being recorded for the recently delivered women or women in labor at the facility	Self-reported	Structure: facilities and supplies for functional labor room
**Suction machine**	Physically verify and record availability of suction machine (functional)	Observed	Structure: facilities and supplies for functional labor room
**Facility for oxygen administration**	NA		Structure: facilities and supplies for functional labor room
**Sterilization equipment (autoclave)**	Physically verify and record availability of autoclave/sterilizer (functional)	Observed	Structure: facilities and supplies for functional labor room
**24-hour running water**	Physically verify and record availability of 24 hour running water supply	Observed	Structure: facilities and supplies for functional labor room
**Electricity supply with back-up facility**	Whether the generator supply is connected to the LR	Observed	Structure: facilities and

For peer review only - http://bmjopen.bmj.com/site/about/guidelines.xhtml

**(generator with POL)**			supplies for functional labor room
**Attached toilet facilities**	Physically verify and record availability of attached toilet in the LR (functional)	Observed	Structure: facilities and supplies for functional labor room
**Delivery kits, including those for normal & assisted deliveries**	Normal delivery kits available in the facility?	Self-reported	Structure: facilities and supplies for functional labor room
**Privacy of woman ensured**	Observe and record the condition of the LR - privacy in the LR (satisfactory)	Observed	Structure: facilities and supplies for functional labor room
**Radiant warmer, fixed height with trolley**	Radiant warmer	Observed	Structure: equipment and supplies for newborn care
**Resuscitation bag and mask with reservoir**	Ambu bag with mask (functional)	Observed	Structure: equipment and supplies for newborn care
**Weighing scale**	Baby weighing machine of any time (functional)	Observed	Structure: equipment and supplies for newborn care
**Thermometer, clinical, digital**	NA		Structure: equipment and supplies for newborn care
**Light examination**	NA		Structure: equipment and supplies for newborn care
**I/V Cannula 24G, 26G**	Suction catheter/cannula (functional)	Observed	Structure: equipment and supplies for newborn care
**Mucus extractor; Pump suction, foot operated**	Pedal suction machine/mucus extractor (functional)	Observed	Structure: equipment and supplies for newborn care
**Feeding tube**	NA		
**Oxygen catheter**	NA		
**Sterile gloves**	Whether following emergency drugs and consumables are available: at least 2 pairs of gloves	Self-reported	Structure: emergency drugs and supplies for labor and delivery
**Inj. Oxytocin**	Whether following emergency drugs and consumables are available: oxytocin injection	Self-reported	Structure: emergency drugs and supplies for labor and delivery
**Inj. Diasepam**	Whether following emergency drugs and consumables are available: diazepam injection	Self-reported	Structure: emergency drugs and supplies for labor and delivery

For peer review only - http://bmjopen.bmj.com/site/about/guidelines.xhtml

**Tab. Nifedepine**	Whether following emergency drugs and consumables are available: nifedipine tablet	Self-reported	Structure: emergency drugs and supplies for labor and delivery
**Inj. Magnesium Sulphate**	Whether following emergency drugs and consumables are available: magnesium sulphate injection	Self-reported	Structure: emergency drugs and supplies for labor and delivery
**Inj. Lignocaine hydrochloride**	Whether following emergency drugs and consumables are available: lignocaine hydrochloride injection	Self-reported	Structure: emergency drugs and supplies for labor and delivery
**Inj. Methyl ergometrine maleate**	NA		Structure: emergency drugs and supplies for labor and delivery
**I/V Haemaccel**	NA		Structure: emergency drugs and supplies for labor and delivery
**Sterilized cotton and gauze**	Whether following emergency drugs and consumables are available: sterilized cotton and gauze	Self-reported	Structure: emergency drugs and supplies for labor and delivery
**Syringe**	Whether following emergency drugs and consumables are available: sterile syringes and needles (different sizes)	Self-reported	Structure: emergency drugs and supplies for labor and delivery
**Hub cutter**	Hub Cutter (available and functional)	Observed	Structure: emergency drugs and supplies for labor and delivery
**Oxygen bottles**	Whether following emergency drugs and consumables are available: Oxygen cylinder with face mask, wrench & regulator - functional	Self-reported	Structure: emergency drugs and supplies for labor and delivery

Indicators were extracted from Indian Public Health Standards (IPHS) for Primary Health Center;
DLHS questions from both Primary and Community Health Center surveys;
LR: Labor Room;
NA refers to IPHS indicators that were not included in the DLHS facility survey.

For peer review only - http://bmjopen.bmj.com/site/about/guidelines.xhtml

Figure Titles and Legends

Figure 1: Availability of basic delivery and newborn services in primary care facilities in India

Figure 2: Capacity of Primary Health Centers and Community Health Centers for basic delivery and newborn care

*** Legend:
Overall facility capacity of basic delivery and newborn care calculated as the average of the two preceding summary measures (process and structural capacity). Details on indicators of structure are available in Supplement Figure 1. Differences between PHC and CHC, for all indicators and summary index are statistically significant at $p<0.05$ level (Pearson's chi-square test and Student's t-test).

Figure 3: Difference in facility capacity for basic delivery and newborn care across rural and urban Primary Health Centers

***Legend: Differences between rural and urban facilities for all three summary indices are not statistically significant at $p<0.05$ level (Student's t-test).

Figure 4: Difference in facility capacity for basic delivery and newborn care across rural and urban Community Health Centers

Figure 5: Empowered Action Group (EAG) status and facility capacity for basic delivery and newborn care

Supplement Figure 1: Indicators of basic structural capacity for delivery and newborn care in primary facilities

Supplement Figure 2: Mean facility capacity scores in Empowered Action Group (EAG) states

***Legend: Mean facility capacity scores were calculated by summarizing overall capacity at district level, weighting facilities by total deliveries

Supplement Figure 3: Mean facility capacity scores in non-EAG states

***Legend: Mean facility capacity scores were calculated by summarizing overall capacity at district level, weighting facilities by total deliveries

For peer review only

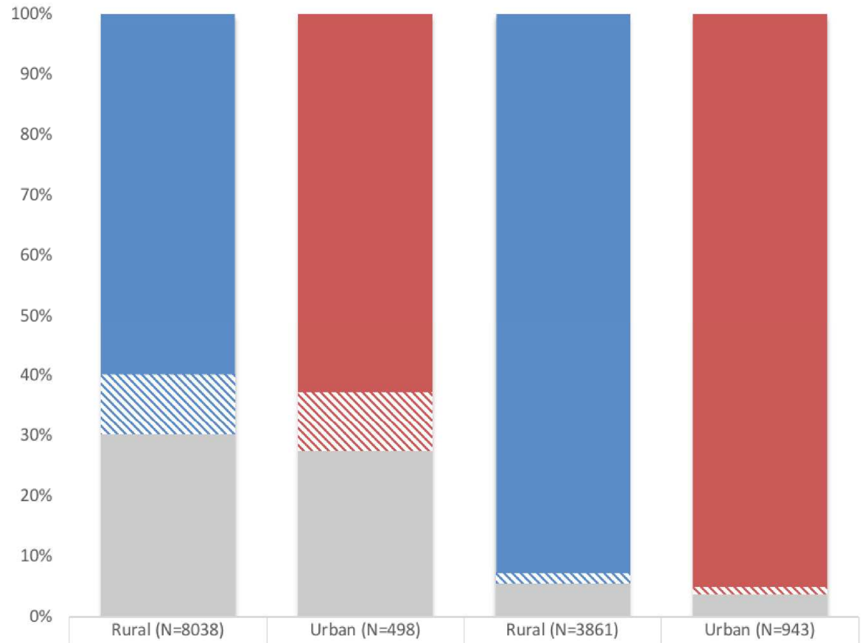

	Primary Health Center		Community Health Center	
	Rural (N=8038)	Urban (N=498)	Rural (N=3861)	Urban (N=943)
24X7	59.7%	62.7%	92.7%	94.9%
Daytime only	10.0%	9.8%	1.8%	1.3%
No service	30.4%	27.5%	5.6%	3.8%

Figure 1: Availability of basic delivery and newborn services in primary care facilities in India

333x296mm (300 x 300 DPI)

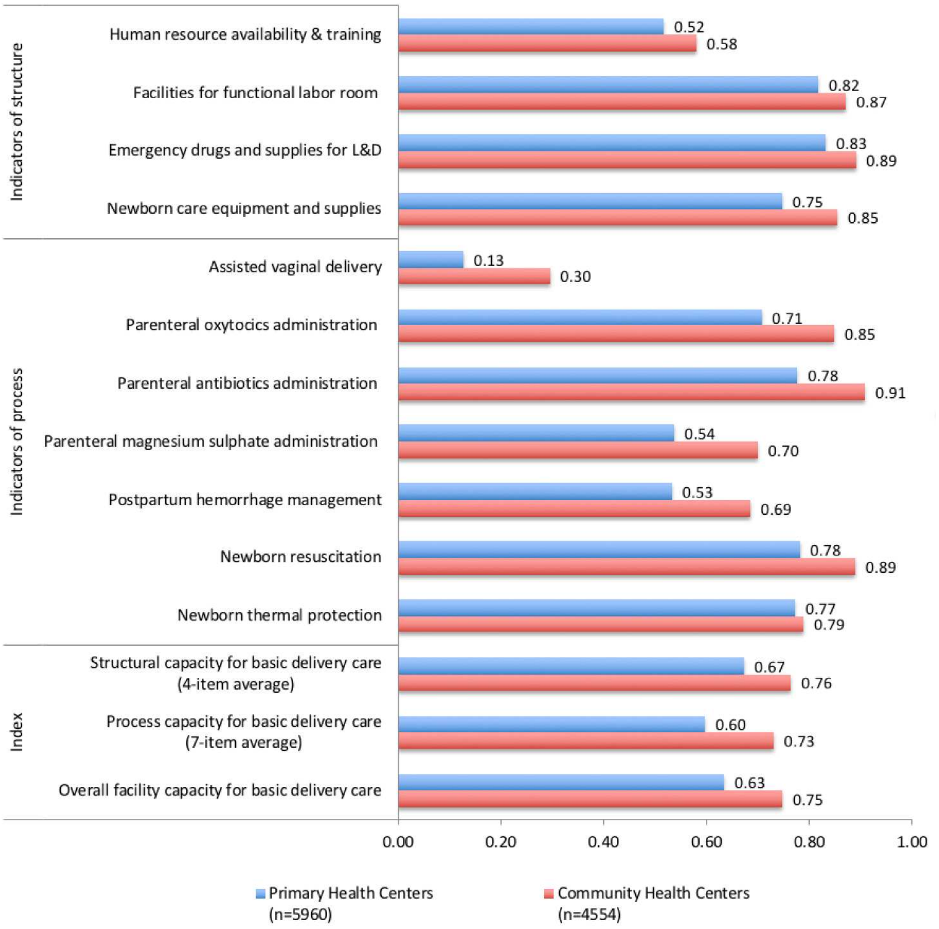

Figure 2: Capacity of Primary Health Centers and Community Health Centers for basic delivery and newborn care

*** Legend:
Overall facility capacity of basic delivery and newborn care calculated as the average of the two preceding summary measures (process and structural capacity). Details on indicators of structure are available in Supplement Figure 1. Differences between PHC and CHC, for all indicators and summary index are statistically significant at $p<0.05$ level (Pearson's chi-square test and Student's t-test).

333x351mm (300 x 300 DPI)

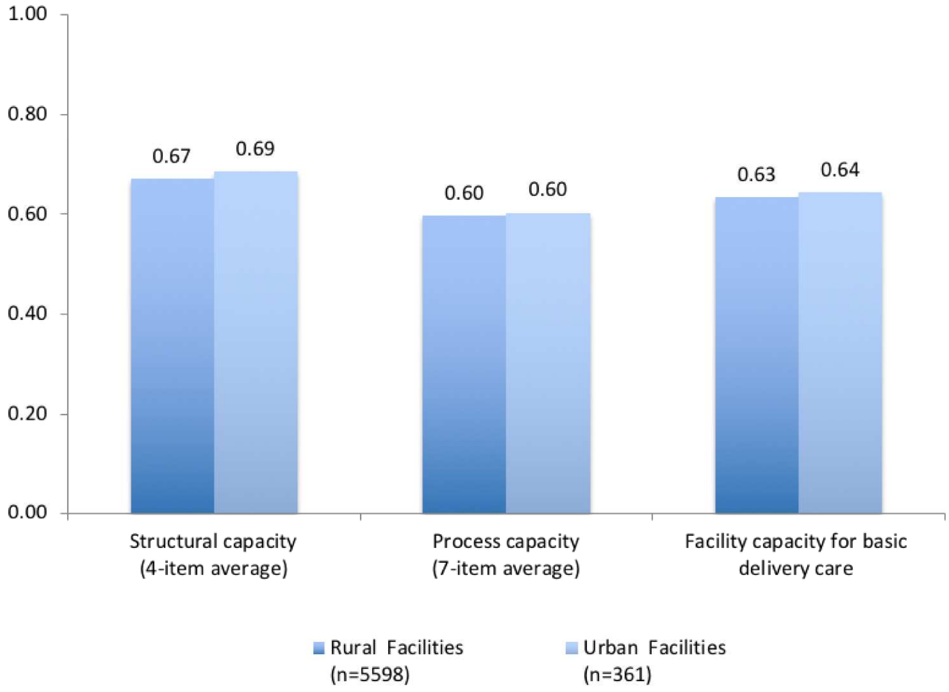

Figure 3: Difference in facility capacity for basic delivery and newborn care across rural and urban Primary Health Centers

***Legend: Differences between rural and urban facilities for all three summary indices are not statistically significant at p<0.05 level (Student's t-test).

288x217mm (300 x 300 DPI)

For peer review only - http://bmjopen.bmj.com/site/about/guidelines.xhtml

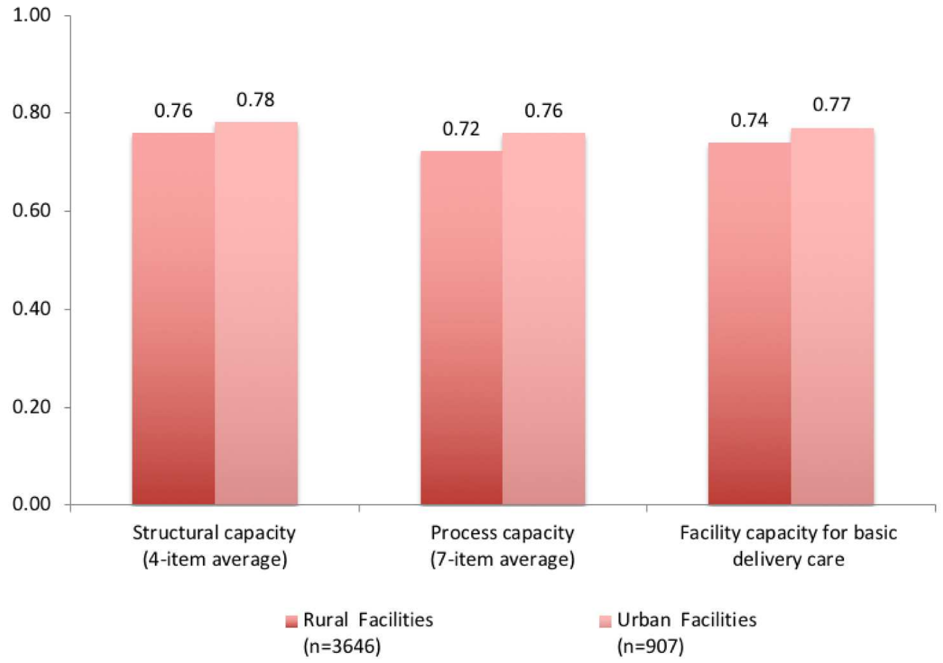

Figure 4: Difference in facility capacity for basic delivery and newborn care across rural and urban Community Health Centers

294x216mm (300 x 300 DPI)

For peer review only - http://bmjopen.bmj.com/site/about/guidelines.xhtml

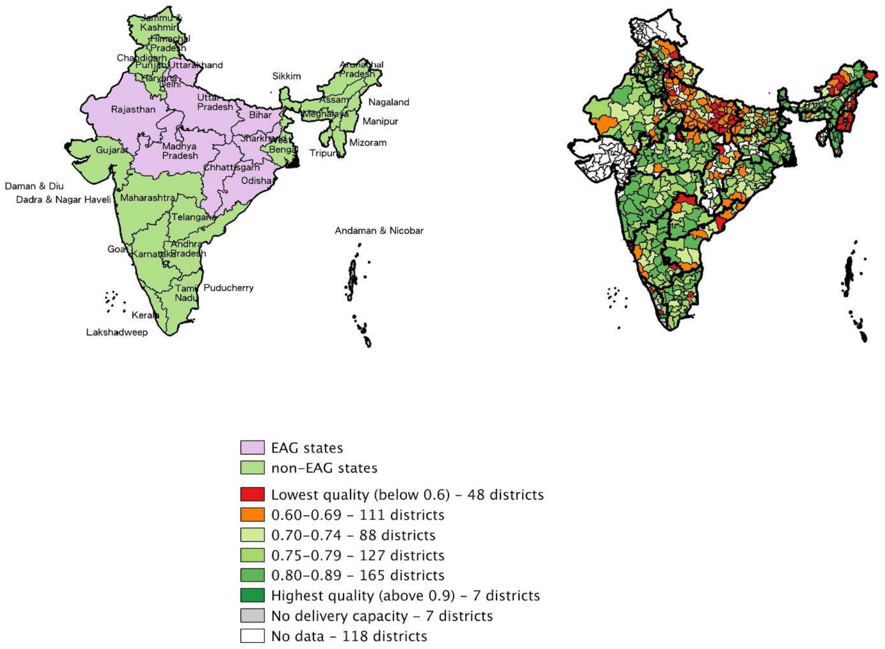

Figure 5: Empowered Action Group (EAG) status and facility capacity for basic delivery and newborn care

297x210mm (300 x 300 DPI)

For peer review only - http://bmjopen.bmj.com/site/about/guidelines.xhtml

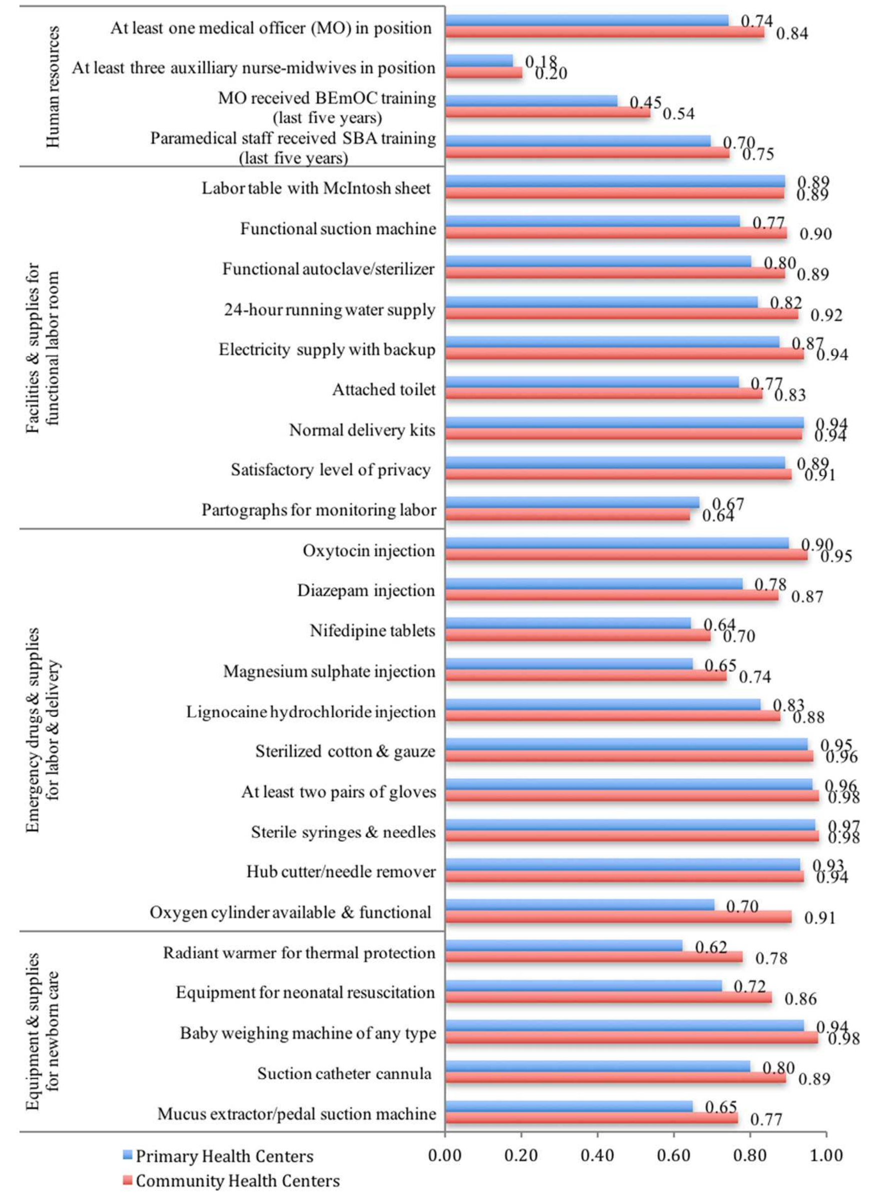

Supplement Figure 1: Indicators of basic structural capacity for delivery and newborn care in primary facilities

76x107mm (300 x 300 DPI)

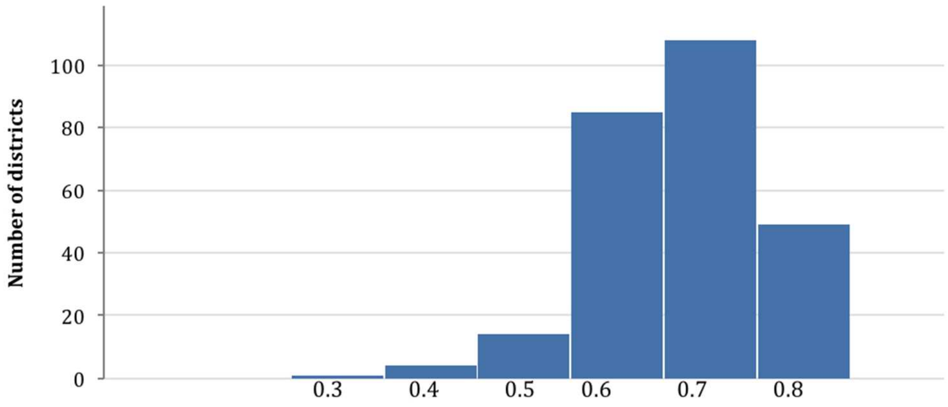

Supplement Figure 2: Mean facility capacity scores in Empowered Action Group (EAG) states

***Legend: Mean facility capacity scores were calculated by summarizing overall capacity at district level, weighting facilities by total deliveries

84x35mm (300 x 300 DPI)

For peer review only

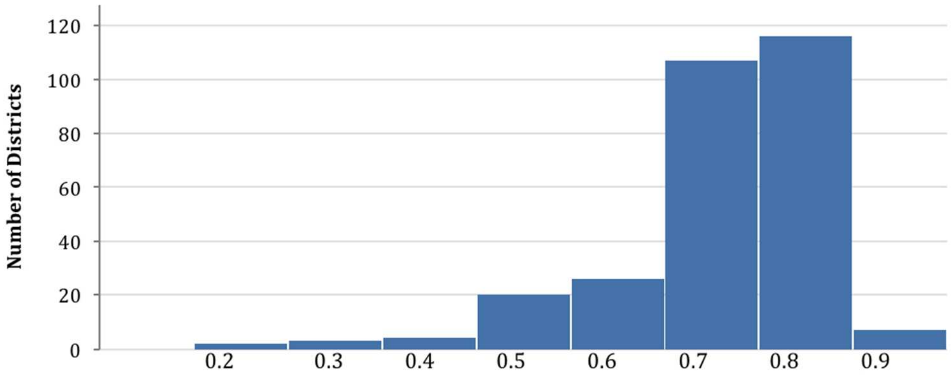

Supplement Figure 3: Mean facility capacity scores in non-EAG states

***Legend: Mean facility capacity scores were calculated by summarizing overall capacity at district level, weighting facilities by total deliveries

87x34mm (300 x 300 DPI)

For peer review only

STROBE Statement—Checklist of items that should be included in reports of ***cross-sectional studies***

	Item No	Recommendation	Page No. and Line No.
**Title and abstract**	1	(*a*) Indicate the study's design with a commonly used term in the title or the abstract	Page 1, lines 1-2
		(*b*) Provide in the abstract an informative and balanced summary of what was done and what was found	Pages 2-3
**Introduction**			
Background/rationale	2	Explain the scientific background and rationale for the investigation being reported	Pages 6-9
Objectives	3	State specific objectives, including any prespecified hypotheses	Page 9, lines 6-12
**Methods**			
Study design	4	Present key elements of study design early in the paper	Pages 9 - 12
Setting	5	Describe the setting, locations, and relevant dates, including periods of recruitment, exposure, follow-up, and data collection	Page 9 line 16 – page 10 line 9
Participants	6	(*a*) Give the eligibility criteria, and the sources and methods of selection of participants	Page 9 line 16 – page 10 line 9
Variables	7	Clearly define all outcomes, exposures, predictors, potential confounders, and effect modifiers. Give diagnostic criteria, if applicable	Page 10 line 11 – page 12 line 18
Data sources/ measurement	8*	For each variable of interest, give sources of data and details of methods of assessment (measurement). Describe comparability of assessment methods if there is more than one group	Pages 9 - 12
Bias	9	Describe any efforts to address potential sources of bias	NA
Study size	10	Explain how the study size was arrived at	Page 9 line 16 – 19
Quantitative variables	11	Explain how quantitative variables were handled in the analyses. If applicable, describe which groupings were chosen and why	Page 10 line 12 – Page12 line 18
Statistical methods	12	(*a*) Describe all statistical methods, including those used to control for confounding	Page 12 line 21 – Page 13 line 17
		(*b*) Describe any methods used to examine subgroups and interactions	Page 13 lines 10-17
		(*c*) Explain how missing data were addressed	Page 11 lines 1-5
		(*d*) If applicable, describe analytical methods taking account of sampling strategy	NA
		(*e*) Describe any sensitivity analyses	NA
**Results**			
Participants	13*	(a) Report numbers of individuals at each stage of study—eg numbers potentially eligible, examined for eligibility, confirmed eligible, included in the study, completing follow-up, and analysed	Page 14 line 5-22
		(b) Give reasons for non-participation at each stage	Page 14 line 5-22
		(c) Consider use of a flow diagram	NA
Descriptive data	14*	(a) Give characteristics of study participants (eg	Page 14 lines 17-22

For peer review only

		demographic, clinical, social) and information on exposures and potential confounders	
		(b) Indicate number of participants with missing data for each variable of interest	Page 14 lines 5-11
Outcome data	15*	Report numbers of outcome events or summary measures	Page 15 lines 1-7
Main results	16	(*a*) Give unadjusted estimates and, if applicable, confounder-adjusted estimates and their precision (eg, 95% confidence interval). Make clear which confounders were adjusted for and why they were included	Page 15 lines 1 – Page 16 line 11
		(*b*) Report category boundaries when continuous variables were categorized	Page 16, line 7-11
		(*c*) If relevant, consider translating estimates of relative risk into absolute risk for a meaningful time period	NA
Other analyses	17	Report other analyses done—eg analyses of subgroups and interactions, and sensitivity analyses	NA
**Discussion**			
Key results	18	Summarise key results with reference to study objectives	Page 16 line 14 – Page 17 line 2
Limitations	19	Discuss limitations of the study, taking into account sources of potential bias or imprecision. Discuss both direction and magnitude of any potential bias	Page 20 line 5 - 22
Interpretation	20	Give a cautious overall interpretation of results considering objectives, limitations, multiplicity of analyses, results from similar studies, and other relevant evidence	Page 22 line 7 – 20
Generalisability	21	Discuss the generalisability (external validity) of the study results	Page 20 line 16 – 22
**Other information**			
Funding	22	Give the source of funding and the role of the funders for the present study and, if applicable, for the original study on which the present article is based	Page 4 lines 18-20

For peer review only

For peer review only - http://bmjopen.bmj.com/site/about/guidelines.xhtml

BMJ Open

Can India’s primary care facilities deliver? A cross-sectional assessment of the Indian public health system’s capacity for basic delivery and newborn services

Journal:	*BMJ Open*
Manuscript ID	bmjopen-2017-020532.R2
Article Type:	Research
Date Submitted by the Author:	22-Mar-2018
Complete List of Authors:	Sharma, Jigyasa; Harvard T. H. Chan School of Public Health, Global Health and Population<br>Leslie, Hannah; Harvard T. H. Chan School of Public Health, Global Health and Population<br>Regan, Mathilda; Harvard School of Public Health, Global Health and Population<br>Nambiar, Devaki; The George Institute for Public Health, India<br>Kruk, Margaret; Harvard School of Public Health,
**Primary Subject Heading**:	Health services research
Secondary Subject Heading:	Health policy, Global health, Health services research
Keywords:	Health policy < HEALTH SERVICES ADMINISTRATION & MANAGEMENT, Quality in health care < HEALTH SERVICES ADMINISTRATION & MANAGEMENT, Health Systems, Maternal Health, Newborn Health

SCHOLARONE™
Manuscripts

For peer review only

Can India's primary care facilities deliver? A cross-sectional assessment of the Indian public health system's capacity for basic delivery and newborn services

Jigyasa Sharma, MSc[1], Hannah H Leslie, PhD[1], Mathilda Regan, MPH[1], Devaki Nambiar, PhD[2], Margaret E Kruk, MD[1]

[1]Department of Global Health and Population, Harvard T.H. Chan School of Public Health, USA
[2] The George Institute for Public Health, India

Corresponding Author:
Jigyasa Sharma
Department of Global Health and Population
Harvard T.H. Chan School of Public Health
655 Huntington Avenue
Boston, MA 02115
jigyasa.sharma@mail.harvard.edu

For peer review only

For peer review only - http://bmjopen.bmj.com/site/about/guidelines.xhtml

Abstract

Objectives

To assess input and process capacity for basic delivery and newborn (intrapartum care hereafter) care in the Indian public health system and to describe differences in facility capacity between rural and urban areas and across states.

Design

Cross-sectional study.

Setting

Data from the nationally representative 2012-2014 District Level Household and Facility Survey, which includes a census of community health centers (CHCs) and sample of primary health centers (PHCs) across 30 states and union territories in India.

Participants

8536 PHCs and 4810 CHCs.

Outcome Measures

We developed a summative index of 33 structural and process capacity items matching the Indian Public Health Standards for PHCs as a metric of minimum facility capacity for intrapartum care. We assessed differences in performance on this index across facility type and location.

Results

About 30% of PHCs and 5% of CHCs reported not offering any intrapartum care. Amongst those offering services, volumes were low: median monthly delivery volume was 8 (IQR=13) in PHCs and 41 (IQR=73) in CHCs. Both PHCs and CHCs failed to meet the national standards for basic intrapartum care capacity. Mean facility capacity

For peer review only - http://bmjopen.bmj.com/site/about/guidelines.xhtml

was low in PHCs in both urban (0.64) and rural (0.63) areas, while in CHCs, capacity was slightly higher in urban areas (0.77 vs. 0.74). Gaps were most striking in availability of skilled human resources and emergency obstetric services. Poor capacity facilities were more concentrated in the more impoverished states, with 37% of districts from these states receiving scores in the lowest third of the facility capacity index (<0.70), compared to 21% of districts otherwise.

Conclusions

Basic intrapartum care capacity in Indian public primary care facilities is weak in both rural and urban areas, especially lacking in the poorest states with worst health outcomes. Improving maternal and newborn health outcomes will require focused attention to quality measurement, accountability mechanisms, and quality improvement. Policies to address deficits in skilled providers and emergency service availability are urgently required.

For peer review only

Strengths and limitations of this study

1. This study uses a nationally representative survey to assess primary public health facilities' adherence to nationally set minimum recommended standards for basic delivery and newborn (intrapartum) care.
2. The index used to assess input and process capacity is based on national guidelines, and therefore represents a contextually appropriate measure of basic service readiness.
3. However, the index lacked information on important technical and interpersonal care processes, such as provider competence, owing to data limitation.
4. The sample did not include private facilities and included limited information on quality of referral care.

Ethical approval:

The Harvard University Human Research Protection Program approved this analysis as exempt from human subjects review.

Funding statement

This research received no specific grant from any funding agency in the public, commercial or not-for-profit sectors.

Competing interests statement

We have read and understood BMJ policy on declaration of interests and declare that we have no competing interests.

For peer review only - http://bmjopen.bmj.com/site/about/guidelines.xhtml

Author contributions

JS, HHL and MEK conceived the research question. JS and HHL developed the methods, and JS, HHL and MR conducted the data analysis. JS, HHL, MR, DN and MEK interpreted the results, and JS wrote the first draft of the manuscript. JS, HHL, MR, DN and MEK edited and revised the manuscript and approved the final draft for submission.

Data sharing statement

The data are publicly available through the International Institute of Population Sciences, India. Website: http://www.iipsindia.ac.in

For peer review only

Introduction

The adoption of Sustainable Development Goals (SDGs) has reaffirmed the reduction of preventable maternal and newborn deaths as global health priorities. Achieving the targeted reduction of the global maternal mortality ratio (MMR) to less than 70 per 100,000 live births and neonatal mortality to 12 per 1,000 live births [1] will require near universal coverage of institutional deliveries and timely detection and management of birth complications, as most maternal and newborn deaths occur at birth or within 24 hours of birth [2]. An emerging body of literature suggests that getting women to facilities alone will not suffice: what happens when a woman reaches a facility – including her right to quality antenatal, intrapartum, and postpartum care – matters [3, 4], especially given the global mandate of universal health coverage [5, 6].

In India, increased coverage of facility-based births has not successfully translated to desired improvement in health outcomes for mothers and newborns. Under India's National Rural Health Mission (NRHM, now called the National Health Mission or NHM), a variety of interventions were introduced through architectural improvements in the fund flow and design of services: increased number of maternal care facilities, particularly primary health centers (PHCs) and community health centers (CHCs); a strengthened supply chain for essential medicines, equipment and supplies; and Janani Surakshya Yojana (JSY), a financial-incentive program to increase institutional deliveries [7]. Following the launch of NRHM in 2005, institutional deliveries in rural areas have more than doubled [8], and a declining trend in maternal and newborn mortality has been noted, although strong causal evidence linking NRHM efforts to improved health

For peer review only

outcomes for mothers and newborns is lacking [9-11]. Annual decline in neonatal mortality between 2005 and 2015 was faster than in the preceding years; however, the rate of decline is not sufficient to meet the 2030 SDG targets [12]. Inadequate quality of care, including insufficient facility readiness, and low provider skill and clinical management capacity, as evidence from low and middle income (LMIC) countries indicates, may explain why increased utilization alone may not have resulted in the desired reduction in adverse intrapartum outcomes [13, 14]. Moreover, quality of care itself also affects utilization. Evidence from India indicates that the availability of a labor room and adequacy of essential equipment and laboratory services for childbirth at public health facilities have a significant effect on service uptake [15, 16]. Describing and improving quality of intrapartum care is relevant to improving service uptake in India, where maternal and newborn services are underutilized despite the availability of primary healthcare in public health facilities free of charge.

Public health facilities are a significant provider of care in India, especially for rural and vulnerable population segments. About 80.1% of all deliveries in rural India are facility-based, of which about 70% are in public facilities [17]. For urban areas, 89.5% of births are institutional, 47.4% of which are in public facilities [17]. In the majority of India's states, more facility-based deliveries happen at government health institutions than in private facilities [18, 19]. Additionally, quality of care in the public sector affects the poorest segment of the population the most, as the poorest wealth quintile class is more heavily reliant on public health facilities than the richest, in both rural (58% vs. 29%) and urban areas (48% vs. 19%) [17, 20].

For peer review only - http://bmjopen.bmj.com/site/about/guidelines.xhtml

India has made several efforts to strengthen its public health service provision. Initial policy emphasis was on rural areas given the preponderance of the Indian population living there and the assumption that population access to quality health services was relatively better in urban areas. However, mounting evidence of worse health outcomes for the urban poor compared to the urban rich - and sometimes even rural populations - led to the formal launch of the National Urban Health Mission in 2013 [21]. The unified National Health Mission is now charged with oversight for public health care in urban and rural areas [22]. The implementation of programs and policies, however, has varied sub-nationally. Under the NRHM, substantial funding and technical support has been provided to states with relatively weaker public health indicators and health system infrastructure, including the Empowered Action Group (EAG) states (Bihar, Jharkhand, Chhattisgarh, Madhya Pradesh, Uttar Pradesh, Uttaranchal, Orissa and Rajasthan) [23]. About 46% of India's population resides in the EAG states, and this region lags behind the rest of the Indian states on socio-economic, demographic and health indicators [23].

To boost the performance of public health facilities, in 2007 the government introduced the Indian Public Health Standards (IPHS), a set of recommendatory standards to be used as a reference for public health infrastructure planning and as a benchmark for assessing the functional status of health facilities [24]. Although widely used in health system planning and management, the use of these standards in health system research has been limited in number and geographic scope to date. Rural health statistics from 2015 note that only 21% of PHCs and 26% of CHCs were functioning in concordance with IPHS

For peer review only - http://bmjopen.bmj.com/site/about/guidelines.xhtml

standards, although a service-specific breakdown was not provided [25]. A few studies have utilized IPHS as a reference, noting deficiencies in service availability, human resource and infrastructure [26-29]. With one exception [29], all these studies surveyed less than a hundred facilities.

The aim of this work is to understand the capacity of the Indian public health system to provide quality intrapartum care. This is an important element of understanding the effects of maternal and newborn health policies to date and setting priorities for health system strengthening going forward. In this paper, using the updated IPHS (2012) as the minimum standard for basic facility capacity for intrapartum care, we (1) assess the performance of PHCs and CHCs in India against this standard; and (2) describe differences in intrapartum care capacity between urban and rural areas, and across states.

Methods

Study sample

We drew data from the fourth cycle of the District Level Household and Facility Survey (DLHS) conducted by the International Institute of Population Sciences in India. We used data from the facility modules of the DLHS for PHCs and CHCs to capture the primary level of the public health system designated to provide intrapartum care services. Typically, PHCs serve a population of 20,000 to 30,000 [30], and CHCs, which are intended to be referral centers for four PHCs, cater to approximately a population of 80,000 to 120,000 [30]. Within the tiers of the Indian public health system, CHCs are referral centers for PHCs; yet they are frequently used for first-line intrapartum care. In

For peer review only - http://bmjopen.bmj.com/site/about/guidelines.xhtml

this paper, in keeping with international guidelines, both are referred to as primary care facilities [31].

The most recent survey, DLHS-4, was conducted in 2012-2014 and contains a sample of PHCs designed to be representative at district level and a census of CHCs. We also obtained the most recent district and state boundaries from the database of global administrative areas (GADM.org) and matched DLHS data to the appropriate area using state and district name. We were unable to match three districts, likely due to district boundary changes between 2013 and 2016.

Basic intrapartum care capacity index

Availability of essential physical and human resources and services is foundational to the provision of high-quality care [32]. The study objective was to evaluate basic intrapartum care capacity that should be present in all childbirth facilities. As described in the WHO quality of care framework for maternal and newborn health [32], which builds on Donebedian's framework of healthcare quality [33], structural capacity elements, such as availability of drugs and supplies, refer to necessary, but not sufficient, human and physical resource conditions for care delivery. Process capacity elements refer to facility's ability to offer evidence-based practices for routine care and management of complications, such as assisted vaginal delivery or administration of parenteral antibiotics.

We used the IPHS for PHCs to define intrapartum care capacity in this context; CHCs are assumed to meet at least the standards of the lower-tier PHCs [30]. The facility capacity

For peer review only - http://bmjopen.bmj.com/site/about/guidelines.xhtml

index comprises structure and process capacity elements required for both routine and basic emergency obstetric care.

We identified 48 elements of the IPHS pertaining to intrapartum care. Of these, we found at least some information on 33 in the DLHS facility audit, of which 26 were a complete match. Facility capacity to offer services such as manual removal of placenta, appropriate pre-referral management of and referral for obstetric emergencies, and availability of select drugs and equipment, identified as the basic requirements for all PHCs and higher-level facilities, were not included in the facility audit. See Table 1 for the full list of IPHS requirements along with corresponding item from DLHS survey.

Our data comprised both observed and self-reported measures of structural inputs to care and self-reported process capacity indicators. Structural inputs fell into four sub-domains: skilled provider availability (average of 4 indicators), facilities for a functional labor room (average of 9 indicators), emergency drugs and supplies for labor (average of 10 indicators), and delivery and newborn care equipment and supplies (average of 5 indicators). Process capacity indicators included reports from survey respondent (facility managers or medical officers in charge) on availability of assisted vaginal deliveries; administration of parenteral oxytocins, antibiotics, magnesium sulfate; postpartum hemorrhage management; newborn resuscitation and thermal protection services. All process capacity indicators were binary measures. The items were grouped following the IPHS categorization. See Supplemental Figure 1 for details on all items.

For peer review only - http://bmjopen.bmj.com/site/about/guidelines.xhtml

For both structure (4 sub-domains) and process (7 items) capacity indices, sub-domains or indicators were averaged to provide a facility summary score from 0 to 1, with missing values excluded. Missing values were minimal (less than 3% of facilities) for all indicators except for availability of newborn resuscitation (11% of PHCs missing) and electricity supply with backup (23.6% of PHC and 9.6% of CHC missing). We created an index of facility capacity for basic intrapartum care as an average of the structure and process capacity indices. We followed current practice and weighted each indicator equally within a sub-domain or index given the lack of guidance in the IPHS on weights [34, 35].

Covariates

We identified covariates that may be associated with facility capacity. We followed DLHS classification of facilities as either a PHC or CHC, which is based on delivery volume and their capacity to perform comprehensive obstetric emergency management. We categorized facilities by rural versus urban location as reported in the survey. We also calculated annual delivery volume based on facility report, excluding missing values. We calculated capacity for quality intrapartum care within districts by weighting each facility by delivery volume. For weighting, we assigned facilities reporting childbirth capacity but missing delivery volume data or reporting no deliveries a delivery volume of 1.

Statistical analysis

In this analysis, to describe primary level public health facilities' capacity to provide basic intrapartum care, we first calculated availability of basic childbirth care services at

For peer review only - http://bmjopen.bmj.com/site/about/guidelines.xhtml

the facility level: facilities not offering any services, facilities offering round-the-clock services, and those providing daytime services only. Some PHCs and CHCs may have curtailed services possibly due to low volume (resulting from proximity to a higher-level facility), inadequate infrastructure or human resources. As our data do not support classification of these facilities based on the reason for service unavailability (which may have been justified or not, given individual circumstances), we restricted subsequent analysis to facilities reporting provision of either round-the-clock or daytime only childbirth services. In this sample, we assessed each facility's adherence to national standard for care provision, stratified by facility type (PHC or CHC).

We described variation in facility-level capacity across rural and urban settings. We employed Pearson's chi-square test for binomial and Student's T-test for continuous indicators to assess the statistical significance ($p<0.05$) of difference in capacity between PHCs and CHCs and between rural and urban PHCs. To visualize differences in intrapartum care capacity across India, we also calculated averages of overall facility intrapartum care capacity at the district level, weighting facilities by total deliveries. For mapping, we divided the overall facility capacity index scores into sixths, designating the lowest third of scores as "poor capacity."

Statistical analysis was done using Stata 14.1 (StataCorp, Texas) and mapping using QGIS Version 2.12 (Free Software Foundation, Massachusetts).

Ethical approval

For peer review only - http://bmjopen.bmj.com/site/about/guidelines.xhtml

The Harvard University Human Research Protection Program approved this analysis as exempt from human subjects review.

Patient and Public Involvement

Patients or public were not involved in this analysis.

Results

The 2012-2013 DLHS-4 facility survey was conducted across India; however, data are publicly available only for 30 out of 36 states and union territories (excluding the states of Gujarat, and Jammu and Kashmir, and the territories Dadar and Nagar Haveli, Daman Diu, the National Capital Territory of Delhi, and Lakshadweep). Our study sample comprised a total of 8536 PHCs and 4810 CHCs. Although all PHCs and CHCs are expected to provide childbirth care, about 30.2% (N=2557) of PHCs and 5.2% (N=251) of CHCs reported not providing any childbirth services, either round-the-clock or daytime-only. Only 59.7% (N=4798) of rural and 62.7% (N=312) of urban PHCs and only 92.7% (N=3578) of rural and 94.9% (N=895) of urban CHCs offered 24-hour intrapartum care. As Figure 1 indicates, availability of services was similar for both levels of facility across urban and rural settings.

Within facilities offering any delivery services (N= 5959 PHC, N= 4553 CHC), median monthly delivery volume was 8 (IQR=13) in PHCs and 41 (IQR=73) in CHCs. All CHCs are expected to provide 24x7 delivery care; 73.4% of PHCs self-identified as government-designated 24x7 delivery facilities. In practice, 98% of CHCs and 86% of

For peer review only - http://bmjopen.bmj.com/site/about/guidelines.xhtml

PHCs actually offered 24x7 services. A designated area for newborn care was present only in 67% and 80% of included PHCs and CHCs, respectively.

Overall capacity for basic intrapartum care was lower than the basic IPHS standard in both PHCs (mean 0.63, standard deviation [SD] 0.23) and CHCs (mean 0.75, SD 0.17) included in this analysis. The structural capacity index for basic intrapartum care was slightly better than the process capacity index for both PHCs (mean 0.67, SD 0.19 vs. 0.60, SD 0.29) and CHCs (mean 0. 76, SD 0.14 vs. 0.73, SD 0.22). As shown in Figure 2, PHCs lagged behind CHCs on all indicators; the difference between PHCs and CHCs was statistically significant for all indicators and the summary index. Although CHCs, as the referral centers for PHCs, are expected to offer more comprehensive care, they frequently lacked basic infrastructure required even at PHCs. Human resource availability and training in both CHCs (mean 0.58, SD 0.23) and PHCs (mean 0.52, SD 0.26) were comparably low. Particularly large gaps were seen in process capacity indicators such as provision of assisted vaginal deliveries (13% of PHCs versus 30% of CHCs), administration of parenteral magnesium sulfate (54% versus 70%), and management of postpartum hemorrhage (53% of PHCs versus 69% of CHCs).

Figures 3 and 4 display the differences in structural and process capacity indices, and in overall capacity of basic intrapartum care across rural and urban settings, for PHCs and CHCs, respectively. Facility capacity was low across the board, with small differences across geographical settings. Scores for structural, process, and overall capacity summary indices were comparable for PHCs, with no significant rural-urban differences. Rural-

For peer review only - http://bmjopen.bmj.com/site/about/guidelines.xhtml

urban differences in CHCs were slightly more pronounced, with urban CHCs outperforming rural CHCs by 0.02 to 0.04 in structural, process, and overall facility capacity indices.

As shown in Figure 5, the capacity of facilities that offered intrapartum care varied across the country, with districts with the poorest facility capacity concentrated in the northern part of the country. There were seven districts with no facilities offering childbirth services in the DLHS data. We had no data for 118 districts, including the districts in regions for which survey data were not publicly available. In both EAG and non-EAG states, there was significant variation in facility capacity (Supplemental Figures 2 and 3). There was a disproportionate number of poor capacity facilities in EAG states, with 37% of districts scoring in the lowest third of the facility capacity index (less than 0.70) compared to 21% of non-EAG states. Among the nine EAG states, Uttar Pradesh had the greatest concentration of poor capacity facilities, with 84% percent of districts receiving scores in the lowest third.

Discussion

Using a national health facility survey, we found that many Indian public primary care facilities fail to meet the nationally set minimum recommended standards for basic intrapartum care. Despite a concerted effort to increase accessibility, about 30% of PHCs and 5% of CHCs did not offer any childbirth services; round-the-clock intrapartum care services were available only in 60% of PHCs and 94% of CHCs. Both PHCs and CHCs offering these services had critical deficiencies in routine and emergency care practices,

For peer review only - http://bmjopen.bmj.com/site/about/guidelines.xhtml

infrastructure and staffing, fulfilling an average of only 63% and 75% of an index of basic intrapartum care capacity, respectively. These findings are consistent with studies documenting weak emergency care capacity, infrastructure and staffing in low-income countries, particularly in lower level facilities with low delivery volume [34, 36]. Most CHCs, which are meant to serve as referral centers to manage more complicated cases [23], failed to meet the threshold set for lower level facilities.

Structural capacity across the study facilities was marginally better than process capacity, with large gaps in provision of signal functions of emergency obstetric care [37]. For example, assisted vaginal deliveries were offered only in about one-tenth of PHCs and one-third of CHCs. Almost one-half of PHCs and one-third of CHCs reported not providing services to manage postpartum hemorrhage, a leading cause of maternal mortality in India. We also noted that availability of medicine and supplies, such as the partograph or uterotonics, was worse than that of equipment and infrastructure, such as scales or labor tables, underscoring the need to strengthen the supply chain for essential medicine and supplies. This is a challenge that the NRHM was designed to address, and one that has been resistant to change [16].

Of similar concern are the low scores on human resource availability and training across facilities. Although addressing the shortage of skilled providers in rural PHCs and CHCs has also been one of the agendas of the NRHM, critical deficiencies clearly persist, as has been documented elsewhere [38]. The gaps observed are especially problematic because our indicators reflect a minimum level of human resource preparedness (for example,

For peer review only

skilled birth attendance training for auxiliary nurse-midwives or emergency obstetric care training for medical officers). They do not include key dimensions of provider availability and competence, such as appropriately diagnosing and managing complications, which the World Health Organization identifies as integral to good quality of care during facility-based deliveries [39]. For example, accounting for absenteeism, a common problem in public facilities in India, would probably lower our estimate of actual human resource availability significantly [38].

Facility capacity for basic intrapartum care in public facilities was similar in rural and urban areas. Our findings underline the need to improve facility capacity in both urban and rural areas, which the unified National Health Mission may be well situated to tackle. Given the fact that the public facilities assessed here are the main source of obstetric care for poor populations in both rural and urban settings, [40, 41] the deficiencies we found are likely to disproportionately affect the most vulnerable women. Meeting the commitment to equitable care inherent in the National Health Mission requires redoubled attention on care quality in the public health system.

Despite additional funding and considerable technical support for improving maternal and newborn health outcomes in the EAG states, facility capacity for intrapartum care remains weaker than in non-EAG states. We found that facilities with poor capacity were especially common in Uttar Pradesh and Bihar, states with the two highest maternal and newborn mortality rates in the country. Roughly comprising a third of India's population,

For peer review only - http://bmjopen.bmj.com/site/about/guidelines.xhtml

these two states are critical in India's progress towards meeting national and international maternal and newborn mortality reduction targets.

Our findings indicate very low monthly delivery volume in PHCs. Bypassing of care at lower-level facilities may be driven by a myriad of reasons [42], including, but not limited to, women's preference for higher quality care when it is available, as seen in Tanzania [43]. Regardless of the reason, underutilization of PHCs represents inefficiency in the Indian health system, considering the amount of resources spent at this level of healthcare [44]. This merits attention for effective planning and resource allocation as healthcare delivery structure being developed for urban India under the National Urban Health Mission follows the same model as in rural areas [22].

A demand for quality care, regardless of the level of facility, has been noted in India. Preference for higher-level facilities with better quality is observed in the state of Kerala in India [17], whereas in Tamil Nadu, women prefer to delivery in PHCs with qualified staff providing respectful care [45]. Most high-income and many middle-income countries encourage delivery in hospitals where surgical and newborn care services are available to all women [46]. Organizing health system services around the capacity to deliver high-quality care may thus respond to women's observed preferences and improve health system performance. The breadth of quality deficits in CHCs and especially PHCs, which are under-resourced despite concerted efforts and investment of the NHM, is concerning. Rather than attempting to bring many thousand facilities up to standards, regionalization of obstetric care and quality improvement in high-volume

For peer review only - http://bmjopen.bmj.com/site/about/guidelines.xhtml

facilities may offer a more efficient resource allocation strategy for the general population. Building on the success of NRHM in using supply- and demand-side strategies to reduce barriers to facility-based delivery [47], India may be particularly well positioned to assess the feasibility of regionalizing care. Assessment of regionalization should include checks against over-medicalization of childbirth care, which has been seen in some states [48]. That said, it is essential to strengthen lower-level facilities and improve referral linkages with higher-level facilities in areas with highly remote, rural and/or vulnerable populations, to ensure continued access to services for all, especially considering evidence from India indicating preference for PHCs when they are adequate [45, 49].

Our study has several limitations. First, it would have been ideal to examine the quality of clinical care provided to women in public health facilities such as the use of basic emergency care procedures for mothers and newborns (signal functions) and adherence to global guidelines for routine deliveries [35, 50]. Our index was constructed from indicators of basic equipment and service availability, measuring facility level inputs to care and basic service readiness, but it lacked information on important technical and interpersonal care processes, such as provider competence. Previous studies have noted the importance of provider knowledge and effort in determining the quality of actual clinical encounters [51] as well as substantial variation in the skill and competence of skilled birth attendants [52]. Likewise, although disrespectful care during childbirth is common [53] and respectful care is identified as WHO as a key requirement for improving quality of intrapartum care [39], we could not assess interpersonal care aspects

For peer review only - http://bmjopen.bmj.com/site/about/guidelines.xhtml

in our index. Critically, data constrained our ability to assess private facilities, which are also relied on for intrapartum care and may have different adherence to IPHS standards. We were also not able to assess quality of referral care or the outcomes of care. Thus, our measure should be seen as a starting point in measuring care quality in India; process and outcome quality may well be worse than the largely input-based measures examined here. Finally, these findings may not be representative of the six states and union territories without the survey data.

Our study findings add to the growing body of literature documenting gaps in provision of quality intrapartum care in LMICs as well as inadequate measurement to assess health system quality. For fifteen out of forty-eight intrapartum care items on the IPHS, we did not find any corresponding survey question in the DLHS facility audit. Harmonizing data collection with national standards is a critical first step towards addressing information gaps. Likewise, expanding data collection to capture the nature and content of care being provided is important for understanding quality of care [54]. Quality of care is complex and multifaceted. While the inputs to care measured here are a critical foundation for high-quality care, they are a poor proxy of care as delivered [55]. Even measures of process quality may be insufficient to predict quality-sensitive outcomes such as morbidity and mortality [10, 56, 57], particularly for childbirth where averting severe outcomes requires timely recognition of complications by qualified personnel and functional referral systems to ensure a rapid response [10, 57].

For peer review only

For peer review only - http://bmjopen.bmj.com/site/about/guidelines.xhtml

Future assessments should better capture the context of care and whether women's rights to quality care are being met, with a focus on clinical effectiveness of care, patient experience, respectful care, and outcomes [32]. A variety of methods are available for this, including patient and provider interviews, direct observations, and clinical vignettes; assessment of the private sector is essential to gaining a full understanding of health system performance [51]. Strengthening the publicly available health information system in India to collect reliable, frequent and timely data on facility quality is key for effective program implementation and for meeting national and global targets such as those of the Ending Preventable Maternal Mortality, the Every Newborn Action Plan, and the SDGs. It is also essential to link quality measures to health outcomes and demographic characteristics of the population to assess the determinants of quality and its distribution across population subgroups. Health systems research in India needs to be expanded to address the large knowledge gaps in health system quality [58].

Conclusion

Primary care facilities in India are not well prepared to provide high quality obstetric and newborn care, and facility capacity is worst in states with the worst health outcomes. Over the past decade, India's health system has operated in an extremely resource-constrained environment: from 2004-2014, government health expenditure has remained approximately around 1% of country's GDP [20]. The Indian government will need to increase investment in the health system, in providers, and in research to harness the full benefit of its public health infrastructure. Research on regionalization is a priority as this may offer an innovative approach to ensuring quality services for mothers and newborns.

For peer review only

The impact of regionalization strategies on facility overcrowding, performance incentive structures for frontline workers, as well as equity in service access should be important considerations of such research. Improving quality of care and strengthening public health infrastructure is integral to India's path to universal health coverage: without an explicit focus on quality, a push towards universal coverage is unlikely to lead to better health for mothers and newborns.

For peer review only

References

1. UN Development Program. Sustainable Development Goals. Geneva, United Nations: 2015.
2. The Partnership for Maternal Newborn & Child Health. A Global Review of the Key Interventions Related to Reproductive, Maternal, Newborn and Child Health (RMNCH). Geneva, Switzerland: PMNCH: 2011.
3. Dickson KE, Kinney MV, Moxon SG, Ashton J, Zaka N, Simen-Kapeu A, et al. Scaling up quality care for mothers and newborns around the time of birth: an overview of methods and analyses of intervention-specific bottlenecks and solutions. BMC pregnancy and childbirth. 2015;15 Suppl 2:S1. Epub 2015/09/24. doi: 10.1186/1471-2393-15-s2-s1. PubMed PMID: 26390820; PubMed Central PMCID: PMCPmc4578819.
4. Bhutta ZA, Das JK, Bahl R, Lawn JE, Salam RA, Paul VK, et al. Can available interventions end preventable deaths in mothers, newborn babies, and stillbirths, and at what cost? Lancet. 2014;384(9940):347-70. doi: 10.1016/S0140-6736(14)60792-3. PubMed PMID: 24853604.
5. Kruk ME, Larson E, Twum-Danso NA. Time for a quality revolution in global health. The Lancet Global health. 2016;4(9):e594-6. Epub 2016/08/20. doi: 10.1016/s2214-109x(16)30131-0. PubMed PMID: 27539798.
6. Sobel HL, Huntington D, Temmerman M. Quality at the centre of universal health coverage. Health Policy Plan. 2016;31(4):547-9. Epub 2015/10/01. doi: 10.1093/heapol/czv095. PubMed PMID: 26420642.
7. Nandan D. National rural health mission: turning into reality. Indian J Community Med. 2010;35(4):453-4. doi: 10.4103/0970-0218.74338. PubMed PMID: 21278861; PubMed Central PMCID: PMCPMC3026119.
8. National Health Mission. Five years (2009–2014). Achievements and new initiatives. New Delhi: Ministry of Health and Family Welfare, Government of India, 2014.
9. Lim SS, Dandona L, Hoisington JA, James SL, Hogan MC, Gakidou E. India's Janani Suraksha Yojana, a conditional cash transfer programme to increase births in health facilities: an impact evaluation. Lancet. 2010;375(9730):2009-23. Epub 2010/06/24. doi: 10.1016/s0140-6736(10)60744-1. PubMed PMID: 20569841.
10. Powell-Jackson T, Mazumdar S, Mills A. Financial incentives in health: New evidence from India's Janani Suraksha Yojana. J Health Econ. 2015;43:154-69. doi: 10.1016/j.jhealeco.2015.07.001. PubMed PMID: 26302940.
11. Ng M, Misra A, Diwan V, Agnani M, Levin-Rector A, De Costa A. An assessment of the impact of the JSY cash transfer program on maternal mortality reduction in Madhya Pradesh, India. Glob Health Action. 2014;7:24939. doi: 10.3402/gha.v7.24939. PubMed PMID: 25476929; PubMed Central PMCID: PMCPMC4256523.
12. Million Death Study C. Changes in cause-specific neonatal and 1-59-month child mortality in India from 2000 to 2015: a nationally representative survey. Lancet. 2017. doi: 10.1016/S0140-6736(17)32162-1. PubMed PMID: 28939096.
13. Leslie HH, Fink G, Nsona H, Kruk ME. Obstetric Facility Quality and Newborn Mortality in Malawi: A Cross-Sectional Study. PLoS medicine.

For peer review only - http://bmjopen.bmj.com/site/about/guidelines.xhtml

2016;13(10):e1002151. Epub 2016/10/19. doi: 10.1371/journal.pmed.1002151. PubMed PMID: 27755547; PubMed Central PMCID: PMC5068819.

14. Knight HE, Self A, Kennedy SH. Why are women dying when they reach hospital on time? A systematic review of the 'third delay'. PLoS One. 2013;8(5):e63846. Epub 2013/05/25. doi: 10.1371/journal.pone.0063846. PubMed PMID: 23704943; PubMed Central PMCID: PMCPMC3660500.

15. Patel R, Ladusingh L. Do Physical Proximity and Availability of Adequate Infrastructure at Public Health Facility Increase Institutional Delivery? A Three Level Hierarchical Model Approach. PLoS One. 2015;10(12). doi: 10.1371/journal.pone.0144352. PubMed PMID: 26689199; PubMed Central PMCID: PMCPMC4686327.

16. Kumar S, Dansereau E. Supply-side barriers to maternity-care in India: a facility-based analysis. PLoS One. 2014;9(8):e103927. Epub 2014/08/06. doi: 10.1371/journal.pone.0103927. PubMed PMID: 25093729; PubMed Central PMCID: PMCPMC4122393.

17. National Sample Survey Office, Ministry of Statistics and Programme Implementation. Key Indicators of Social Consumption in India: Health. New Delhi, India: Government of India, 2014.

18. Office of the Registrar General & Census Commissioner, Institute of Economic Growth. Annual Health Survey Report. New Delhi, India: Ministry of Home Affairs, Government of India, 2015.

19. International Institute for Population Sciences. District Level Household and Facility Survey - 4 State Fact Sheet (2012-13). Mumbai: Ministry of Health and Family Welfare, Government of India, 2014.

20. Patel V, Parikh R, Nandraj S, Balasubramaniam P, Narayan K, Paul VK, et al. Assuring health coverage for all in India. Lancet. 2015;386(10011):2422-35. doi: 10.1016/S0140-6736(15)00955-1. PubMed PMID: 26700532.

21. Dasgupta R, Bisht R. The Missing Mission in Health. Economic and Political Weekly. 2010;XLV(6):3.

22. Bhaumik S. India outlines plans for National Urban Health Mission. Lancet. 2012;380(9841):550. PubMed PMID: 22891372.

23. Programme Evaluation Organisation. Evaluation Study of National Rural Health Mission (NRHM) in Seven States. New Delhi, India: Planning Commission, Government of India, 2011.

24. National Health Mission, Ministry of Health & Family Welfare, Government of India. Indian Public Health Standards New Delhi, India: Government of India; [cited 2016 November 15]. Available from: http://nrhm.gov.in/nhm/nrhm/guidelines/indian-public-health-standards.html.

25. Ministry of Health and Family Welfare. Rural health statistics bulletin 2015. New Delhi: Government of India, 2015.

26. Zaman FA, Laskar NB. An application of Indian public health standard for evaluation of primary health centers of an EAG and a Non-EAG state. Indian journal of public health. 2010;54(1):36-9. Epub 2010/09/23. doi: 10.4103/0019-557x.70551. PubMed PMID: 20859050.

27. Sodani P, Sharma K. Assessing Indian Public Health Standards for 24 × 7 primary health centers: A case study with special reference to newborn care

For peer review only - http://bmjopen.bmj.com/site/about/guidelines.xhtml

services. The Journal of National Accreditation Board for Hospitals & Healthcare Providers. 2014;1(1):12-6. doi: 10.4103/2348-6139.132919.
28. Sodani PR, Sharma K. Assessing Indian public health standards for community health centers: a case study with special reference to essential newborn care services. Indian journal of public health. 2011;55(4):260-6. Epub 2012/02/03. doi: 10.4103/0019-557x.92402. PubMed PMID: 22298134.
29. Sodani PR, Sharma K. Strengthening Primary Level Health Service Delivery: Lessons from a State in India. Journal of Family Medicine and Primary Care. 2012;1(2):127-31. doi: 10.4103/2249-4863.104983. PubMed PMID: 24479021; PubMed Central PMCID: PMCPMC3893968.
30. Directorate General of Health Services. Indian Public Health Standards (IPHS) Guidelines for Primary Health Centers. New Delhi: Ministry of Health and Family Welfare, Government of India, 2012.
31. Bhatia M. The Indian Helath Care System, 2015. United States: The Commonwealth Fund, 2016.
32. Tuncalp, Were WM, MacLennan C, Oladapo OT, Gulmezoglu AM, Bahl R, et al. Quality of care for pregnant women and newborns-the WHO vision. BJOG. 2015;122(8):1045-9. doi: 10.1111/1471-0528.13451. PubMed PMID: 25929823; PubMed Central PMCID: PMCPMC5029576.
33. Donabedian A. The quality of care. How can it be assessed? JAMA. 1988;260(12):1743-8. PubMed PMID: 3045356.
34. Kruk ME, Leslie HH, Verguet S, Mbaruku GM, Adanu RM, Langer A. Quality of basic maternal care functions in health facilities of five African countries: an analysis of national health system surveys. The Lancet Global health. 2016;4(11):e845-e55. doi: 10.1016/S2214-109X(16)30180-2. PubMed PMID: 27670090.
35. Tripathi V, Stanton C, Strobino D, Bartlett L. Development and Validation of an Index to Measure the Quality of Facility-Based Labor and Delivery Care Processes in Sub-Saharan Africa. PLoS One. 2015;10(6):e0129491. Epub 2015/06/25. doi: 10.1371/journal.pone.0129491. PubMed PMID: 26107655; PubMed Central PMCID: PMC4479466.
36. Hsia RY, Mbembati NA, Macfarlane S, Kruk ME. Access to emergency and surgical care in sub-Saharan Africa: the infrastructure gap. Health Policy Plan. 2012;27(3):234-44. doi: 10.1093/heapol/czr023. PubMed PMID: 21441566.
37. WHO, UNFPA, UNICEF, Mailman School of Public Health. Monitoring emergency obstetric care: a handbook. Geneva: World Health Organization, 2009.
38. Rao M, Rao KD, Kumar AK, Chatterjee M, Sundararaman T. Human resources for health in India. Lancet. 2011;377(9765):587-98. doi: 10.1016/S0140-6736(10)61888-0. PubMed PMID: 21227499.
39. World Health Organization. Standard for improving quality of maternal and newborn care in health facilities. Geneva, Switzerland: World Health Organization Press; 2016.
40. Balarajan Y, Selvaraj S, Subramanian SV. Health care and equity in India. Lancet. 2011;377(9764):505-15. doi: 10.1016/S0140-6736(10)61894-6. PubMed PMID: 21227492; PubMed Central PMCID: PMCPMC3093249.

For peer review only - http://bmjopen.bmj.com/site/about/guidelines.xhtml

41. Barik D, Thorat A. Issues of Unequal Access to Public Health in India. Front Public Health. 2015;3:245. doi: 10.3389/fpubh.2015.00245. PubMed PMID: 26579507; PubMed Central PMCID: PMCPMC4621381.
42. Gupta A, Fledderjohann J, Reddy H, Raman VR, Stuckler D, Vellakkal S. Barriers and prospects of India's conditional cash transfer program to promote institutional delivery care: a qualitative analysis of the supply-side perspectives. BMC Health Serv Res. 2018;18(1):40. Epub 2018/01/27. doi: 10.1186/s12913-018-2849-8. PubMed PMID: 29370798; PubMed Central PMCID: PMCPMC5785836.
43. Kruk ME, Hermosilla S, Larson E, Mbaruku GM, Kruk ME, Hermosilla S, et al. Bypassing primary care clinics for childbirth: a cross-sectional study in the Pwani region, United Republic of Tanzania. Bulletin of the World Health Organization. 2014;92(4):246-53.
44. Prinja S, Gupta A, Verma R, Bahuguna P, Kumar D, Kaur M, et al. Cost of Delivering Health Care Services in Public Sector Primary and Community Health Centres in North India. PLoS One. 2016;11(8):e0160986. doi: 10.1371/journal.pone.0160986. PubMed PMID: 27536781; PubMed Central PMCID: PMCPMC4990301.
45. Jayanthi TP, Suresh S, Padmanaban P. Primary health centres: preferred option for birthing care in tamilnadu, India, from users' perspectives. J Health Popul Nutr. 2015;33(1):177-86. Epub 2015/05/23. PubMed PMID: 25995734; PubMed Central PMCID: PMCPMC4438661.
46. Straneo M, Hanson C, Fogliati P, Mbaruku GM. Minimum obstetric volume in low-income countries. The Lancet. 2017;389(10070):698. doi: 10.1016/S0140-6736(17)30342-2.
47. Randive B, Diwan V, De Costa A. India's Conditional Cash Transfer Programme (the JSY) to Promote Institutional Birth: Is There an Association between Institutional Birth Proportion and Maternal Mortality? PLoS One. 2013;8(6):e67452. Epub 2013/07/05. doi: 10.1371/journal.pone.0067452. PubMed PMID: 23826302; PubMed Central PMCID: PMC3694862.
48. Leone T. Demand and supply factors affecting the rising overmedicalization of birth in India. Int J Gynaecol Obstet. 2014;127(2):157-62. Epub 2014/07/30. doi: 10.1016/j.ijgo.2014.05.018. PubMed PMID: 25064013.
49. Vellakkal S, Reddy H, Gupta A, Chandran A, Fledderjohann J, Stuckler D. A qualitative study of factors impacting accessing of institutional delivery care in the context of India's cash incentive program. Soc Sci Med. 2017;178:55-65. Epub 2017/02/16. doi: 10.1016/j.socscimed.2017.01.059. PubMed PMID: 28199860; PubMed Central PMCID: PMCPMC5360172.
50. World Health Organization. WHO safe childbirth checklist implementation guide: improving the quality of facility-based delivery for mothers and newborns. Geneva: World Health Organization, 2015.
51. Das J, Hammer J. Quality of Primary Care in Low-Income Countries: Facts and Economics. Annual Review of Economics. 2014;6(1):525-53. doi: doi:10.1146/annurev-economics-080213-041350.
52. Harvey SA, Blandon YC, McCaw-Binns A, Sandino I, Urbina L, Rodriguez C, et al. Are skilled birth attendants really skilled? A measurement method, some disturbing results and a potential way forward. Bull World Health Organ.

For peer review only - http://bmjopen.bmj.com/site/about/guidelines.xhtml

2007;85(10):783-90. PubMed PMID: 18038060; PubMed Central PMCID: PMCPMC2636500.

53. Bohren MA, Vogel JP, Hunter EC, Lutsiv O, Makh SK, Souza JP, et al. The Mistreatment of Women during Childbirth in Health Facilities Globally: A Mixed-Methods Systematic Review. PLoS medicine. 2015;12(6):e1001847; discussion e. Epub 2015/07/01. doi: 10.1371/journal.pmed.1001847. PubMed PMID: 26126110; PubMed Central PMCID: PMCPmc4488322.

54. Campbell OM, Calvert C, Testa A, Strehlow M, Benova L, Keyes E, et al. The scale, scope, coverage, and capability of childbirth care. Lancet. 2016. doi: 10.1016/S0140-6736(16)31528-8. PubMed PMID: 27642023.

55. Leslie HH, Sun Z, Kruk ME. Association between infrastructure and observed quality of care in 4 healthcare services: A cross-sectional study of 4,300 facilities in 8 countries. PLoS medicine. 2017;14(12):e1002464. doi: 10.1371/journal.pmed.1002464.

56. Souza JP, Gülmezoglu AM, Vogel J, Carroli G, Lumbiganon P, Qureshi Z, et al. Moving beyond essential interventions for reduction of maternal mortality (the WHO Multicountry Survey on Maternal and Newborn Health): a cross-sectional study. The Lancet. 2013;381(9879):1747-55 %U http://www.sciencedirect.com/science/article/pii/S0140673613606868.

57. Semrau KEA, Hirschhorn LR, Marx Delaney M, Singh VP, Saurastri R, Sharma N, et al. Outcomes of a Coaching-Based WHO Safe Childbirth Checklist Program in India. New England Journal of Medicine. 2017;377(24):2313–24. doi: 10.1056/NEJMoa1701075.

58. Das J, Mohpal A. Socioeconomic Status And Quality Of Care In Rural India: New Evidence From Provider And Household Surveys. Health Aff (Millwood). 2016;35(10):1764-73. doi: 10.1377/hlthaff.2016.0558. PubMed PMID: 27702947.

For peer review only - http://bmjopen.bmj.com/site/about/guidelines.xhtml

Table 1: Indian Public Health Standard for basic delivery and newborn care, and corresponding question (closest match) in the DLHS-4 facility survey for Primary and Community Health Centers

Indian Public Health Standard	DLHS Question	Data Type	Categorization
**Management of normal deliveries**	Can you tell me whether normal delivery services are provided in this facility?	Self-reported	-
**Assisted vaginal deliveries including forceps/vacuum delivery whenever required**	Can you tell me whether assisted (forceps delivery/vacuum) delivery services are provided in this facility?	Self-reported	Process
**24-hour delivery services both normal and assisted**	Whether deliveries are conducted in this facility or no? If yes, whether the deliveries are conducted 24X7?	Self-reported	-
**Manual removal of placenta**	NA		
**Appropriate & prompt referral for cases needing specialist care**	NA		
**Management of pregnancy induced hypertension & referral**	NA		
**Pre-referral management (obstetric first-aid) in obstetric emergencies that need expert assistance**	NA		
**Proficient in identification and basic first aid treatment for PPH, eclampsia, sepsis and prompt referral**	Can you tell me whether administration of parenteral antibiotics is provided in this facility?	Self-reported	Process
	Can you tell me whether administration of parenteral oxytocics is provided in this facility?	Self-reported	Process
	Can you tell me whether administration of parenteral magnesium sulphate is provided in this facility?	Self-reported	Process
	Can you tell me whether management of PPH is provided in this facility?	Self-reported	Process
**Minimum 48 hours of stay after delivery**	NA		
**Facilities for Essential New Born Care and Resuscitation (Newborn Care Corner in LR/OT)**	Whether the following essential newborn care services are available? Resuscitation	Self-reported	Process
	Availability of designated newborn baby corner (functional)	Observed	-

For peer review only - http://bmjopen.bmj.com/site/about/guidelines.xhtml

**Management of neonatal hypothermia, infection protection, cord care & identification of sick newborn & prompt referral**	Whether the following essential newborn care services are available? Thermal protection (warmer/table lamp)	Self-reported	Process
**Early initiation of breastfeeding within one hour of birth**	NA		
**At least one medical officer - MBBS**	Availability of human resources (Medical Officer - Regular) - No. in position	Self-reported	Structure: human resources
**Three nurse midwife (staff-nurse)**	Availability of human resources (ANM/Female Health Worker - Regular) - No. in position	Self-reported	Structure: human resources
**Health worker female**	NA		
**Health Assistant (female)**	NA		
**Training for staff for emergency management to be ensured**	Training received by any medical officer during last five years (SBA or Basic Emergency Obstetric Care training)	Self-reported	Structure: human resources
	Training received for paramedical staff during last five years (SBA training)	Self-reported	Structure: human resources
**Labor table**	Physically verify and record availability of labor table with McIntosh sheet	Observed	Structure: facilities and supplies for functional labor room
**Managing labor using partograph**	Partographs being recorded for the recently delivered women or women in labor at the facility	Self-reported	Structure: facilities and supplies for functional labor room
**Suction machine**	Physically verify and record availability of suction machine (functional)	Observed	Structure: facilities and supplies for functional labor room
**Facility for oxygen administration**	NA		Structure: facilities and supplies for functional labor room
**Sterilization equipment (autoclave)**	Physically verify and record availability of autoclave/sterilizer (functional)	Observed	Structure: facilities and supplies for functional labor room
**24-hour running water**	Physically verify and record availability of 24 hour running water supply	Observed	Structure: facilities and supplies for functional labor room
**Electricity supply with back-up facility**	Whether the generator supply is connected to the LR	Observed	Structure: facilities and

For peer review only - http://bmjopen.bmj.com/site/about/guidelines.xhtml

**(generator with POL)**			supplies for functional labor room
**Attached toilet facilities**	Physically verify and record availability of attached toilet in the LR (functional)	Observed	Structure: facilities and supplies for functional labor room
**Delivery kits, including those for normal & assisted deliveries**	Normal delivery kits available in the facility?	Self-reported	Structure: facilities and supplies for functional labor room
**Privacy of woman ensured**	Observe and record the condition of the LR - privacy in the LR (satisfactory)	Observed	Structure: facilities and supplies for functional labor room
**Radiant warmer, fixed height with trolley**	Radiant warmer	Observed	Structure: equipment and supplies for newborn care
**Resuscitation bag and mask with reservoir**	Ambu bag with mask (functional)	Observed	Structure: equipment and supplies for newborn care
**Weighing scale**	Baby weighing machine of any time (functional)	Observed	Structure: equipment and supplies for newborn care
**Thermometer, clinical, digital**	NA		Structure: equipment and supplies for newborn care
**Light examination**	NA		Structure: equipment and supplies for newborn care
**I/V Cannula 24G, 26G**	Suction catheter/cannula (functional)	Observed	Structure: equipment and supplies for newborn care
**Mucus extractor; Pump suction, foot operated**	Pedal suction machine/mucus extractor (functional)	Observed	Structure: equipment and supplies for newborn care
**Feeding tube**	NA		
**Oxygen catheter**	NA		
**Sterile gloves**	Whether following emergency drugs and consumables are available: at least 2 pairs of gloves	Self-reported	Structure: emergency drugs and supplies for labor and delivery
**Inj. Oxytocin**	Whether following emergency drugs and consumables are available: oxytocin injection	Self-reported	Structure: emergency drugs and supplies for labor and delivery
**Inj. Diasepam**	Whether following emergency drugs and consumables are available: diazepam injection	Self-reported	Structure: emergency drugs and supplies for labor and delivery

For peer review only - http://bmjopen.bmj.com/site/about/guidelines.xhtml

**Tab. Nifedepine**	Whether following emergency drugs and consumables are available: nifedipine tablet	Self-reported	Structure: emergency drugs and supplies for labor and delivery
**Inj. Magnesium Sulphate**	Whether following emergency drugs and consumables are available: magnesium sulphate injection	Self-reported	Structure: emergency drugs and supplies for labor and delivery
**Inj. Lignocaine hydrochloride**	Whether following emergency drugs and consumables are available: lignocaine hydrochloride injection	Self-reported	Structure: emergency drugs and supplies for labor and delivery
**Inj. Methyl ergometrine maleate**	NA		Structure: emergency drugs and supplies for labor and delivery
**I/V Haemaccel**	NA		Structure: emergency drugs and supplies for labor and delivery
**Sterilized cotton and gauze**	Whether following emergency drugs and consumables are available: sterilized cotton and gauze	Self-reported	Structure: emergency drugs and supplies for labor and delivery
**Syringe**	Whether following emergency drugs and consumables are available: sterile syringes and needles (different sizes)	Self-reported	Structure: emergency drugs and supplies for labor and delivery
**Hub cutter**	Hub Cutter (available and functional)	Observed	Structure: emergency drugs and supplies for labor and delivery
**Oxygen bottles**	Whether following emergency drugs and consumables are available: Oxygen cylinder with face mask, wrench & regulator - functional	Self-reported	Structure: emergency drugs and supplies for labor and delivery

Indicators were extracted from Indian Public Health Standards (IPHS) for Primary Health Center;
DLHS questions from both Primary and Community Health Center surveys;
LR: Labor Room;
NA refers to IPHS indicators that were not included in the DLHS facility survey.

For peer review only - http://bmjopen.bmj.com/site/about/guidelines.xhtml

Figure Titles and Legends

Figure 1: Availability of basic delivery and newborn services in primary care facilities in India

Figure 2: Capacity of Primary Health Centers and Community Health Centers for basic delivery and newborn care

*** Legend:
Overall facility capacity of basic delivery and newborn care calculated as the average of the two preceding summary measures (process and structural capacity). Details on indicators of structure are available in Supplement Figure 1. Differences between PHC and CHC, for all indicators and summary index are statistically significant at $p<0.05$ level (Pearson's chi-square test and Student's t-test).

Figure 3: Difference in facility capacity for basic delivery and newborn care across rural and urban Primary Health Centers

***Legend: Differences between rural and urban facilities for all three summary indices are not statistically significant at $p<0.05$ level (Student's t-test).

Figure 4: Difference in facility capacity for basic delivery and newborn care across rural and urban Community Health Centers

Figure 5: Empowered Action Group (EAG) status and facility capacity for basic delivery and newborn care

Supplement Figure 1: Indicators of basic structural capacity for delivery and newborn care in primary facilities

Supplement Figure 2: Mean facility capacity scores in Empowered Action Group (EAG) states

***Legend: Mean facility capacity scores were calculated by summarizing overall capacity at district level, weighting facilities by total deliveries

Supplement Figure 3: Mean facility capacity scores in non-EAG states

***Legend: Mean facility capacity scores were calculated by summarizing overall capacity at district level, weighting facilities by total deliveries

For peer review only

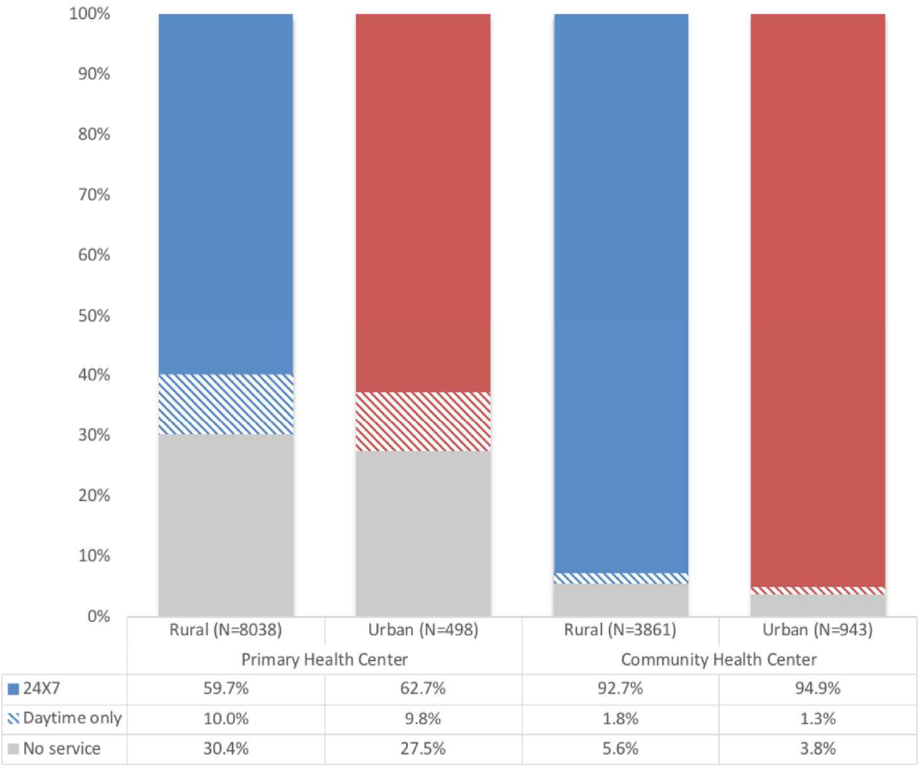

Figure 1: Availability of basic delivery and newborn services in primary care facilities in India

333x296mm (300 x 300 DPI)

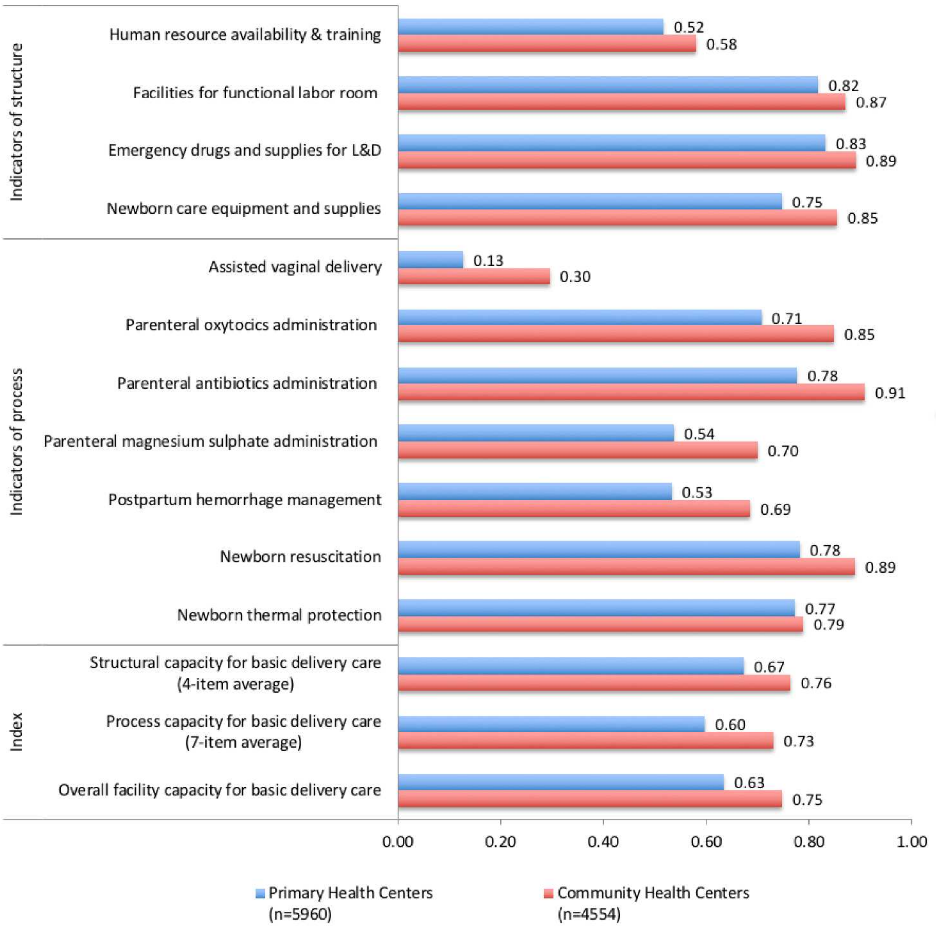

Figure 2: Capacity of Primary Health Centers and Community Health Centers for basic delivery and newborn care

*** Legend:
Overall facility capacity of basic delivery and newborn care calculated as the average of the two preceding summary measures (process and structural capacity). Details on indicators of structure are available in Supplement Figure 1. Differences between PHC and CHC, for all indicators and summary index are statistically significant at $p<0.05$ level (Pearson's chi-square test and Student's t-test).

333x351mm (300 x 300 DPI)

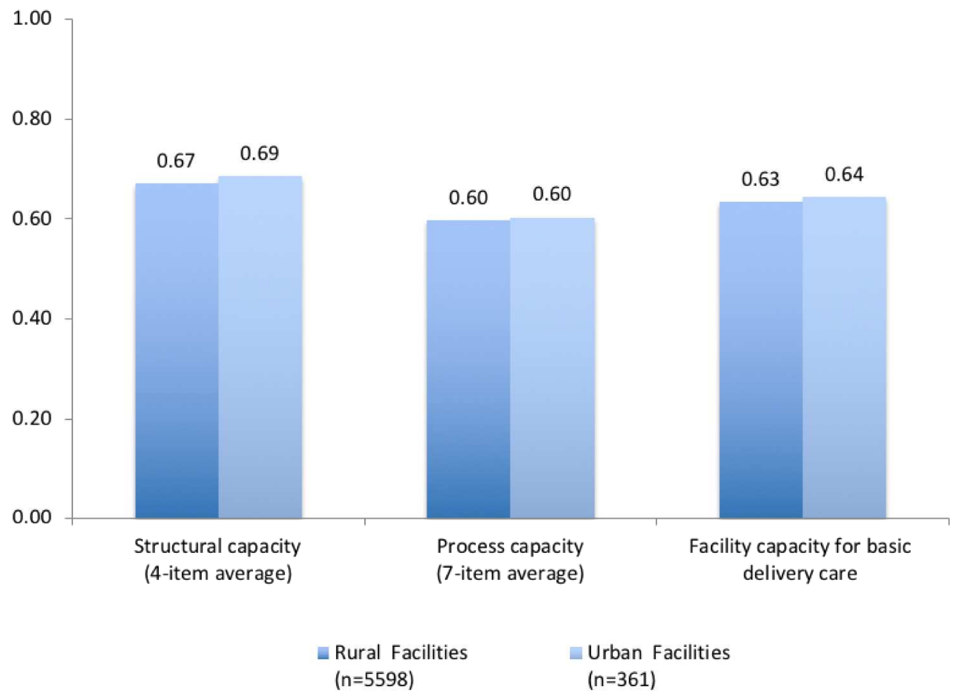

Figure 3: Difference in facility capacity for basic delivery and newborn care across rural and urban Primary Health Centers

***Legend: Differences between rural and urban facilities for all three summary indices are not statistically significant at p<0.05 level (Student's t-test).

288x217mm (300 x 300 DPI)

only

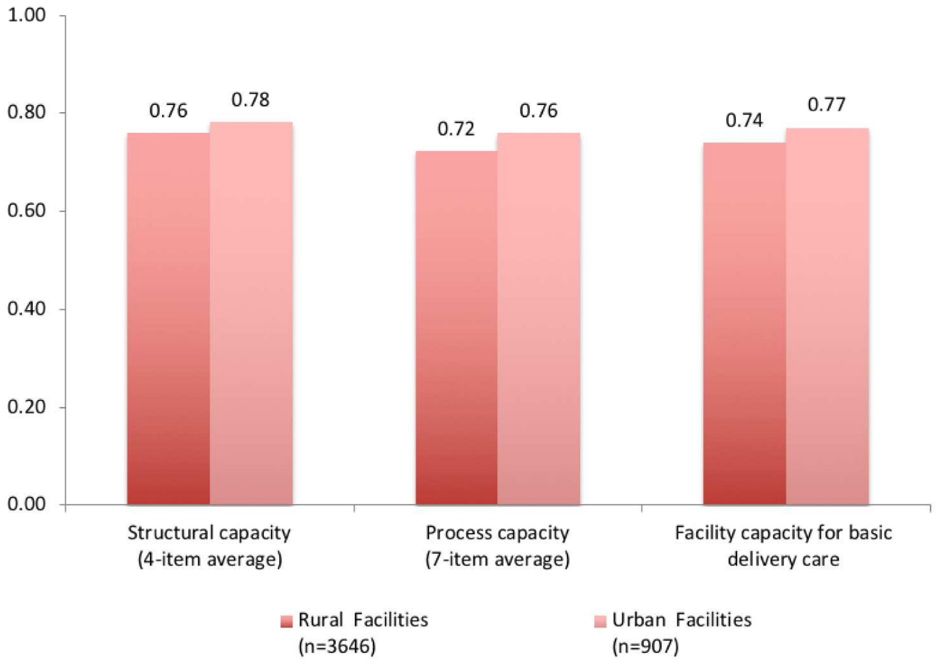

Figure 4: Difference in facility capacity for basic delivery and newborn care across rural and urban Community Health Centers

294x216mm (300 x 300 DPI)

w only

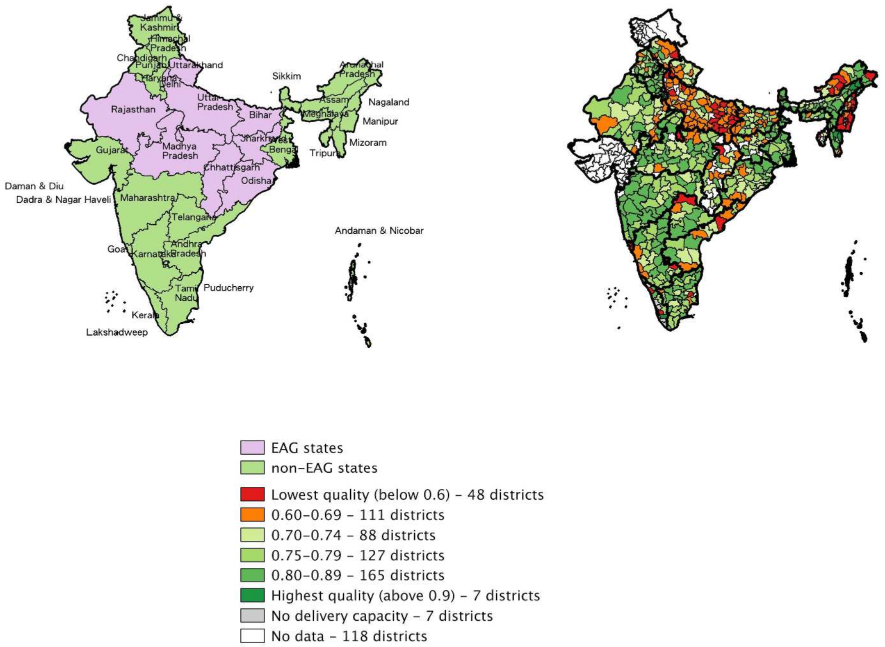

Figure 5: Empowered Action Group (EAG) status and facility capacity for basic delivery and newborn care

297x210mm (300 x 300 DPI)

ew only

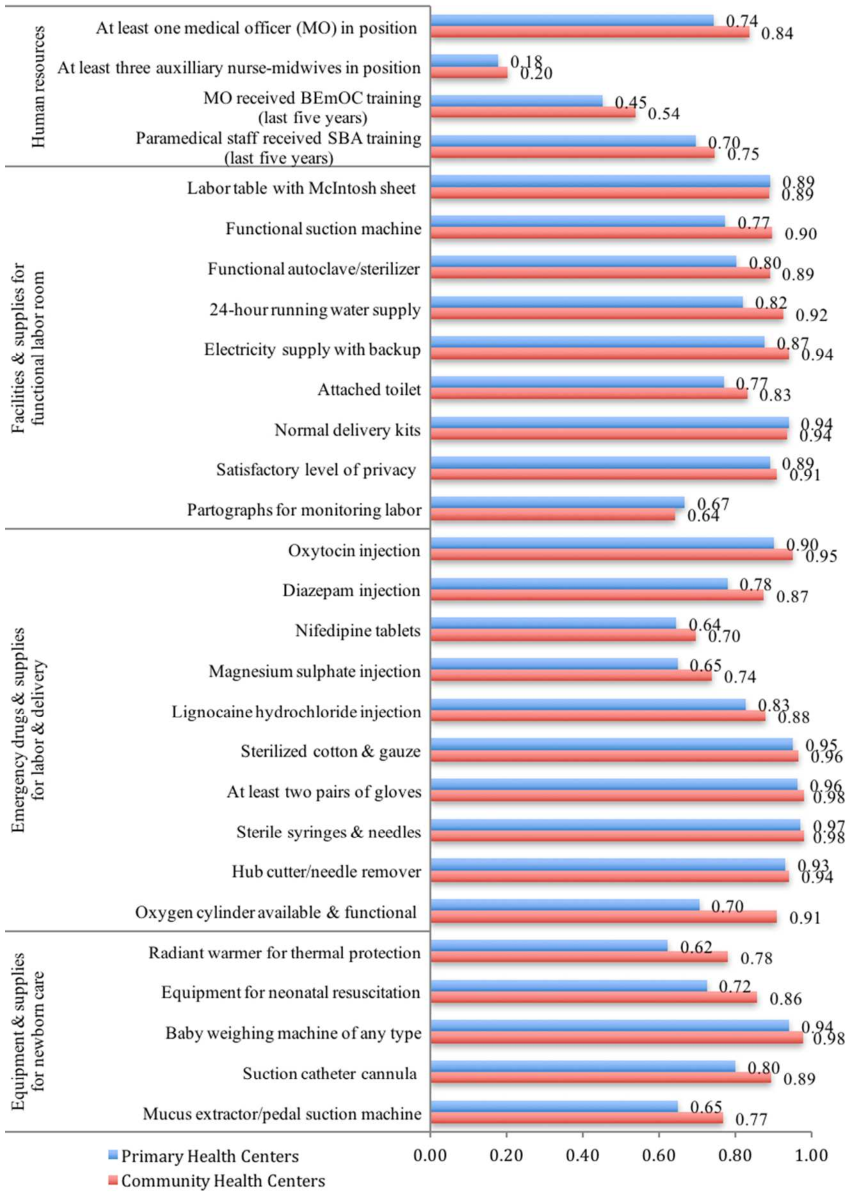

Supplement Figure 1: Indicators of basic structural capacity for delivery and newborn care in primary facilities

76x107mm (300 x 300 DPI)

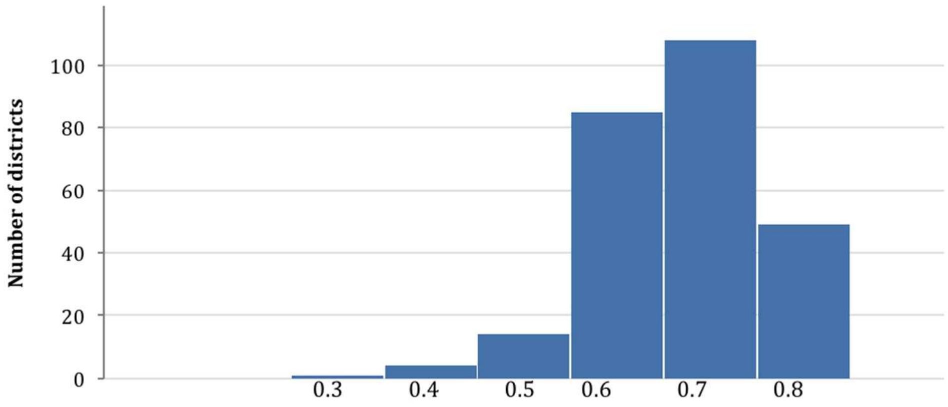

Supplement Figure 2: Mean facility capacity scores in Empowered Action Group (EAG) states

***Legend: Mean facility capacity scores were calculated by summarizing overall capacity at district level, weighting facilities by total deliveries

84x35mm (300 x 300 DPI)

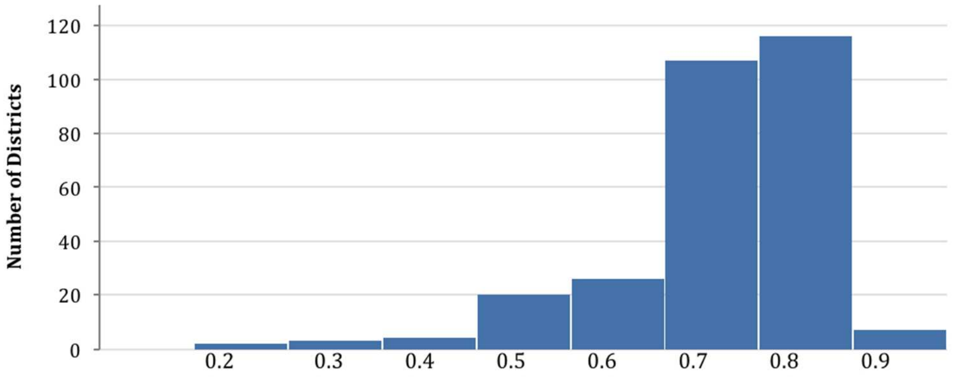

Supplement Figure 3: Mean facility capacity scores in non-EAG states

***Legend: Mean facility capacity scores were calculated by summarizing overall capacity at district level, weighting facilities by total deliveries

87x34mm (300 x 300 DPI)

For peer review only

STROBE Statement—Checklist of items that should be included in reports of ***cross-sectional studies***

	Item No	Recommendation	Page No. and Line No.
**Title and abstract**	1	(*a*) Indicate the study's design with a commonly used term in the title or the abstract	Page 1, lines 1-2
		(*b*) Provide in the abstract an informative and balanced summary of what was done and what was found	Pages 2-3
**Introduction**			
Background/rationale	2	Explain the scientific background and rationale for the investigation being reported	Pages 6-9
Objectives	3	State specific objectives, including any prespecified hypotheses	Page 9, lines 6-12
**Methods**			
Study design	4	Present key elements of study design early in the paper	Pages 9 - 12
Setting	5	Describe the setting, locations, and relevant dates, including periods of recruitment, exposure, follow-up, and data collection	Page 9 line 16 – page 10 line 9
Participants	6	(*a*) Give the eligibility criteria, and the sources and methods of selection of participants	Page 9 line 16 – page 10 line 9
Variables	7	Clearly define all outcomes, exposures, predictors, potential confounders, and effect modifiers. Give diagnostic criteria, if applicable	Page 10 line 11 – page 12 line 18
Data sources/ measurement	8*	For each variable of interest, give sources of data and details of methods of assessment (measurement). Describe comparability of assessment methods if there is more than one group	Pages 9 - 12
Bias	9	Describe any efforts to address potential sources of bias	NA
Study size	10	Explain how the study size was arrived at	Page 9 line 16 – 19
Quantitative variables	11	Explain how quantitative variables were handled in the analyses. If applicable, describe which groupings were chosen and why	Page 10 line 12 – Page12 line 18
Statistical methods	12	(*a*) Describe all statistical methods, including those used to control for confounding	Page 12 line 21 – Page 13 line 17
		(*b*) Describe any methods used to examine subgroups and interactions	Page 13 lines 10-17
		(*c*) Explain how missing data were addressed	Page 11 lines 1-5
		(*d*) If applicable, describe analytical methods taking account of sampling strategy	NA
		(*e*) Describe any sensitivity analyses	NA
**Results**			
Participants	13*	(a) Report numbers of individuals at each stage of study—eg numbers potentially eligible, examined for eligibility, confirmed eligible, included in the study, completing follow-up, and analysed	Page 14 line 5-22
		(b) Give reasons for non-participation at each stage	Page 14 line 5-22
		(c) Consider use of a flow diagram	NA
Descriptive data	14*	(a) Give characteristics of study participants (eg	Page 14 lines 17-22

For peer review only - http://bmjopen.bmj.com/site/about/guidelines.xhtml

		demographic, clinical, social) and information on exposures and potential confounders	
		(b) Indicate number of participants with missing data for each variable of interest	Page 14 lines 5-11
Outcome data	15*	Report numbers of outcome events or summary measures	Page 15 lines 1-7
Main results	16	(*a*) Give unadjusted estimates and, if applicable, confounder-adjusted estimates and their precision (eg, 95% confidence interval). Make clear which confounders were adjusted for and why they were included	Page 15 lines 1 – Page 16 line 11
		(*b*) Report category boundaries when continuous variables were categorized	Page 16, line 7-11
		(*c*) If relevant, consider translating estimates of relative risk into absolute risk for a meaningful time period	NA
Other analyses	17	Report other analyses done—eg analyses of subgroups and interactions, and sensitivity analyses	NA
**Discussion**			
Key results	18	Summarise key results with reference to study objectives	Page 16 line 14 – Page 17 line 2
Limitations	19	Discuss limitations of the study, taking into account sources of potential bias or imprecision. Discuss both direction and magnitude of any potential bias	Page 20 line 5 - 22
Interpretation	20	Give a cautious overall interpretation of results considering objectives, limitations, multiplicity of analyses, results from similar studies, and other relevant evidence	Page 22 line 7 – 20
Generalisability	21	Discuss the generalisability (external validity) of the study results	Page 20 line 16 – 22
**Other information**			
Funding	22	Give the source of funding and the role of the funders for the present study and, if applicable, for the original study on which the present article is based	Page 4 lines 18-20

For peer review only

For peer review only - http://bmjopen.bmj.com/site/about/guidelines.xhtml
